# Structural Features, Chemical Diversity, and Physical Properties of Microporous Sodalite-Type Materials: A Review

**DOI:** 10.3390/ijms251810218

**Published:** 2024-09-23

**Authors:** Nikita V. Chukanov, Sergey M. Aksenov

**Affiliations:** 1 Federal Research Center of Problems of Chemical Physics and Medicinal Chemistry, Russian Academy of Sciences, Chernogolovka 142432, Russia; 2 Faculty of Geology, Moscow State University, Moscow 119991, Russia; 3 Laboratory of Arctic Mineralogy and Material Sciences, Federal Research Center Kola Science Centre, Russian Academy of Sciences, Apatity 184209, Russia; 4 Geological Institute, Federal Research Center Kola Science Centre, Russian Academy of Sciences, Apatity 184209, Russia; 5 Institute of the Earth’s Crust, Siberian Branch, Russian Academy of Sciences, Irkutsk 664033, Russia

**Keywords:** sodalite, sodalite-related materials, synthesis, crystal chemistry, sorption, ion exchange, immobilization, catalysis, ion conductivity, chromophores, thermal conversions

## Abstract

This review contains data on a wide class of microporous materials with frameworks belonging to the sodalite topological type. Various methods for the synthesis of these materials, their structural and crystal chemical features, as well as physical and chemical properties are discussed. Specific properties of sodalite-related materials make it possible to consider they as thermally stable ionic conductors, catalysts and catalyst carriers, sorbents, ion exchangers for water purification, matrices for the immobilization of radionuclides and heavy metals, hydrogen and methane storage, and stabilization of chromophores and phosphors. It has been shown that the diversity of properties of sodalite-type materials is associated with the chemical diversity of their frameworks and extra-framework components, as well as with the high elasticity of the framework.

## 1. Introduction

**Figure 1:**
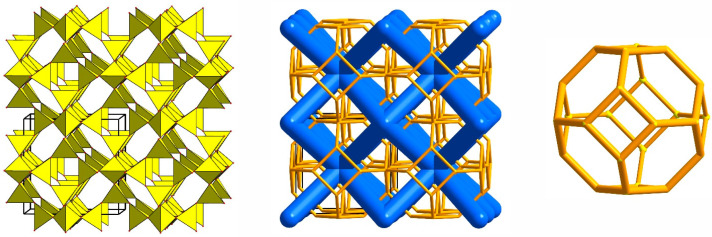
The general view of the SOD-type topology of the framework (coordination tetrahedra are shown in yellow) with the channels (blue lines) running through the SOD-cages.

With respect to the crystal structure, the mineral sodalite, Na_8_(Al_6_Si_6_O_24_)Cl_2_, is an ancestor of a large group of microporous materials with diverse physical and chemical properties, including technologically important ones. Its aluminosilicate framework has a cubic symmetry and is built by the successive alternation of layers containing six-membered rings of alternating Si- and Al-centered tetrahedra around the three-fold axis [1/3 2/3 *z*], [2/3 1/3 *z*], and [0 0 *z*]. The framework hosts intersecting zeolite channels running along three directions and consisting of so-called sodalite cages which are bounded by five six-membered rings of tetrahedra and six four-membered rings (Figure 1). The cages share common six-membered rings of tetrahedra and host Na^+^ and Cl^−^ ions forming [Na_4_Cl]^3+^ clusters.

Other minerals belonging to the sodalite group have aluminosilicate, beryllosilicate or ferrite frameworks. Among their major extra-framework components, there are various cations (Na^+^, K^+^, N(CH_3_)_4_^+^, Ca^2+^, Fe^2+^, Mn^2+^, Zn^2+^), anions (SO_4_^2−^, SO_3_^2−^, S^2−^, O^2−^, HS^−^, Cl^−^, F^−^, OH^−^), radical anion S_3_^•−^, and neutral molecules (S_6_, CO, H_2_O) [1,2,3,4,5,6,7,8,9,10,11,12,13,14,15,16,17,18,19,20,21,22], and some other extra-framework components (H^+^, H_3_O^+^, MoO_4_^2−^, WO_4_^2−^, AsO_4_^3−^, SO_4_^•−^, S_2_^•−^, *cis*- and *trans*-S_4_^•−^, *cis*- and *trans*-S_4_, COS) were detected in minor amounts using a multimethod approach based on electron microprobe analyses, electron spin resonance, infrared, Raman and photoluminescence spectroscopy, luminescence excitation, and absorption spectroscopy in visible, ultraviolet, and near infrared ranges [17,18,19,23,24,25,26,27].

Ordering of extra-framework components can result in a lowering of the framework symmetry to orthorhombic, monoclinic, or triclinic as well as structure modulations [28]. Some sodalite-group minerals are widely distributed in nature and belong to major components of rocks.

Chemical composition of synthetic sodalite-related compounds is more diverse. To date, a large number of compounds of the sodalite (SOD) structural type have been synthesized, differing both in the composition of the framework and in the set of extra-framework components. Some of them are considered as advanced materials which can be used as matrices for immobilization of heavy metals and radionuclides, storage of hydrogen, as membranes for separation of gases and desalination of seawater, sorbents for water purification, pigments, catalysts, superconductors, etc.

The topological type of sodalite is realized in compounds of various classes, which indicates its high stability. This conclusion is confirmed by numerous experimental data, which show high thermal and chemical stability of sodalite-type materials in various environments, unlike most zeolites. At present, the use of sodalite-like materials as sorbents, ionites, matrices for immobilization of radionuclides, and catalysts is limited by the lack of developed cheap technologies for obtaining such materials. The possibility of synthesizing sodalite from cheap raw materials has been demonstrated only in laboratory experiments. The purpose of this review is to summarize the available data on the synthesis, crystal-structure features, and physical and chemical properties of sodalite-related materials. We hope that this review will serve as an incentive for the development of such technologies.

As being the part of the “Women’s Special Issue Series: Recent Advances in Molecular Crystal Materials” we would like to dedicated it to three Russian women crystallographers (Prof. Nadezhda B. Bolotina, Prof. Ramiza K. Rastsvetaeva and Dr. Ekaterina V. Kaneva), who made significant contributions to the study of the structures and crystal chemistry of sodalite-group minerals.

## 2. Synthesis

### 2.1. Synthesis of Sodalite and Basic Sodalite

Natural sodalite crystallizes directly from Cl-enriched fluids or as a result of the conversion of primary framework aluminosilicates, nepheline, (Na,K)AlSiO_4_, or albite, NaAlSi_3_O_8_, under the action of Na-, Cl-, and Al-bearing basic fluids with low Si concentrations [29].

Sodalite was synthesized in the reaction of nepheline with alkaline brines at relatively high sodium chloride concentrations at 750 °C and high pressures of 3 to 6 kbar [30]. The reaction boundary was located at the NaCl:(NaCl + H_2_O) molar ratios of 0.16 and 0.35 at 3 and 6 kbar, respectively. However, in typical natural sodalite-forming media, such high NaCl concentrations are unlikely. The coexistence of nepheline and sodalite buffers the activity of NaCl in a coexisting aqueous solution to relatively low values at low pressures. There is no obvious evidence of incorporation of OH or H_2_O into the channels of the sodalite formed.

When developing methods for the synthesis of sodalite-related materials for the purpose of its use in various industrial technologies, the availability of significant reserves of cheap raw materials should be taken into account. In this case, in fact, as a rule, we are not talking about sodalite itself with Cl^−^ as an extra-framework anion, but about the so-called basic sodalite or “hydrated hydroxysodalite”, Na_6+*x*_(Al_6_Si_6_O_24_)(OH)*_x_*·*n*H_2_O [31]. In some sources, this material is described with the names “hydrosodalite”, or “hydroxysodalite”. However, these terms are inexact and should be applied to the sodalite-type compounds with the formulae Na_6_[AlSiO_4_]_6_·8H_2_O [32] and Na_6+*x*_(Al_6_Si_6_O_24_)(OH)*_x_*, respectively. It is to be noted that the term “anhydrous sodalite” applied to the dodalite-type compound Na_6_[AlSiO_4_]_6_ in the cited work is also misleading because sodalite *s.s*., Na_8_[AlSiO_4_]_6_Cl_2_, is also anhydrous. All these compounds are chemically and thermally more stable than most zeolites [33].

Investigation of the hydrosodalite system does not verify the existence of a solid solution between the two end-member series Na_8_[AISiO_4_]_6_(OH)_2_·*n*H_2_O (0 ≤ *n* ≤ 4 (“basic series”) and Na_6_[AISiO_4_]_6_·*n*H_2_O (0 ≤ *n* ≤ 8) (“non-basic series”) [34].

Despite the fact that a large number of compounds with the sodalite structure, differing in the chemical composition of the framework and extra-framework components, have now been synthesized, most of the work relates to the development of various methods for the synthesis of sodalite *s.s*. and, especially, basic sodalite. Depending on the synthesis conditions, which include the synthesis method (hydrothermal, solid-state, gel to melt flow, crystal transition, etc.), composition of starting materials (including templates) and temperature, sodalite and basic sodalite products having different porosity, crystallinity, particle size, and morphological features have been obtained.

In most works, hydrosodalite is synthesized by hydrothermal method, at temperatures from 80 °C to 300 °C. Most often, to obtain hydrosodalite, reaction mixtures based on kaolinite (or more reactive metakaolinite, obtained by calcining kaolinite at temperatures of 600–700 °C) and NaOH are used [35,36,37,38,39,40,41,42,43,44].

Hydrothermal synthesis of sodalite-based sorbents to be used for water purification and biodiesel production was also successfully performed in numerous works using NaOH and coal fly ash [45,46,47,48,49,50] as the starting materials. In particular, it was shown [50] that basic sodalite was formed at the expense of intermediate zeolite Na-P1 at 140 °C.

The other natural and industrial raw materials successively used for the hydrothermal synthesis of sodalite-based materials are diatomite (in combination with AlCl_3_·6H_2_O) [51], sepiolite (after preliminary fusion with NaOH or KOH at 650 °C) [52], tourmaline (in combination with NaOH) [53], basalt rock (in combination with NaOH) [54], palygorskite clay (in combination with NaOH) [55], electric arc furnace slag (in combination with NaOH) [56], windshield waste (in combination with NaOH) [57], rice husk ash (as silica source, in combination with NaOH) [58], and Al_2_O_3_ pillared montmorillonite (in combination with NaOH) [59].

Different methods to synthesize sodalite, as well as structurally related Zeolite A and faujasite, under very mild hydrothermal conditions (at 40 °C), using calcined HCl-treated clays (Ca-montmorillonite, Na-montmorillonite, illite, and chlorite) as raw materials, with the addition of Al(OH)_3_ to the hydrothermal solution are described in the review [60] containing references to 65 sources. The synthesis was performed by means of hydrothermal methods (conventional or prefusion ones). It was shown that alkaline pre-fusion improves raw material reactivity.

Hydrothermal syntheses using cristobalite, corundum, and mullite as starting substances show crystallization of basic cancrinite accompanied by its gradual transformation into hydrosodalite [61].

Homogeneous aluminate and silicate solutions prepared from sodium aluminate (Al/NaOH = 0.75), fumed silica, and sodium hydroxide were used to prepare a starting mixture for the synthesis basic sodalite [62]. The reaction was carried out at 90 °C and the product was obtained as nanosized crystals with a high surface area, unlike microsized sodalite crystals.

The decomposition of NaCaHSiO_4_ in sodium aluminate solution at 50–110 °C results in the formation of basic sodalite and katoite [63].

Basic sodalite was synthesized hydrothermally from sodium aluminate and sodium metasilicate at 90 °C for 10 h in the presence of cetyltrimethylammonium bromide (CTAB) as a template [64]. Isometric crystals and flower-like basic sodalite particles were obtained at CTAB concentrations of 0–0.5 and 1–5 mM, respectively. With an increase in CTAB concentration, HS phase increased significantly.

Hydrothermal synthesis of mesoporous sodalite-related materials was performed at 150° using various organic templates as structure-directing molecules from a sol containing oxide ratios of 1.7SiO_2_:15Na_2_O:1Al_2_O_3_:80H_2_O:0.3T, where T are templates [65]. Cetyltrimethylammonium bromide (CTABr), tetrapropyl ammonium hydroxide (TPA), organosilane, and a mixture of TPA:CTABr with mole ratio of 1:1 were used as templates. The materials obtained by this method maintain their active sites and are suitable for catalyst applications.

Mesoporous sodalite with the surface area reaching 295 m^2^/g was also prepared hydrothermally from NaAlO_2_, Na_2_SiO_3_·9H_2_O, and chlorides in the presence of NaOH using a bridged polysilsesquioxane monomer as the mesoporogen [66].

Crystallization of basic sodalite was studied in an ethanol–Na_2_O–Al_2_O_3_–SiO_2_–H_2_O system at 90 °C [67]. Sizes and morphological features of the product significantly depend on the ethanol concentration and time of the reaction. Micron-sized sodalite particles with disc- and thread-ball-like shapes were produced at low ethanol contents, whereas sodalite particles with core-shell nanostructures were dominant at high ethanol contents.

Basic sodalite particles of several micrometer sizes and different morphologies (rod-like, flower-like, and nest-like grains, microspheres, and stone-like agglomerated particles) were synthesized from sodium hydroxide, aluminum metal foil, sodium metasilicate nonahydrate, and Na_2_SiO_3_·9H_2_O, with the molar composition of 50Na_2_O:Al_2_O_3_:5SiO_2_:1000H_2_O, by water-in-oil emulsion technique in the presence of Span 80, Span 20, and Tween 80 non-ionic surfactants, at 90 °C [68].

Reverse sodalite-to-cancrinite phase transformation can be realized in Bayer liquor obtained by digestion of bauxite ores in concentrated NaOH solutions at elevated temperatures [69]. The scheme of phase transformations is:

[Aluminosilicate species] → Amorphous phase → Zeolite (Linde A) → Basic sodalite → Basic cancrinite.

In the absence of Bayer liquor, no transformation of sodalite to cancrinite takes place. The transformation is promoted by CaO, CaCO_3_ (calcite), and Ca_4_Al_2_(OH)_12_(Cl,CO_3_,OH)_2_ 4H_2_O (hydrocalumite), which are common components of the Bayer liquor [70].

Basic sodalite was synthesized at 400 °C by the gel to melt flow method using pellets prepared from Zeolite 13X, Na_86_(Si_106_Al_86_O_384_)(H_2_O)_264_, and NaOH [71]. The mechanism of this process consists of alkaline aluminosilicate gel formation and crystallization in the early period but shifts into crystal growth in NaOH melt at elevated temperature. Addition of NaCl (in the NaCl:NaOH ratio of 1:1) to the reaction mixture results in the formation of sodalite as the main product of the reaction carried out at the same conditions. No water was added to the charges. Strong alkaline fluid required for the reaction in both cases is formed as a result of NaOH solvation in water evolved from zeolite 13X.

Diatomite and organosilane (TPOAC), dimethyloctadecyl [3-(trimethoxysilyl)propyl]ammonium chloride (as the sources of silica, mainly in the crystabolite form), and NaOH (as the source of sodium and hydrogen) were also used as starting materials for solvent-free synthesis of basic sodalite [72,73].

Solid-state synthesis of sodalite *s.s*. can be realized at temperatures of 600–850 °C from mixtures of kaolinite and NaCl. Kinetics of this reaction have been studied [74,75]

Sodalite was also synthesized from a mixture of kaolinite, NaCl, and NaOH in the steam and air atmosphere by crystal transition method [76]. At 200–300 °C, sodalite is the only product of this reaction. At 400–800 °C, nepheline formed as an intermediate product. However, at temperatures above 600 °C, a high yield of sodalite can be achieved.

Sodalite single crystals up to 1 mm across were synthesized from Cl-bearing sodium aluminosilicate gel at 600–700 °C and 100–150 MPa [77]. Powder and single-crystal X-ray diffraction data show good quality of the single crystals grown.

Synthesis of sodalite-type materials with Si/Al ratios from 1.0 to 2.5 from a gel prepared from metakaoline, using tetrapropylammonium hydroxide as the organic template with subsequent calcination at 550 °C for 6 h, was reported [78] However, IR spectra of the products obtained are dominated by strong bands of an organic compound.

Zeolite NaA, Na_12_[Al_12_Si_12_O_48_], and its analogues with Na partly exchanged with K or Cs were used as starting materials (in combination with NaCl, KCl, or LiCl) to synthesize sodalite and related compounds by a solid-state method, at 800 and 900 °C for 2 to 24 h [79]. K- and Cs-exchanged zeolite A transformed to sodalite more rapidly than NaA. Sodalite could not be synthesized when the NaA was treated with KCl. The products were characterized by powder X-ray diffraction. It was shown that the reaction proceeds via formation of an intermediate carnegieite phase. Direct phase transformation from zeolite A to basic sodalite was observed during crystal growth of zeolite A in hydrothermal conditions, at 90 °C, in the presence of the biopolymer chitosan [80].

### 2.2. Synthesis of Other Sodalite-Related Materials

To date, numerous chemically different compounds with the SOD-type frameworks have been synthesized. Some of them have been listed in the reviews [22,81]. Appendix A contains significantly supplemented data on the chemical composition of sodalite-type compounds, as well as short data on their synthesis conditions.

Most sodalite-type compounds were synthesized under hydrothermal conditions, in the temperature range of 60–230 °C (rarely, at higher temperatures). Some of them (mainly, those with silicate, aluminosilicate, and aluminate frameworks) were obtained in solid-state reactions carried out at 650–1300 °C using preliminary prepared gels or stoichiometric mixtures of reactants. This fact shows a high thermal stability of the (Si,Al)_12_O_24_ frameworks. NaAlO_2_, Na_2_SiO_3_·9H_2_O, zeolite A, and kaolin are most frequently used as sources of Si and Al in the synthesis of sodalite-related materials with aluminosilicate frameworks. Organic solvents or melts of salts were also used as media for the synthesis of sodalite-type compounds.

In most sodalite-related materials obtained under hydrothermal conditions, Na^+^ is the main extra-framework cation. As a rule, their synthesis was carried out in the presence of NaOH, which is required for sufficiently high pH values of the hydrothermal solution.

Metal–organic sodalite-related compounds constitute a specific group of microporous materials of this type. Strictly speaking, they are not isostructural with sodalite s.s., but their frameworks are topologically identical to that of sodalite. Usually, these materials are synthesized in organic solutions at room temperature.

## 3. Crystallography, Crystal Chemistry, and Intra-Cage Chemical Conversions of Sodalite-Type Compounds

### 3.1. SOD-Type Topology of Zeolites and Related Materials

Natural and synthetic compounds with the SOD-type topology of the framework represent the most comprehensive family of zeolites. About ~5000 crystal structures of zeolites were deposited in the databank [82], where more than 900 belong to this topological type [83].

The ideal SOD-type framework has a cubic symmetry and contains zeolite channels consisting of sodalite cages (*sod*-cages, {0[4^6^.6^8^]}, also denoted as β-cages). This specific feature distinguishes sodalite-type materials from trigonal cancrinite-related compounds (i.e., compounds with stacking sequences of six-membered rings other than ABC) in which channels and columns of cages are running only in one direction. The unit-cell parameters of the “idealized SOD-type framework” deposited in the Database of Zeolite Structures are *a* = 8.9561 Å, *V* = 718.4 Å^3^; space group *Im*$\bar{3}$*m* (aristotype) [83,84]. However, real symmetry of compounds with the SOD-type frameworks is lower as a result of different schemes of ordering of framework atoms and extra-framework components. The group–subgroup relationship for symmetry derivatives and corresponding unit-cell settings are discussed in [83].

**Figure 2:**
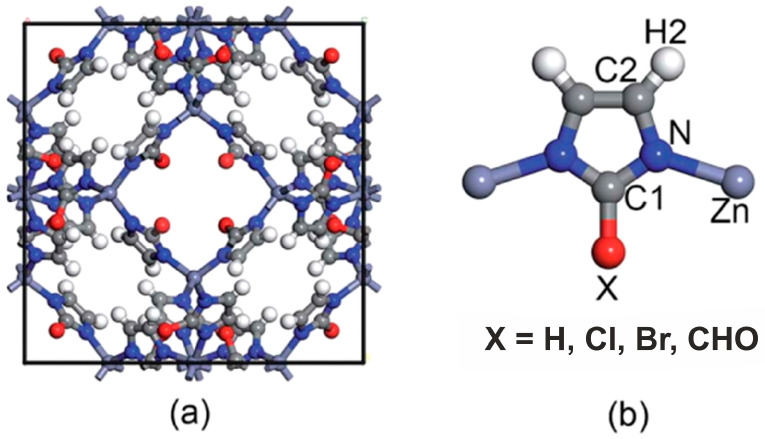
Representation of (**a**) the unit cell of ZIFs with an SOD topology and (**b**) the basic unit of ZIFs with the different functional groups [88].

**Figure 3:**
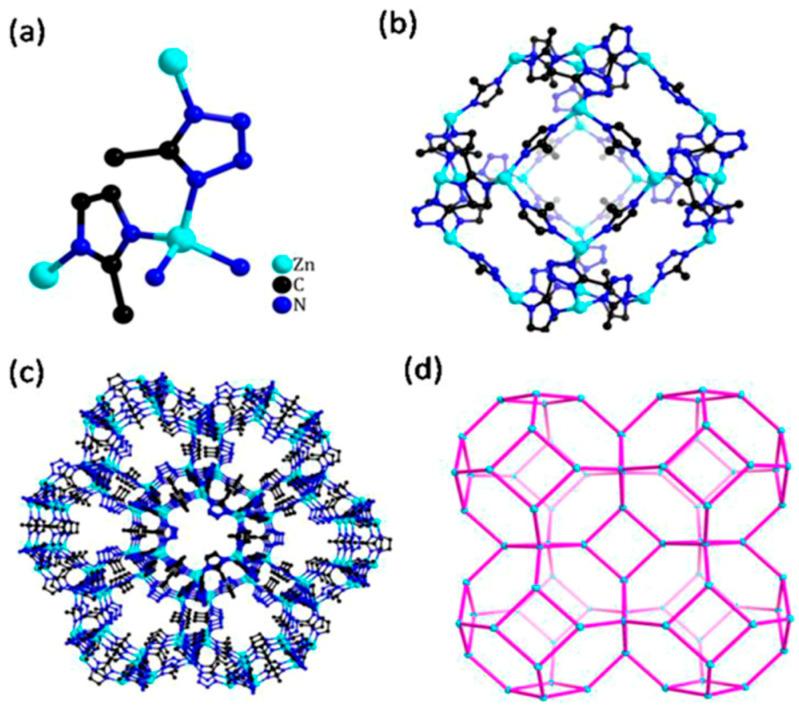
(a) Coordination mode of zinc atoms of ZTIF-8; (**b**) SOD cage constructed by Zn–tetrazolate–imidazolate; (**c**) view of the 3D framework of ZTIF-8 along the (111) direction; (**d**) topology of ZTIF-8. Reprinted with permission from [92]. Copyright 2020 American Chemical Society.

It was shown that sodalite-related crystalline solids can be divided into three kinds of compounds, namely those with the sodalite framework of all-corner connected *T*O_4_ tetrahedra and with a virtual lattice of cage cations or anions [81]. However, the SOD-type topology is also widespread among the metal–organic frameworks (MOFs), porous crystalline materials containing both organic and inorganic moieties which form a regular three-dimensional lattice [85]. Among MOFs, the zeolitic imidazolate frameworks (ZIFs) are the most frequently investigated due to their exceptionally high chemical and thermal stability with a high hydrophobic character [86]. Various ZIFs, i.e., ZIF-8, ZIF-7, ZIF-90, Zn(dcim)2-SALE, ZIF-67, and CdIF-1, are characterized by the SOD-type topology (Figure 2) [87,88,89,90,91]. The SOD-type topology also preserved in porous zeolitic tetrazolate–imidazolate frameworks (Figure 3), which have been synthesized by the introducing tetrazole ligands into ZIFs [92].

**Figure 4:**
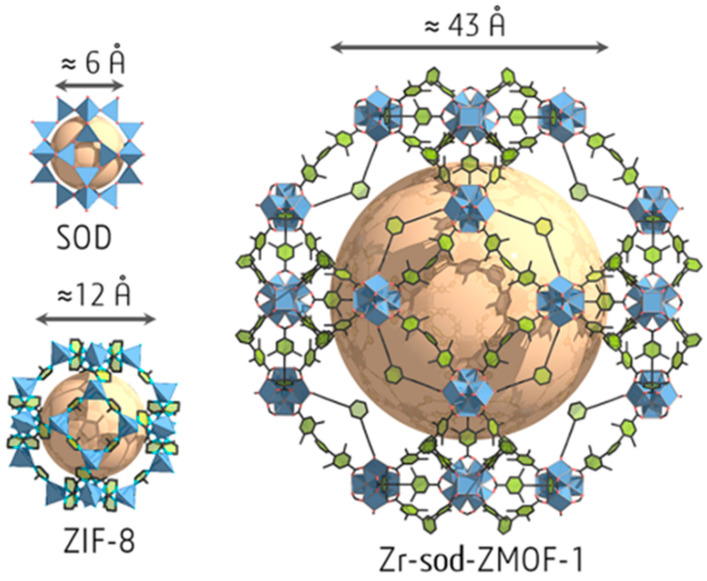
Cage size for SOD structure, ZIF-8, and Zr-sod-ZMOF-1. Reprinted (adapted) with permission from [95]. Copyright 2020 American Chemical Society.

The In^3+^ ion shows a high affinity to the carboxylate groups to form {In(O_2_C)_4_} complex with tetrahedral coordination [93]. The metal–organic SOD-type compound, (Et_2_NH_2_)[In^3+^ 5-(bis(4-carboxybenzyl)amino)-isophthalate]·4DEF·4EtOH was synthesized by a 4 + 4 synthetic strategy from tetrahedral organic building units and In^3+^ ions [94]. The mesoporous zirconium-based MOF (Zr-sod-ZMOF-1) with SOD-type topology contains mesopores with a diameter up to ~43 Å and a pore volume of 1.98 cm^3^·g^−1^ (the highest reported experimental value for zeolite-like MOFs (Figure 4) [95].

**Figure 5:**
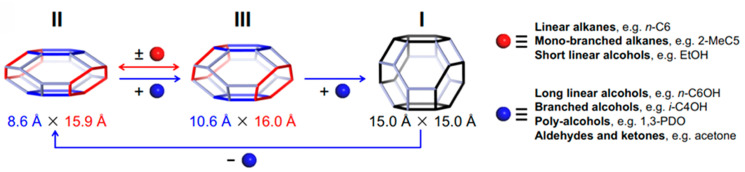
The breathing behavior and expansion magnitude of ZIF-65(Zn) is selective and responsive depending on the nature of the guest molecules [97].

Unlike typical zeolites, microporous materials with the metal–organic frameworks (MOFs) are characterized by a high framework flexibility [96]. In particular, ZIF-65(Zn) exhibits a stepwise II (contraction phase) → III (intermediate phase) → I (expansion phase) structural transition during which its ellipsoidal *sod* cage first expands to another ellipsoidal *sod* cage and then to a spherical *sod* cage (Figure 5). The breathing behavior of ZIF-65(Zn) varies depending on the nature of the guest molecules, their polarity and shape [97].

**Figure 6:**
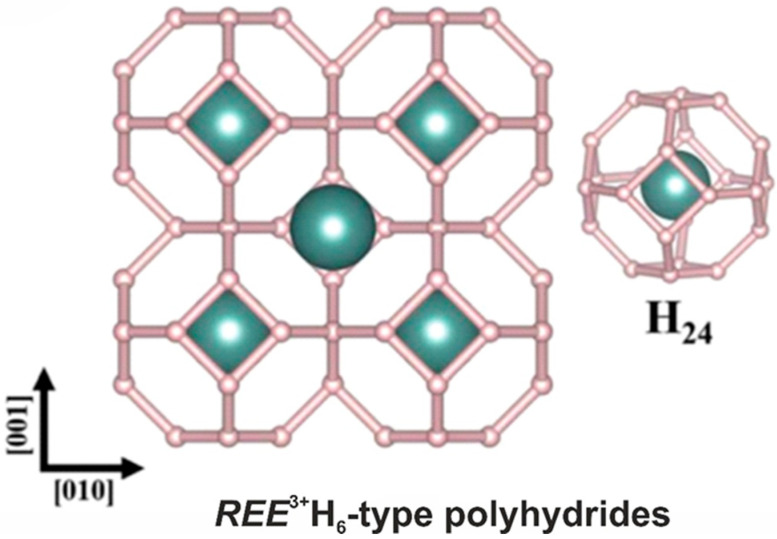
The general view of the crystal structure of a rare-earth hexahydride. The *REE* atom is shown with gray-blue.

Simple hydrides (Figure 6) attract interest as potential superconducting materials [98,99,100,101]. Among them, the SOD-type topology has been observed for the hexahydrides of rare-earth elements [102,103,104] as well as hafnium [105] and calcium [106]. Moreover, the SOD-type topology is present in the crystal structures of type VII clathrates [107], in particular, in the novel compounds SrB_3_C_3_ [108] and Si_2_Ge [109].

### 3.2. Framework Composition, Elasticity and Porosity

Sodalite-group minerals and related synthetic materials with host structures belonging to the same topological type are characterized by a wide chemical diversity and variable symmetry due to different kinds of framework distortions. These compounds are characterized by different specific properties, which make it possible to consider them as advanced materials with technologically important properties.

**Figure 7:**
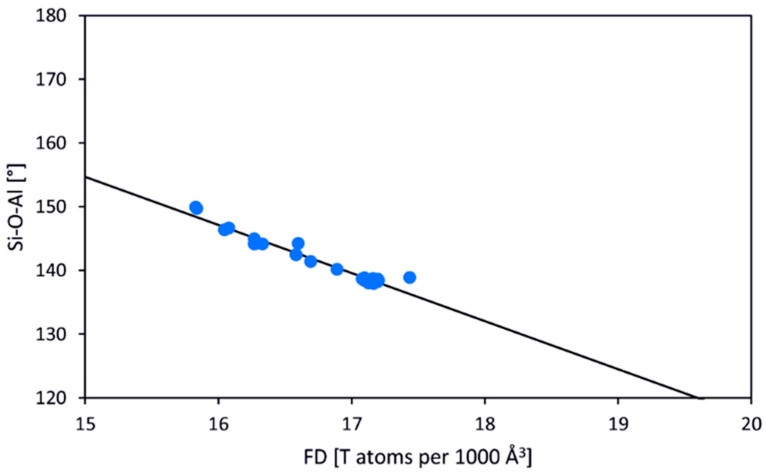
Correlation between Si–O–Al angles and framework densities (FD) for 30 individual aluminosilicate frameworks of SOD-type compounds crystallizing in space group *P*-43*n* (Pearson’s *R*^2^ is 0.95) [111].

It is obvious that geometrical characteristics including mean *T*–O bond lengths, *T*–O–*T* angles, and rotation angles of the *T*-centered tetrahedra with respect to the four-fold axis, as well as elastic and compressional properties of the SOD-type host structures, depend on the chemical composition of the framework [110]. The general features of the SOD-type frameworks have been recently reported [111] (Table 1). The framework density (the number of *T* atoms per 1000 Å^3^ [84], FD) depends crucially on the value of the mean *T*–O–*T* angle. For the most representative [Al–Si–O]-SOD type of the framework, the range of the Si–O–Al angle varies from 128° to 150°. The relationship between the *T*–O–*T* angle and FD is close to linear with the correlation coefficient *R*^2^ of 0.95 (Figure 7) [111].

**Figure 8:**
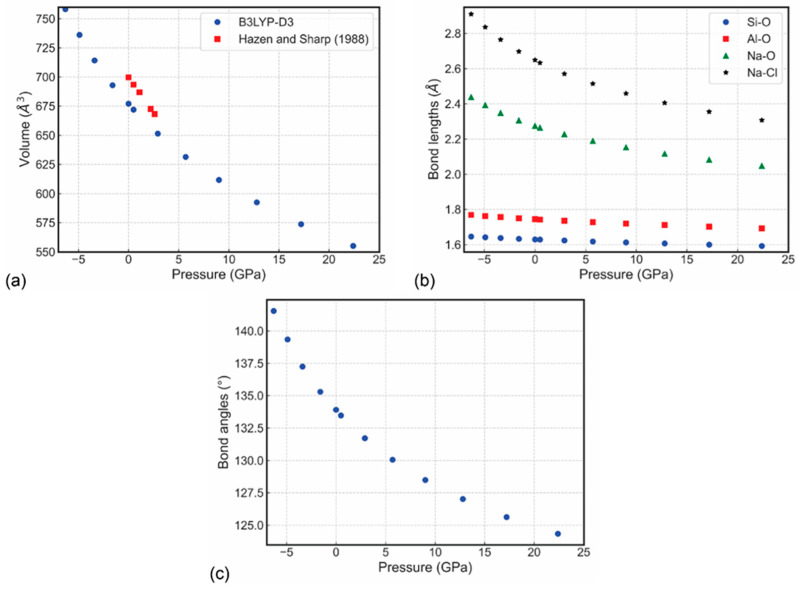
Sodalite (**a**) unit-cell volume, (**b**) bond lengths, and (**c**) Si–O–Al bridging angle variations as a function of pressure [113]. In panel (**a**), the results of Hazen and Sharp [81] are added for a direct comparison.

**Figure 9:**
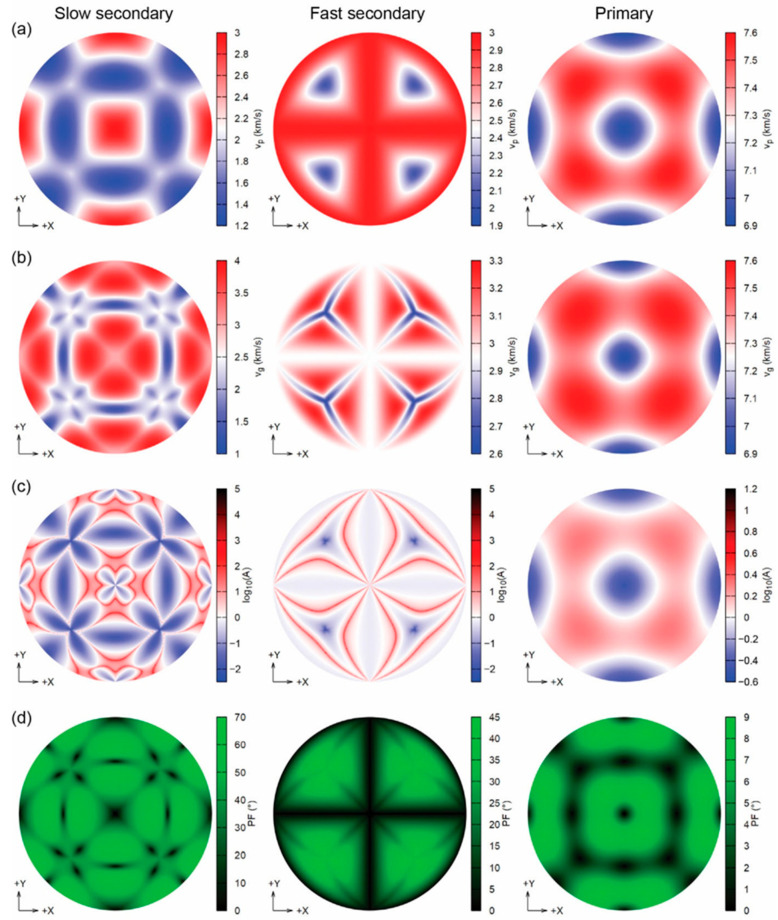
Diagrams of the seismic wave velocities (Lambert equal-area upper hemisphere projections) of sodalite at 12.8 GPa, showing (**a**) the phase velocities *v*_P_ (km/s), (**b**) the group velocities *v*_P_ (km/s), (**c**) the enhancement factor *A*, and (**d**) the power flow angle (PF, °) [113].

The [Al–Si–O]-SOD type framework is relatively soft and can be easily deformed by bending the Si–O–Al angle involving an oxygen atom shared by the SiO_4_ and AlO_4_ tetrahedra. As a result, the determined bulk elastic modulus (*K_S_* = 55.3 GPa) and the shear modulus (μ = 31.3 GPa) for sodalite, Na_8_(Al_6_Si_6_O_24_)Cl_2_, are low and comparable with those of zeolites, but are somewhat smaller than those of minerals and compounds with denser aluminosilicate frameworks such as monoclinic K-feldspar (*K_S_* = 67.3 GPa; μ = 35.2 GPa) [112]. The recent data obtained from DFT simulations reported the calculated equation of state parameters at 0 GPa and absolute zero (0 K) [113] are: elastic modulus *K*_0_ = 70.15(7) GPa, its pressure derivative *K′* = 4.46(2), and unit-cell volume *V*_0_ = 676.85(3) Å^3^ (Figure 8), which are in agreement with the experimental data [49,114]. The seismic velocity results, i.e., phase velocity *v_p_*, group velocity *v_g_*, enhancement factor *A*, and power flow angle *ψ* for sodalite at 12.8 GPa are presented in Figure 9, as the upper hemisphere (Z > 0) of Lambert equal-area projections on the XY plane.

**Figure 10:**
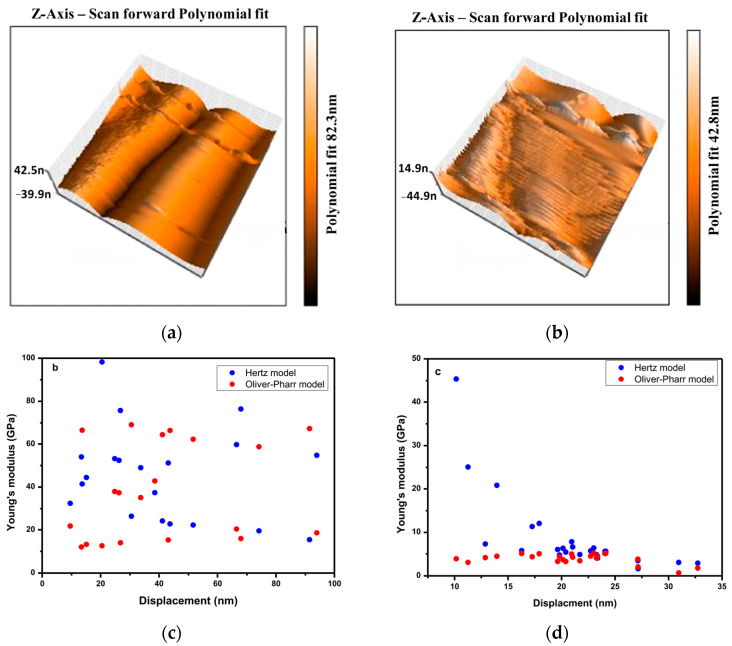
The 3D AFM topography of sodalite (**a**) and PCL/sodalite nanocomposite (**b**), and the corresponding relationship between elastic modulus and displacement for sodalite (**c**) and PCL/sodalite nanocomposite (**d**) [115]. The images were acquired with sizes of 2.56 μm × 2.56 μm.

The mechanical properties of large sodalite single crystals were measured and the values of hardness and elastic modulus are equal to 6 GPa and 40 GPa, respectively [115]. The Young’s modulus of sodalite determined using atomic force microscopy with two contact models (Hertz and Oliver–Pharr) varies between 69.02 and 12.01 GPa for the Hertz and the Oliver–Pharr models, respectively (Figure 10b) [115]. These modulus for nanocomposite, containing sodalite and polycaprolactone (PCL) as reinforcement, range from 1.75 to 6.66 GPa and 1.63 to 45.36 GPa for the cited models, respectively. The deviation of the Young’s modulus can be explained by the presence of defects on the surface contact area (Figure 10a).

The compressibility of the orthorhombic sodalite-type compound Ca_8_(Al_12_O_24_)(SO_4_) was studied [116]. This material is more compressible than other Al-bearing cement phases.

Cubic silica sodalite with 1,3,5-trioxane template, [Si_12_O_24_]·2 C_3_H_6_O_3_ (*a*_0_ = 8.8349 Å), was studied at room temperature and at elevated pressures up to 1.28 GPa with neutron powder diffraction [117]. The crystal structure refinement was carried out by means of the Rietveld method. It was shown that cubic symmetry retained in the whole pressure range and the linear bulk modulus is equal to 132 GPa. The volume reduction under pressure is mainly a result of cooperative tilting of the system of framework SiO_4_ tetrahedra.

### 3.3. Inorganic and Organic Guest Species in the Sodalite Cages

In sodalite-type compounds containing relatively small extra-framework uni- or bivalent metal M-cations (M = Li^+^, Na^+^, Mn^2+^, Fe^2+^, Mn^2+^, Zn^2+^, Cd^2+^, Mn^2+^, etc.) and relatively small monoatomic X-anions like Cl^−^, Br^−^, I^−^, S^2−^, Se^2−^, or Te^2−^, these components form anion-centered tetrahedral [M_4_X] clusters occurring in the sodalite cages In particular, the clusters Mn_4_S^6+^ (with the effective radii (*r*) of “guest” tetrahedron, *r* = 3.44 Å), Cd_4_S^6+^ (*r* = 3.78 Å), Na_4_Cl^3+^ (*r* = 3.75 Å), Na_4_Br^3+^ (*r* = 3.90 Å), Na_4_I^3+^ (*r* = 4.14 Å), Zn_4_S^6+^ (*r* = 3.32 Å), Zn_4_Se^6+^(*r* = 3.39 Å), Fe_4_S^6+^ (*r* = 3.32 Å), Fe_4_Se^6+^ (*r* = 3.39 Å), Fe_4_Te^6+^ (*r* = 3.59 Å), and Li_4_Cl^3+^ (*r* = 3.17 Å) have been identified [118,119,120,121,122]. Similar Na_4_(HS)^3+^ clusters formed by the HS^−^ and Na^+^ extra-framework ions were found in the natural HS^−^ sodalite analogue, sapozhnikovite, Na_8_(Al_6_Si_6_O_24_)(HS)_2_ [17]. Sapozhnikovite and sodalite, Na_8_(Al_6_Si_6_O_24_)Cl_2_, form a complete solid–solution series [24].

Tetrahedral (H_2_O)_4_Na clusters with the Na···O distance of 2.6 Å occur in the structure of nosean, Na_8_(Al_6_Si_6_O_24_)(SO_4_)·H_2_O [8]. Partial substitution of Na^+^ with hydronium in this cluster results in the formation of the Zundel cation, H_5_O_2_^+^ [26].

Intermediate members of the solid–solution system Ga*_x_*Zn_8−_*x*P*_x_*Se_2−*x*_(BO_2_)_12_ having a sodalite-type structure were synthesized by solid-state reactions of stoichiometric mixtures of ZnB_4_O_7_, ZnO, GaP, and ZnSe at 900–950 °C [121]. Inclusion of GaP within the borate sodalite analogue results in the formation of an isolated ^31^P,^69,71^Ga spin pair that exhibits resolved scalar coupling in the ^31^P MAS NMR spectra. It was shown that P and Se have tetrahedral coordination and occur in four kinds of domains, [Zn_4_Se]^6+^, [GaZn_3_P]^6+^, [Zn_4_P]^5+^, and [Ga_2_Zn_2_P]^7+^.

In_4_O_4_^4+^ clusters can be also incorporated into sodalite cages of zeolite Y [123].

The sodalite-type compound Na_6_Li_1.6_K_0.4_(Al_6_Si_6_O_24_)Cl_2_ is similar to sodalite proper, but the introduction of Li into the sodalite cages instead of Na results in a collapse of the framework. Refinement of an X-ray powder diffraction pattern yielded for this compound a lattice parameter of 8.8427 Å, an Al–O–Si bond angle of 137.9°, and Al–O and Si–O bond lengths of 1.730 and 1.620 Å, respectively [124].

Unit-cell parameters (*a*, Å) of two series of cubic sodalites with mixed cation and anion sites, *M*^+^_8_(AlSiO_4_)_6_Cl_2_ (*M* = Li, Na, K, Rb, and/or Ag) and Na_8_(AlSiO_4_)_6_*X*^−^_2_ (*X* = Cl, Br, and/or I), have been related to the mean radii (〈*r*〉, Å) of the *M*^+^ and *X*^−^ ions [125]. The following correlations have been obtained:

*a* = 0.3974 〈*r_X_*〉 + 8.1713 (*R*^2^ = 0.9731) and *a* = 1.061〈*r_M_*〉 + 7.852 (*R*^2^ = 0.9909).

The radii of the cations and anions with the coordination number of IV used to obtain these correlations are (in Å): Li^+^ 0.60, Na^+^ 0.95, K^+^ 1.33, Rb^+^ 1.48, Ag^+^ 1.00, Cl^−^ 1.78, Br^−^ 1.93 and I^−^ 2.155 [126,127].

A similar trend was observed for germanate sodalites: the cubic unit-cell *a* parameters of Na_8_(Al_6_Ge_6_O_24_)*X*_2_ with *X* = Cl, Br, and I are, respectively, 9.0641, 9.1049, and 9.1874 Å [128].

Unit-cell parameters of synthetic aluminosilicate sodalites with different extra-framework anionic groups are given in Table 2.

Complete solid solution exists between synthetic products of nosean and haüyne composition, but only limited solid solution occurs in synthetic products of sodalite–nosean and sodalite–haüyne compositions formed at 600 °C [133]. However, wide variations of the Cl:S ratio were found in sodalite-group minerals from the Lovozero alkaline complex. It was shown that these minerals do not contain sulfate groups and belong to the sodalite–sapozhnikovite solid–solution series, Na_8_(Al_6_Si_6_O_24_)(Cl,HS) [24].

In addition to cations, anions, and radical anions, unpaired electrons and neutral clusters like Pb_4_O_4_, as well as organic molecules, are known as guest components in SOD-type materials [81].

### 3.4. Symmetry, Structure Modulations, and Twinning

The idealized (aristotype) sodalite framework deposited in the Database of Zeolite Structures is cubic, space group *Im*$\bar{3}$*m*, with *a* = 8.9561 Å and *V* = 718.4 Å^3^ [84]. However, in most compounds topologically identical with sodalite, various distortions of this ideal structure are observed. In particular, different schemes of the Si/Al ordering as well as specific features of the occupation of the sodalite cages may result in the symmetry lowering, structure modulations, twinning, etc.

Structure modulations of sulfate sodalite-group minerals are very typical and have been described in numerous publications [7,134,135,136,137,138,139]. In particular, structure modulations of different kinds are a specific feature of minerals belonging to the haüyne–lazurite solid–solution series [140,141,142,143,144,145,146,147,148,149].

In some samples, both commensurate and incommensurate modulations are observed (Table 3).

Symmetry relationships of sodalite-type crystal structures were considered in detail in [83]. Usually, cubic symmetry remains, but real symmetry of some sodalite-type minerals and compounds can be orthorhombic [13,14,150,151,152], monoclinic [28,153], and even triclinic [18]. Often, symmetry lowering is accompanied by commensurate and incommensurate structure modulations. Powder X-ray diffraction patterns of such samples contain both basic and superstructural reflections [7]. Basic reflections correspond to cubic pseudo-cells. The displacement of the satellite from the main reflection along the reciprocal lattice axis determines the modulation parameter *n*. In X-ray diffraction patterns of structurally modulated sodalite-related compounds, integer *hkl* indices of satellites correspond to commensurate modulations, whereas incommensurate modulations cannot be described by integer *hkl* indices. Thus, commensurate modulations can be treated as multiplications of the basic *a* parameter of the sodalite unit cell.

Commensurate modulations were observed for aluminate sodalite [154].

**Figure 11:**
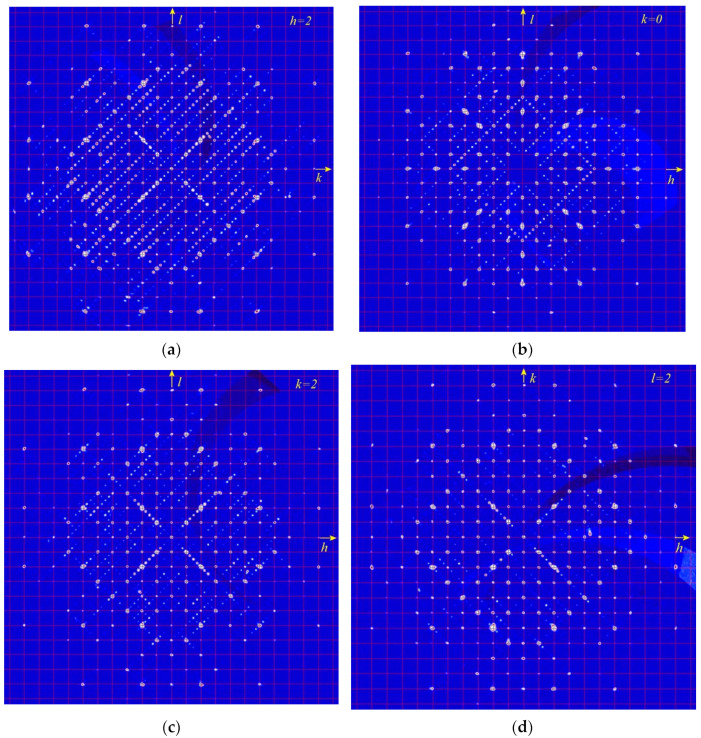
The sections of the diffraction pattern of modulated monoclinic LRM with **q**~0.43**c** in the reciprocal space by the planes *h* = 2, *k* = 0, *k* = 2 and *l* = 2 (the pictures (**a**), (**b**), (**c**) and (**d**), respectively).

A comparative structural analyses of modulated cubic, orthorhombic, monoclinic, and triclinic lazurite-related minerals (LRM) was carried out in [28]. The results are given in Table 4 and examples of sections of the diffraction pattern obtained for modulated monoclinic LRM with **q**~0.43**c** showing superstructure reflections is given in Figure 11. It was concluded that the character of modulations depends on the kind of ordered or disordered alternation of SO_4_^2−^ anions and polysulfide species (S_3_^•−^, S_4_, S_6_).

Cubic LRM show different schemes of structure modulations. In the diffraction pattern of the cubic RLM-2 (Table 4), the average structure of which was studied in [143], strong satellites with fractional indices *h* ± δ, *k* ± δ, *l*), (*h* ± δ, *k*, *l* ± δ), and (*h*, *k* ± δ, *l* ± δ), δ ≈ 0.2154(1) ≈ 3/14 were observed in six directions [144]. Satellites with indices (*h* ± 2δ, *k* ± δ, *l* ± δ) may by a superposition of two or three differently directed waves. The latter served as an argument for modeling this structure in the (3 + 3)D space. The construction of the (3 + 3)D model for the cubic LRM involved three vectors of equal lengths: **q**_1_ = δ(**a**_cub_* + **b**_cub_*), **q**_2_ = δ(**a**_cub_* + **c**_cub_*), **q**_3_ = δ(**b**_cub_* + **c**_cub_*); δ = 0.2154(1) [146]. In the corresponding orthorhombic setting of the coordinate axes, any of these vectors can be represented as **q** ≈ 0.43c*.

Modulation of the crystal structure of LRM-1 (Table 4) occurs in the same main directions with wave vectors of shorter lengths: **q**_1_ = δ(**a**_cub_* + **b**_cub_*), **q**_2_ = δ(**a**_cub_* + **c**_cub_*), **q**_3_ = δ(**b**_cub_* + **c**_cub_*); δ = 0.1479 or **q** ≈ 0.30c* in the orthorhombic setting.

Annealing of cubic S_3_^•−^-bearing haüyne at 550 °C results in complex transformations of its framework, including changes of modulation patterns [155]. During the first three days of the experiment, coexistence of satellite reflections with the incommensurability parameters δ of 0.217 and 0.147 was observed. The intensities of the former satellite reflections gradually decreased, whereas the intensities of the latter satellites increased and, after reaching maximum values, started to decrease and finally disappeared. The resulting structure was cubic without structural modulations. Similar processes were observed for other samples of LRMs. The most probable cause of these phenomena is change of ordering of extra-framework sulfate and sulfide groups and their mutual transformations [24,26].

**Figure 12:**
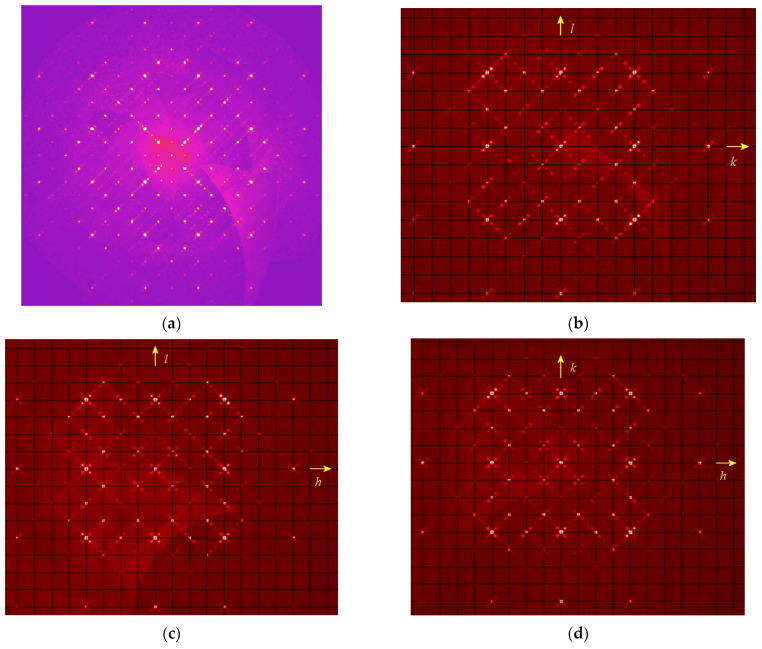
The sections of the diffraction pattern of S_4_-bearing LRM in the reciprocal space by the planes *h* = 2, *h* = 4, *k* = 4, and *l* = 4 (the pictures (**a**), (**b**), (**c**), and (**d**), respectively). In (**b**–**d**), the reciprocal lattice is shown with black net.

A specific case is cubic LRM with alternating SO_4_^2−^ and S_4_ groups which results in the incommensurate modulation. Different sections of the 3D diffraction pattern in the reciprocal space are shown in Figure 12. As one can see, the main reflections at lattice sites are surrounded by satellites oriented along the diagonals of the squares (crystallographic directions <110>) and spaced from the main reflections by about 0.222 square diagonals. Additionally weak superstructural reflections can be observed in the centers of the squares. In general, the 3D picture contains disproportionate satellites in six directions <110>.

**Figure 13:**
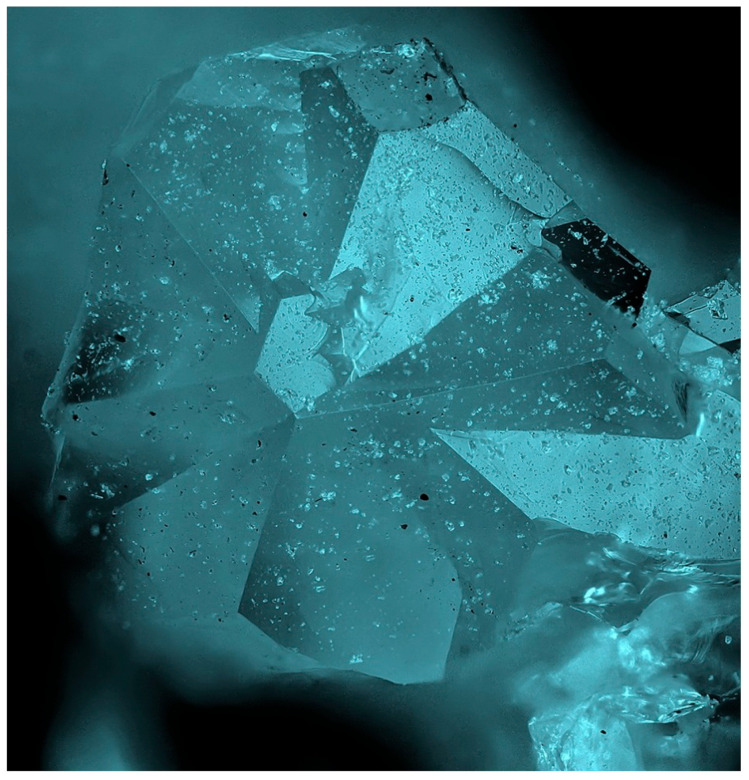
A typical sodalite penetration twin.

**Figure 14:**
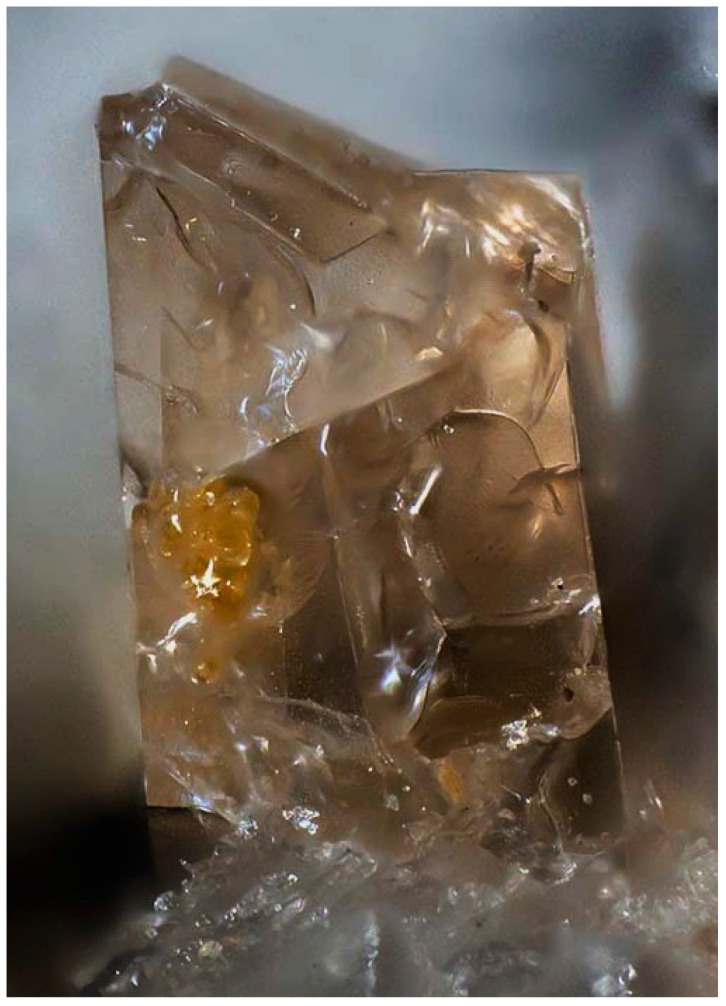
Sodalite contact twin. Photographer: V. Heck.

Twinning is one more cause of violation of translational periodicity of the crystal structure. The formation of growth twins is typical for minerals of the sodalite group and synthetic materials with the SOD-type structures. Both penetration and contact sodalite twins are known (Figure 13 and Figure 14).

**Figure 15:**
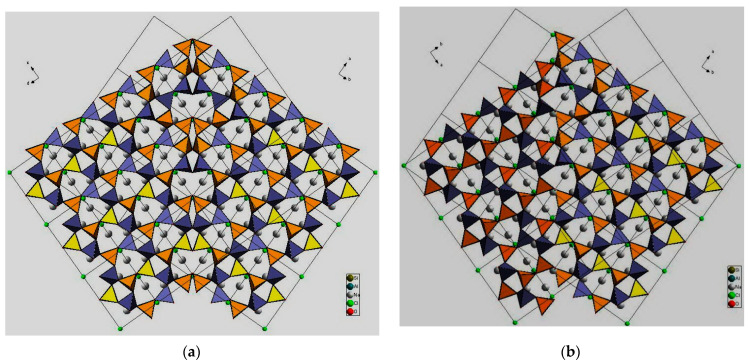
Crystal structures of (**a**) reflection and (**b**) rotation twins of sodalite.

Based on morphological features, one can suppose that components of such twins are connected either by a 180-degree turn around the [156] axis of the cubic lattice or by a reflection in any of the planes ($\bar{2}$11), (1$\bar{2}$1), or (11$\bar{2}$). Both kinds of twins are morphologically undistinguishable, with an angle of 109.5° between the (111) axes of the twin components (Figure 15).

Important properties of the SOD-type materials related to transport of small species crucially depend on the kind and concentration of 2D defects among which the boundary between the twin components plays the most significant role. Analysis of interatomic contacts at the border between twin components [157] has shown that twinning by a plane is energetically most favorable because it requires the smallest distortion and straining.

A haüyne sample from Sacrofano, Italy with the composition close to Na_4.5_Ca_2_K[Al_6_St_6_O_24_](SO_4_)_1.5_(OH)_0.5_ and unit-cell parameter of 9.116 Å was studied by single-crystal X-ray diffraction and selected-area electron diffraction [138]. Based on the structure analysis and composition, it was concluded that 75% of sodalite cages contain [Na_3_Ca·SO_4_]^3+^ clusters, the remaining 25% of the cages contain [K_2_Ca·OH]^3+^ clusters, and the space group *P*23 applies for each domain. Alternation of these domains results in structure modulations and diffuse streaked reflections of the single-crystal X-ray diffraction pattern. It should be noted that most available precise chemical analyses do show the occurrence of significant amounts of OH groups in haüyne: the formulae with minor H_2_O and without OH groups are charge-balanced due to deviations of the contents of extra-framework cations and Si:O ratios from the ideal values.

### 3.5. Intracage Reactions Involving H, S, C, N, B, Cl, and Mn in Sodalite-Type Compounds

Encapsulation of various species in sodalite cages significantly enhances their thermal stability. This effect can be used for the stabilization of various species including phosphors, chromophores, and sources of hydrogen.

Although pure sodium chlorate decomposes at ~300 °C, decomposition of encapsulated chlorate in sodalite cage of Na_8_(Al_6_Ge_6_O_24_)(ClO_3_)_2_ takes place above 600 °C [158]. Similarly, the C_2_O_4_^2−^ and CO_3_^2−^ anions in sodalite cages remain stable up to 700 °C and 800 °C, respectively [24,26,27], whereas pure sodium oxalate starts to decompose at 290 °C [159].

Sodium thiocyanate decomposes below 400 °C [160]. However, the decomposition of thiocyanate anion in the aluminogermanate sodalite Na_8_(AlGeO_4_)_6_(SCN)_2_ starts at around 750 °C [161].

Pure sodium azide decomposes at ~350 °C, while encapsulation of sodium azide into a sodalite cage results in its stabilization: evolution of nitrogen from azide sodalite Na_8_(AlSiO_4_)_6_(N_3_)_2_, synthesized hydrothermally from zeolite A and NaN_3_, takes place in the temperature interval from 600 °C to 700 °C [162]. Below 600 °C, the compound shows positive thermal expansion. Correlations between the unit-cell parameter and Al–O bond distance with temperature has been established.

Two reactions in sodalite cages have been modeled using a phenomenological approach using cellular automata models for cage template reactions: the thermal decomposition of the MnO_4_^2−^ anion in the Na_8_(Al_6_Si_6_O_24_)(MnO_4_)_2_ sodalite and the transformation of the nitrite analogue of sodalite, Na_8_(Al_6_Si_6_O_24_)(NO_2_)_2_, to the anhydrous carbonate analogue of nosean, Na_8_(Al_6_Si_6_O_24_)(CO_3_), on heating in a CO_2_ atmosphere [160].

According to [156], the MnO_4_^−^ ion in permanganate sodalite decomposes above 600° via the reaction:

2MnO_4_^−^ → MnO_4_^2−^ + MnO_2_ + O_2_,

whereas pure KMnO_4_ starts to decompose below 250 °C [163].

Carbonatization of nitrite sodalite occurs by the mechanism:

Na_8_(Al_6_Si_6_O_24_)(NO_2_)_2_ + CO_2_ → Na_8_(Al_6_Si_6_O_24_)(CO_3_) + NO + NO_2_,

in accordance with [164].

The concentrations S_2_^•−^ and S_3_^•−^ radical anions in blue ultramarine pigment increased on heating under a dynamic vacuum up to 700 °C, as deduced from ESR and Raman spectroscopy data [165]. It was supposed that these chromophore sulfur species could form from S^2−^ occurring in initial ultramarine. However, studies of natural ultramarine did not confirm this assumption.

Application of a multimethod approach based on infrared, Raman, ESR, UV–Vis–near IR absorption spectroscopy, electron microprobe, wet chemical analyses, and powder and single-crystal X-ray diffraction data [19,20,21,22,23,24,25,26] has shown that S-bearing extra-framework components in sodalite-group minerals are very diverse and include SO_4_^2−^, SO_3_^2−^, and HS^−^ anions, S_2_^•−^, S_3_^•−^, *cis*- and *trans*-S_4_^•−^, and SO_4_^•−^ radical anions, as well as neutral molecules (*cis*-, *gauche*-, and *trans*-S_4_, cyclic S_6_, and COS). No S^2−^ anions detectable by the single-crystal X-ray analysis were found in natural ultramarine analogues.

Sodalite cages are specific microreactors in which mutual transformation of these species can be realized. Investigation of these processes extends knowledge on sulfur chemistry in general.

Preheating of S_3_^•−^-bearing haüyne at 700 °C under reducing conditions (over the Fe-FeS buffer) results in the transformation of SO_4_^2−^ groups into monosulfide S^2−^ anions, S_2_^•−^ and S_4_^•−^ radical anions, and S_4_ molecules. Raman, ESR, and UV–Vis–near IR absorption spectra of both initial haüyne samples and products of their annealing at 800 °C in air [24] show that various polysulfide radical anions are converted into S_2_^•−^ and S_4_^•−^ radical anions and S_4_ neutral molecules during heating at 700° in reducing atmosphere, whereas the S_3_^●−^ radical anion is stable during heating at 800° in air. These data are in good agreement with the experimental results obtained in [166,167], showing that S_2_^•−^ and S_3_^•−^ are the most thermally stable polysulfide radical anions.

Annealing of preheated haüyne at 800 °C in air first results in the enhancement of its unit-cell *a* parameter from 8.84 to 8.97 Å, subsequent formation of a new cubic phase with the *a* = 9.05, then enhancement of *a* up to 9.08 Å. These transformations are due to partial conversion of polysulfide species to form SO_4_^2−^.

**Figure 16:**
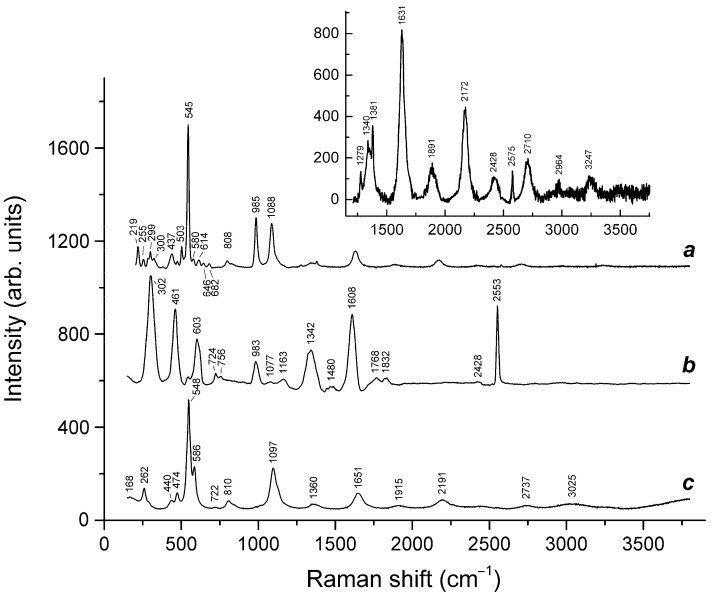
Raman spectra of (**a**) initial slyudyankaite, (**b**) slyudyankaite preheated for three days at 700 °C, over the Fe-FeS buffer, and (**c**) preheated slyudyankaite additionally annealed at 800 °C in air for one day. The inset shows the Raman spectrum of initial slyudyankaite in the range 1200–3750 cm^−1^.

Slyudyankaite is a triclinic sodalite-group mineral with the idealized formula Na_28_Ca_4_(Si_24_Al_24_O_96_)(SO_4_)_6_(S_6_)_1/3_(CO_2_)·2H_2_O and minor contents of S_4_, H_2_S, S_2_^•−^, and S_3_^•−^ [18]. In its crystal structure, SO_4_^2−^ anions (together with cations and minor S_2_^•−^ and S_3_^•−^ radical anions) and neutral species (S_6_, CO_2_, H_2_O, S_4_, and H_2_S) are completely ordered and occur in different sodalite cages. Preheating of slyudyankaite at 700 °C under reducing conditions [24] results in the transformation of S-bearing species into HS^−^, S_2_^•−^, and S_4_^•−^, and, possibly, monosulfide anion S^2−^. Simultaneously, reduction of CO_2_ occurring in initial slyudyankaite results in the formation of carbonate and acid oxalate anions (Figure 16, Table 5). Further annealing of preheated slyudyankaite at 800 °C in air results in the disappearance of HS^−^, S_2_^•−^, and S_4_^•−^, restoration of the SO_4_^2−^ and S_3_^•−^ anionic groups, and decomposition of acid oxalate groups (Figure 16, Table 5). Data from Raman spectroscopy show that S_3_^•−^ radical anions in annealed slyudyankaite occur in reduced sodalite cage in a straitened state. It is remarkable that acid oxalate groups occurring in sodalite cages are stable at 700 °C even under oxidizing conditions, whereas crystals of oxalate salts decompose at much lower temperatures.

Thus, experiments with annealing of sulfate sodalite-group minerals with cubic structures show that under high-temperature oxidizing conditions (in air, at 800 °C) S_3_^•−^ is the most stable sulfide species. Annealing of the preheated samples in air at 800 °C results in partial reverse transformations:

S_2_^•−^ + S^2−^ + 2O_2_(gas) → SO_4_^2−^, S_4_^•−^ + S_2_^•−^ → 2S_3_^•−^,

as well as subordinate processes

S_3_^•−^ + 5e +6O_2_(gas) → 3SO_4_^2−^

and

C_2_O_4_^2−^ → 2CO_2_(gas) + 2*e* (*e* = electron).

The oxidation of S_3_^•−^ into SO_4_^2−^ can proceed only partly due to the charge-balance requirement.

The partial thermal transformation of SO_4_^2−^ to S_3_^•−^ in sodalite cages is possible even under oxidizing conditions. In particular, heating natural haüyne from paleovolcanic rocks of Mt. Vulture, Italy up to 750 °C in air leads its crystals to acquire a blue color and appearance of the bands at 260, 547, 585, 810, and 1096 cm^−1^ in the Raman spectrum [168]. These bands were assigned to S_3_^•−^ (blue chromophore) and S_2_^•−^ (yellow chromophore). However, the assignment of some of these bands to S_2_^•−^ is erroneous: all of them correspond to different modes of S_3_^•−^ (see below, in Section 4.9: Vibrational Spectroscopy of Sodalite-Group Minerals) whereas the band of S_2_^•−^ stretching vibrations (in the range 602–612 cm^−1^) is absent in the Raman spectrum of heated haüyne from Mt. Vulture.

**Figure 17:**
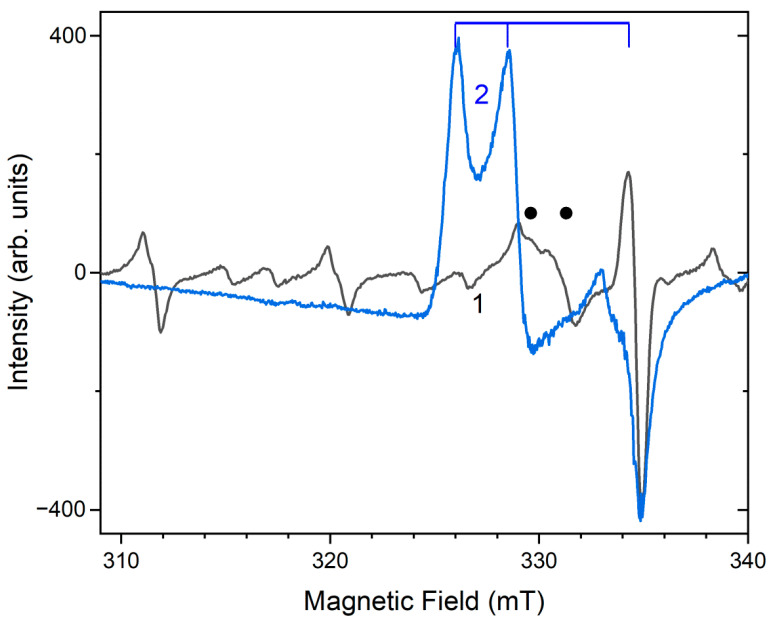
The ESR spectra of unheated S_4_- and CO_2_-bearing haüyne and product of its thermal transformation at 800 °C (2) [24]. The dots indicate the bands of S_4_^•−^ and the vertical lines show the bands of S_3_^•−^.

**Figure 18:**
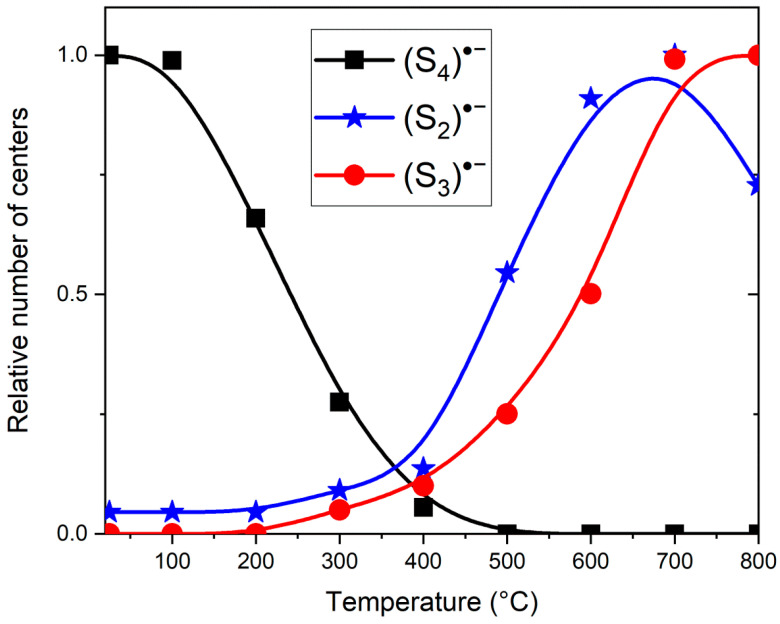
Relative amounts of various sulfur radical anions in S_4_- and CO_2_-bearing haüyne heated at different temperatures. The values of the relative amounts are normalized to the maximum number of each of the radical anions.

Similar experiments with other samples belonging to the haüyne–lazurite solid–solution series [24,26] confirmed these trends. In lilac haüyne with significant amounts of S_4_ and CO_2_ extra-framework molecules, detected in the IR and Raman spectra, no lines of S_3_^●−^ centers were observed in the Raman and ESR spectra before heating. Along with six lines associated with Mn^2+^, there is a weak signal with *g*-tensor components of 2.034 and 2.021, associated with S_4_^●−^ centers (Figure 17). ESR lines of S_3_^●−^ appear above 200 °C and their intensities reach maximum values at 700 °C (Figure 18).

**Figure 19:**
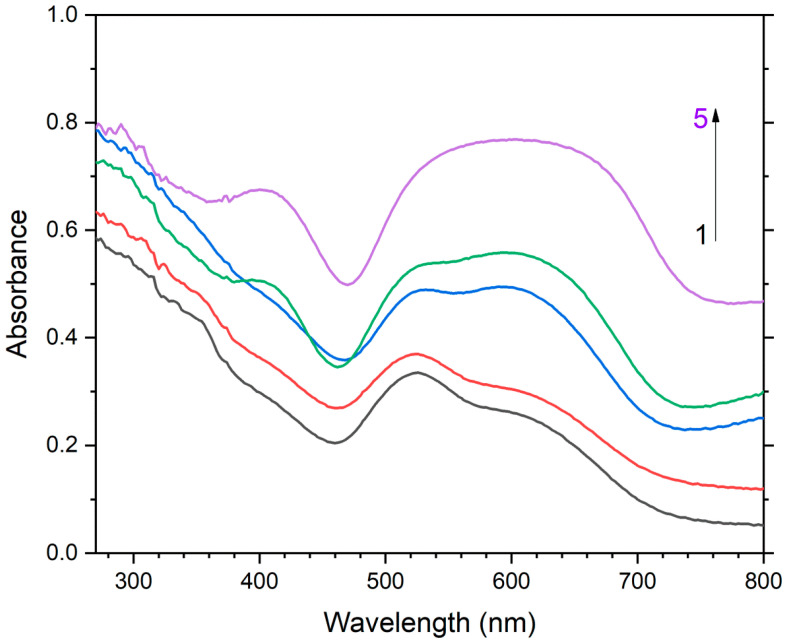
Spectra of diffuse absorption of: unheated S_4_- and CO_2_-bearing haüyne (1) and products of its thermal conversions at 200 °C (2), 400 °C (3), 600 °C (4), and 800 °C (5) [24].

Two absorption bands of S_4_- and CO_2_-bearing haüyne with maxima at 525 and 585 nm are associated with S_4_ centers. Intensities of ESR lines of S_2_^•−^ are below their detection limits. However, a weak absorption band at 400 nm corresponds to S_2_^•−^ centers (Figure 19). Being excited in the 400 nm region, the sample shows intense luminescence with a maximum in the 650 nm region associated with S_2_^•−^ radical anions.

Raman bands of the oxalate anion was detected in the sample heated at 700 °C. Thus, the main channel of transformations of extra-framework components in S_4_- and CO_2_-bearing haüyne at 700 °C under reducing conditions (in the presence of Fe-FeS buffer) is SO_4_^2−^ → S^2−^ + 2O_2_(gas), and the subordinate processes are

CO_2_ + 2SO_4_^2−^ + H_2_O → 2HS^−^ + CO_3_^2−^ + 4O_2_(gas),

3SO_4_^2−^ → S_3_^•−^ +5*e* +6O_2_(gas),

3S_4_ + 4*e* → 4S_3_^•−^, 2S_3_^•−^ → S_2_^•−^ + S_4_^•−^,

and

2CO_2_ + 2*e* → C_2_O_4_^2−^ (*e* = electron).

Annealing of the preheated samples in air at 800 °C results in partial reverse transformations:

S_2_^•−^ + S^2−^ + 2O_2_(gas) → SO_4_^2−^,

S_4_^•−^ + S_2_^•−^ → 2S_3_^•−^,

as well as subordinate processes

S_3_^•−^ + 5e +6O_2_(gas) → 3SO_4_^2−^

and

C_2_O_4_^2−^ → 2CO_2_(gas) + 2*e*.

Numerous experimental data, including those described above, show that HS^−^-bearing sodalite-group minerals crystallized under reducing conditions and CO_3_^2−^-bearing sodalite-group minerals can be formed as a result of thermal transformation of an initial CO_2_-bearing mineral. This conclusion is in agreement with the occurrence of the HS^−^-dominant sodalite-group mineral sapozhnikovite in association with the oxalate cancrinite-analogue [17] and the occurrence of a CO_3_^2−^-bearing haüyne in a thermally metamorphosed rock [169].

The extra-framework species CO_2_ and HS^−^ are indicators of strongly oxidizing and strongly reducing conditions, respectively. The simultaneous presence of these species in minerals of the sodalite group has never been observed. Under moderately oxidizing conditions CO_2_ can coexist with COS molecules in the structures of minerals belonging to the haüyne–lazurite solid–solution series [27]. Corresponding equilibrium can be described with the scheme 2CO_2_(solid) + S_3_^•−^(solid) + O_2_(gas) ↔ 2COS + SO_4_^2−^.

Based on the Raman spectroscopy data, it was shown [26] that the main channel of high-temperature transformations of the HS^−^ anion in the synthetic analogue of sapozhnikovite, Na_8_(Al_6_Si_6_O_24_)(HS)_2_, is

2HS^−^ (solid) + 2.5O_2_ (gas) → SO_4_^2−^ (solid) + 0.25S_4_ (solid) + H_2_O (gas).

Hypothetically, the subordinate channels of transformations of the HS^−^ anion during heating this sample under oxidizing and moderately reducing conditions can be described by the schemes

2HS^−^ (solid) + 3.5O_2_ (gas) → SO_4_^2−^ (solid) + SO_2_ (gas) + H_2_O (gas)

and

6HS^−^ (solid) → S_3_^•−^ (solid) + 3H_2_S (gas),

respectively.

It is remarkable that in most natural sodalite-group minerals, sulfur in sodalite cages occurs in sulfate and/or sulfide form whereas the admixture of S(IV) in the form of the SO_3_^2−^ anion was found only in one haüyne sample [27]. On the contrary, SO_3_^2−^ is a rather common component in larger liottite cages of multilayer cancrinite-group minerals [170,171,172,173].

The causes for the difficult entry of trigonal groups, CO_3_^2−^ and SO_3_^2−^, into the sodalite cage are unclear, given the fact that there are no steric obstacles to the presence of other, no less large groups (SO_4_^2−^, S_4_, S_6_) in the sodalite cage.

Perchlorate and permanganate anions in the sodalite-type compounds Na_8_[Si_6_Al_6_O_24_]_6_·(ClO_4_)_2_ and Na_8_[Si_6_Al_6_O_24_]_6_·(MnO_4_)_2_ transform into chloride and manganate on heating above 500 °C [152].

Heating nitrite sodalite, Na_8_(Al_6_Si_6_O_24_)(NO_2_)_2_, in air at ~1000 K results in the formation of nitrate and a significant expansion of the sodalite framework [174]. Heating in an inert atmosphere results in the decomposition of nitrite groups and formation of a Na_2_O-stuffed carnegieite phase in the 950–1100 K interval, in accordance with the reaction scheme

Na_8_(Al_6_Si_6_O_24_)(NO_2_)_2_ → 6(Na_2_O)_0.17_NaAlO_4_ + 2NO(gas) + 0.5O_2_ (gas).

Temperature-dependent properties of sodalite-type Na_8_(GaGeO_4_)_6_(NO_2_)_2_ were investigated by TG–DTA and in situ X-ray powder diffraction [121]. The Debye anharmonicity model was used for the evaluation of the thermal expansion coefficient. The temperature-dependent unit-cell volume behavior indicates intra-cage nitrite to nitrate oxidation around 850 K. This conclusion was confirmed by ex-situ FTIR spectroscopy.

Heating of the K_4_Na_4_(Al_6_Si_6_O_24_)(BH_4_)_2_ sodalite-type compound in air at 400–600 °C results in the formation of orthoborate groups [175]. FTIR data revealed a decrease in metaborate formation temperature close to 100 °C compared with the pure sodium tetrahydroborate sodalite which may be due to steric factors (the unit-cell a parameter is equal to 8.972 and 9.109 Å for pure sodium and K-rich tetrahydroborate sodalites, respectively).

## 4. Properties and Application of Sodalite-Related Materials

### 4.1. Ion Exchange; Immobilization of Heavy Metals and Redionuclides

Sodalite has long been regarded as a potential immobilization matrix for the chloride salt wastes arising from pyrochemical reprocessing operations. Similar techniques can be applied for the immobilization of lanthanides which are crystal-chemical analogues of actinides. The consolidation and densification of Sm-doped sodalite has been investigated with the aim of producing dense ceramic monoliths via conventional cold press and sinter techniques at temperatures below 1000 °C [176]. However, by the addition of a sodium aluminophosphate glass, dense Sm-sodalite ceramic monoliths can successfully be produced by sintering at temperatures as low as 800 °C and without pressing.

A sodalite-glass ceramic was prepared from zeolite 4A at temperatures of 750–850 °C and pressures > 96,500 kPa to immobilize radioactive nuclear waste containing plutonium alone or plutonium together with a variety of fission products [177]. Up to 35% of Pu in the waste form produced from Pu-loaded simulated fission-product salt may be segregated within the sodalite lattice.

Treatment and immobilization of technetium-99 contained in reprocessed nuclear waste and present in contaminated subsurface systems represents a major environmental challenge [178]. A possible way of treating anion-enriched reprocessed nuclear waste streams is to immobilize ^99^Tc and other radioactive isotopes in micro- and mesoporous materials. Perrhenate sodalite, Na_8_Al_6_Si_6_O_24_(ReO_4_)_2_, a crystal-chemical analogue of pertechnetate sodalite, Na_8_Al_6_Si_6_O_24_(TcO_4_)_2_, is often used for modeling behavior of the latter under various conditions of immobilization. Perrhenate sodalite demonstrates a high thermodynamic stability high temperature and pressure conditions, and has potential uses for immobilizing mobile Re and Tc during nuclear waste vitrification [179].

The largest negative enthalpy of formation from elements and the lack of structural water demonstrated that the perrhenate sodalite is more thermodynamically stable than all other anion bearing sodalites evaluated in [180]. The enthalpies of the solid-state reaction between nepheline and a sodium salt at 1000–1100 °C, which provides corresponding guest anion species in the resulting sodalite-type compound, was negative only for the ReO_4_ and NO_3_ bearing sodalites.

The potential for incorporation of perrhenate anion into mixed perrhenate/nitrate sodalite has been investigated and the mixed compounds Na_8_[Al_6_Si_6_O_24_](ReO_4_,NO_3_)_2_ have been synthesized [181,182]. However, calculated enthalpy and Gibbs free energy suggest that incorporation of nitrate anion, which is present in alkaline waste in relatively high concentrations, into the sodalite cage is favored over the incorporation of the larger perrhenate anion.

Phase stability of perrhenate sodalite was investigated in the temperature range from 13 to 1480 K [183]. Two phase transitions were observed. The first one (from a dynamically ordered compound to a SOD-type disordered phase, both with the *P*-43*n* symmetry) takes place at 218.6(1) K. The second phase transition, taking place at 442(1) K, results in a symmetry increase from *P*-43*n* to *Pm*-3*n* and is accompanied by a strong framework expansion.

Re-bearing sodalite was tested as a vehicle to transport perrhenate to high-level (HLW) and low-activity waste (LAW) glass stimulants [184]. The use of Re-sodalite improved the Re retention by 21% for HLW glass and 85% for LAW glass, demonstrating the potential improvement in Tc-retention if TcO_4_ were to be encapsulated in a Tc-sodalite prior to vitrification.

Based on the calculated values of Gibbs free energy and standard enthalpy for nitrate and perrhenate sodalites (the latter being a chemical surrogate for ^99^TcO_4_^−^ sodalite), it was concluded that incorporation of the nitrate anion into the sodalite cage is favored over the incorporation of ReO_4_^−^ due to the smaller ionic radius of NO_3_^−^ [182]. These results show that nitrate anion is strongly preferred for incorporation into the sodalite cage as compared to ^99^TcO_4_^−^.

The ReO_4_^−^ anion is a crystal-chemical analogue of TcO_4_^−^. The incorporation of ReO_4_^−^ into sodalite structure in the presence of other monovalent and divalent anions was studied in [185] in order to characterize ion selectivity in this process. It was shown that ReO_4_^−^ selectivity increased in the following order: Cl^−^ < NO_3_^−^ < MnO_4_^−^ and CO_3_^2−^ < SO_4_^2−^ < WO_4_^2−^. It was concluded that the difference in ionic radius between ReO_4_^−^ and coexisting anions does not exceed 12%. Otherwise, sodalite is not an effective ionite to be used for ReO_4_^−^ immobilization.

Incorporation of ReO_4_^−^ into ReO_4_-bearing sodalite, NO_2_-sodalite, mixed NO_3_-cancrinite/sodalite, and NO_3_-cancrinite in the presence of competing anions including OH^−^, NO_2_^−^, NO_3_^−^, and Cl^−^ was investigated [181]. In all experiments, feldspathoids were resistant to ion exchange with either NO_2_^−^ or NO_3_^−^. By analogy, it was concluded that ^99^TcO_4_^−^ does not compete well with the smaller ions.

Iodine sodalite, Na_8_Al_6_S_i6_O_24_I_2_, can be used to immobilize radioactive iodine from high-level radioactive wastes. A method of synthesis of iodine sodalite from zeolite-based iodine adsorbents is described in [186]. Zeolite 13X was used as the simulated sorbent. The leaching amount was found to be low (~10^−5^ to ~10^−4^ mol/L in sodium thiosulfate solution).

Silver exchanged zeolites A, X, and Y were used to occlude silver iodide at 400 °C [187]. Heating of silver-exchanged zeolites A and X to 900 °C caused the formation of iodide sodalite considered as a potential matrix for the immobilization of ^129^I, while silver zeolite Y formed an X-ray amorphous phase containing AgI. Silver zeolite A produced the best potential waste form, a monolithic sodalite with negligible porosity. An alternative preparative method yielded a denser and more stoichiometric AgI sodalite on sintering and hot isostatic pressing [188]. Differential normalized leaching rates of such a prepared iodide sodalite are 0.005–0.01 g·m^−2^·day^−1^ during the 7–14 day period [189]. This indicates that sodalite dissolution in natural groundwater, already saturated in these elements, will be very low. It was also shown [188] that iodine is released from AgI sodalite much more readily in reducing water than in ordinary water.

Iodine was captured from the vapor phase using a silver exchanged zeolite and converted to AgI-sodalite, Ag_8_[Al_6_Si_6_O_24_]I_2_, in hot isostatic pressing canisters [190]. This method could be applied for the capture of radioiodine from the vapor phase, followed by thermal conversion.

Different methods can be used to reprocess spent nuclear fuel containing chlorides of alkali, alkaline-earth, and rare-earth elements, as well as minor actinides and I^−^. In particular, the salt can be separated and put into multiple waste forms. Sodalite-type compounds as a host for model mixtures simulating the waste in the electrochemical separations process of nuclear fuel reprocessing have been synthesized by different methods involving solid-state reactions at 650–950 °C [191]. The best result was obtained at reducing temperatures using sodium aluminate and CS as reactants. In this case, the yield of a sodalite-type product with a density up to 91.4% of the theoretical value was 100%.

Sodalite has been investigated as an immobilization matrix for the salt waste generated by the pyrometallurgical processing of spent nuclear fuel containing ions of alkali, alkaline-earth, and some rare-earth metals [192]. Hydrothermal syntheses from kaolinite, metakaolinite, silica + sodium aluminate, and zeolite 4A were used to obtain materials for preliminary decontamination of the salt by ion-exchange. Sodalite synthesized from zeolite 4A is most suitable for routine operations of salt decontamination.

Surrogate sodalite-type materials for immobilization of radioactive waste, containing K, Br, I, Rb, Sr, Y, Cs, Ba, La, Ce, Pr, Nd, Sm, and Eu, with minor glass and NaCl admixtures, have been synthesized by hot mixing dehydrated zeolite 4A with corresponding salts at 550 °C and subsequent heating to 915 °C [124].

Immobilization of molten chloride salt waste arising from the recovering of uranium and plutonium through pyro-processing remains a topical problem. To this aim, sodalite and Si-Al-P-based materials were synthesized both in pure form and mixed with different glass matrices, then loaded with mixed chloride salts to study their retention capacities with respect to the alkali, alkaline-earth and rare-earth elements [193]. The matrices were characterized and leached for contact times up to 150 days at room temperature and at 90 °C. SEM analyses were also performed in order to compare the matrix surface before and after leaching.

In order to obtain materials for confinement of chloride salts wastes (CSW), pellets made of sodalite blended with commercial glass frit, partly with added chloride salts, were synthesized through dry pressing and subsequent thermal treatment at 800 °C for 100 h [194]. Nepheline, prepared from kaolinite and sodium hydroxide mixed with the simulated Li-K chlorides and glass frit, was used as an intermediate. The pellets prepared in such a way were heated and tested for release of different components. Normalized release rates after 28 days (g·m^−2^·day^−1^) at 90 °C for (LiK)-sodalite and CSW-sodalite are, respectively, 0.68 and 0.70 for Li, 0.14 and 0.12 for Al, 0.94 and 0.90 for Si, 0.53 and 0.36 for K, 0 and 0.18 for Rb, 0 and 0.01 for Cs, 0 and 0.11 for Sr, 0 and 0.96 for Ba, 0 and 0.02 for La, and 0 and 0.03 for Nd.

Aluminosilicate and aluminogermanate materials for immobilization and a simulated spent electrorefiner salt solution containing a mixture of alkali, alkaline earth, and lanthanide chlorides were synthesized from corresponding salt solutions, NaAlO_2_, and either Si(OC_2_H_5_)_4_ or Ge(OC_2_H_5_)_4_ [195]. The binders performed similarly with maximum densification achieved at temperatures of 750–850 °C.

The Na_2_O-B_2_O_3_-SiO_2_ glass binders for immobilizing LiCl-KCl eutectic salt waste in a glass-bonded sodalite waste form following electrochemical reprocessing of used metallic nuclear fuel were designed to generate waste forms with high sodalite content by annealing at 500 °C for 2 h [196]. The coefficients of thermal expansion for the glass phase in the glass-bonded sodalite waste are close to those of the sodalite phase which should result in lower probability of cracking in the full-scale monolithic ceramic waste form.

Sorption behavior of heavy metals (Cd^2+^, Pb^2+^, and Zn^2+^) from aqueous solution on iodate sodalite, Na_8_(AlSiO_4_)_6_(IO_3_)_2_, was examined, and the Freundlich and Langmuir isotherms were evaluated for iodate sodalite [197]. The maximum sorption capacity of iodate sodalite increased in the order Pb^2+^ > Cd^2+^ > Zn^2+^ (in the experiments carried out at room temperature, pH 5 and heavy metal ion concentration up to 100 mg/L, the adsorption capacities are 50, 43.5, and 38.5 mg/g, respectively).

Removal efficiency of diazinon pesticide by sodalite synthesized from perlite in an alkaline solution and modified with Cu_2_O nanoparticles was examined through a central composite design under the response surface methodology [198]. The optimum conditions included: 0.22 g adsorbent, 23.62 min contact time, at 29.28 °C. The percentage removal of diazinon in batch runs was 97.24%.

Ethylene oxide-based oligomers (polyethylene glycol, polyethylene glycol methyl ether, diglyme, tetraglyme, and polypropylene glycol) were used as alternative nonaqueous media for ion exchange of Na for Li in hydrosodalite and dehydrated sodalite [199]. The exchange degree was 88% to 99% after 3–5 exchange cycles at 100 °C, as determined by unit-cell parameters and elemental analysis.

### 4.2. Hydrogen and Methane Storage

Hydrogen is an important alternative energy carrier, because it is available in unlimited amounts in the form of water, and unlike hydrocarbons gives no CO_2_ as a byproduct of burning. The synthesis of new materials for hydrogen storage and their investigation of the release of hydrogen are important for advanced energy technologies. Zeolitic clathrates, including those whose structures are based on sodalite-type frameworks, are considered as prospective materials for hydrogen storage [200].

To achieve a large volume of pores and a high specific surface, various templates are used for the synthesis of such materials. Unfortunately, it is often very difficult to remove an organic template out of a zeolitic clathrate because of its limited pore diameter. Framework flexibility is also a significant factor in the synthesis of hydrogen clathrates because it plays an important role in phase transitions, absorption, and transport phenomena of absorbed molecules.

Two kinds of microporous materials for hydrogen storage can be distinguished—those absorbing molecular hydrogen and those containing extra-framework components which can decompose under mild conditions with the release of H_2_.

Molecular dynamics analysis of the self-diffusion of H_2_ in Na_3_Al_3_Si_3_O_12_ sodalite and thermodynamic limits on hydrogen storage in sodalite framework materials have been calculated in numerous works [200,201,202,203]. The results of the calculations indicate how the hydrogen storage capacity is linked to composition of the framework and extra-framework components. The theoretical limit of the content of H_2_ entrapped in sodalite cages is 4.8 wt. %, but real expected values are lower because of the counterbalancing effect of increased adsorption capacity with heavier framework atoms.

The SOD-type material Zn_6_[P_12_N_24_] with a nitridophosphate framework built by the vertice-sharing PN_4_ tetrahedra and extra-framework Zn^2+^ cations is suitable for hydrogen encapsulation in the sodalite cages because of the presence of vacancies in one quarter of the Zn sites [204].

Microporous compounds with metal–organic frameworks (MOFs) are also considered as advanced materials for hydrogen and methane storage [205,206,207,208,209,210,211], along with other microporous materials, including carbon nanotubes [212,213].

To enhance hydrogen and methane storage in MOF materials, most research efforts have focused on either increasing the MOF pore volume and surface area to enhance the gas storage capacity or incorporating functional moieties to improve the gas affinity in MOFs. It was shown that exchange of Et_2_NH_2_^+^ by Li^+^ cations in (Et_2_NH_2_)_3_[(Cu_4_Cl)_3_(TTCA)_8_]·26DEF (TTCA = triphenylene-2,6,10-tricarboxylate, DEF = N,N-diethylformamide) results in the formation of a microporous material which is beneficial hydrogen storage, whereas initial Et_2_NH_2_^+^ compound can be used for methane storage [214]. The H_2_ storage capacities at 77 K and 1 bar are 0.91 wt. % for the initial Et_2_NH_2_^+^ compound and 1.14 wt. % for its Li-exchanged analogue. The exchange of Et_2_NH_2_^+^ for Li^+^ results in the enhancement of the adsorption heat from 3.38 to 4.74 kJ/mol.

The reaction of H_3_TPB-3tz (TPB = 2,4,6-tri-p-(tetrazol-5-yl)-phenyl, tz = triazine) with CuCl_2_·2H_2_O in dimethyl formamide (DMF) affords the non-catenated sodalite-related compound Cu_3_[(Cu_4_-Cl)_3_(TPB-3tz)_8_]_2_·11CuCl_2_·8H_2_O·120DMF, while the reaction of H_3_TPT-3tz with MnCl_2_·4H_2_O or CuCl_2_·2H_2_O generates the catenated compounds Mn_3_[(Mn_4_Cl)_3_(TPT-3tz)_8_]_2_·25H_2_O·15CH_3_OH·95DMF and Cu_3_[(Cu_4_Cl)_3_(TPT-3tz)_8_]_2_·*x*(solvent) [205]. Catenation helps to stabilize the framework toward collapse upon desolvation, leading to an increase in the surface area from 1120 to 1580 m^2^/g and an increase in the hydrogen storage capacity from 2.8 to 3.7 excess wt. % at 77 K. The total hydrogen uptake in the Mn-compound reaches 4.5 wt. % at 80 bar and 77 K,

The sodalite-related compound Fe_3_[(Fe_4_Cl)_3_(BTT)_8_]_2_·22DMF·32DMSO·11H_2_O (BTT_3_^−^ = 1,3,5-benzenetristetrazolate, DMF = dimethylformamide, DMSO = dimethylsulfoxide) showed heat of hydrogen sorption of 11.9 kJ mol^−1^, leading to a total storage capacity of 1.1 wt. % at 100 bar and room temperature [215].

Hydrogen gas adsorption isotherms of M_2_(dobpdc) (M = Mg, Mn, Fe, Co, Ni, Zn; dobpdc^4−^ = 4,4′-dioxidobiphenyl-3,3′-dicarboxylate) measured at 77 and 87 K indicate strong H_2_ binding at low pressures, corresponding to the adsorption of one molecule per M^2+^ site [216]. Isosteric heats of adsorption indicate adsorption enthalpies ranging from −8.8 to −12.0 J/mol, with the trend Zn < Mn < Fe < Mg < Co < Ni.

Sodalite-related compounds containing extra-framework BH_4_^−^ anions are considered as potential sources of hydrogen [217,218]. The release of hydrogen from NaBH_4_ sodalite, Na_8_[AlSiO_4_](BH_4_)_2_, starts at temperatures between 150 and 550 °C; at higher temperatures, the endothermic signal sharply peaked at 670 °C (as a result of complete destruction of the sodalite framework and the formation of borate groups) was observed during heating the product in He atmosphere [219,220].

A total conversion of the BH_4_^−^ groups in nanocrystalline NaBH_4_ sodalite sample was reached at 500 °C, whereas a larger amount of the BH_4_^−^ anions remained stable within the microcrystalline sample under the same conditions [217]. This difference was explained by the smaller crystal size and the high amount of intergrown hydrosodalite-type phase in the nanocrystalline sample compared to the microcrystals. However, steric effect of the sodalite matrix could be an additional cause of the stabilization of BH_4_^−^ in the microcrystalline sample. This effect is weaker than in sodalites with larger extra-framework anions. To compare: the onset dehydrogenation temperature of fine-grained NaBH_4_ is below 300 °C [221], and a maximum decomposition rate of NaBH_4_ at 1 bar of H_2_ was observed above 500 °C [222].

Gallosilicate and aluminosilicate BH_4_-sodalites, Na_8_[GaSiO_4_](BH_4_)_2_ and Na_8_[AlSiO_4_](BH_4_)_2_, respectively, are rather stable under water treatment at room temperature but BH_4_^−^ anions partly decompose during 24 h treatment in water at 80 °C [223].

Hydrogen release by heating of NaBH_4_-bearing sodalites obtained by different methods of crystallization was studied for two samples with different crystal sizes [224]. Total hydrogen release was found during heating of the water-bearing autothermal product up to 550 °C. Under the same conditions, only a partial hydrogen release occurred from the nanocrystalline sample of crossover synthesis.

### 4.3. Gas Sorption; Sodalite-Related Membranes

Zeolite channels in sodalite have small (~2.8 Å) six-membered ring openings. This feature makes sodalite suitable for hydrogen separation from larger gas molecules. Hydrogen was encapsulated in low-silica, high-silica, and pure-silica sodalites and its release from these materials takes place at 380, 550, and 480 °C, respectively [225].

Diffusion of small molecules in sodalite-related materials is the key process determining sorption of gases and separation of different species using sodalite-based membranes. Transition-state theory was applied to calculate the zero-loading-diffusion coefficients of 12 gases (He, Ne, Ar, Xe, H_2_, N_2_, O_2_, CO_2_, SF_6_, CH_4_, CF_4_, and *i*-C_4_H_10_) in silica-sodalite [226]. The diffusion coefficients have been estimated.

Nanocrystalline sodalite-based gas sorbents were synthesized from sodium hydroxide, sodium aluminate and colloidal silica at 90 °C for 3 h in the presence of organic additives (ethanol, urea, and acetone) [227]. In the presence of ethanol, sodalite crystals with sizes of less than 100 nm were obtained. When both ethanol and urea were present in the reaction mixture, nanometer-sized crystals with the specific surface of 170–180 cm^3^/g (by nitrogen sorption) were produced. In the presence of acetone, sodalite crystals with sizes of 200 nm were formed. Synthesis of nanometer-sized sodalite with the BET specific surface area of 93.2 cm^3^/g without adding organic additives has also been reported [62].

A comprehensive experimental and computational investigation of the CO_2_ sorption properties of the metal–organic frameworks, M(BTT) (M = Cr, Mn, Fe, Cu; BTT^3−^ = 1,3,5-benzenetristetrazolate), which exhibit a high density of open metal sites capable of polarizing and binding guest molecules, has been carried out [228]. It was demonstrated that in situ neutron diffraction can provide insights into how to optimize existing metal–organic frameworks for CO_2_ adsorption. The high initial isosteric heats of CO_2_ adsorption are correlated with the presence of open metal sites.

High-purity nanometer-sized hydroxysodalite crystals with a perfect structure and suitable morphology are required for the developing of high-quality nanocomposite sodalite/ceramic membranes for the capture of CO_2_. Such crystals can be produced by the pore-plugging hydrothermal method. Effect of synthesis conditions on the technologically important characteristics of sodalite crystals prepared from a solution with molar composition of 5SiO_2_:0.5Al_2_O_3_:50Na_2_O:1005H_2_O was investigated at different temperature programs, ageing time, and interruption time [229]. The most pure and perfect hydroxysodalite crystals were obtained in the reaction with the total synthesis time of 3.5 h and two interruptions of one hour. On the other hand, ageing the precursor prior to pore-plugging hydrothermal synthesis could result in developing low-quality nanocomposite sodalite ceramic membranes.

A zeolite-like metal–organic framework [Zn(HL)]·DMA with sodalite topology solvothermally synthesized based on an N-rich aromatic ligand L = 4,5-di(1H-tetrazol-5-yl)-2H-1,2,3-triazole in dimethyl acetate (DMA) exhibits high CO_2_ uptake and selective CO_2_/N_2_ sorption capacity [230]. It was shown that the high percentage of open N-donor sites leads to the high uptake capacity for CO_2_, even in the absence of any NH_2_ groups and open metal sites. At saturation of 166.9 cm^3^/g and temperature of 195 K, the sorbent exhibits CO_2_ uptake capacity of 7.5 mmol/g or 23.8 CO_2_ molecules per cage. At 273 K and 298 K and saturation of 91.4 cm^3^/g, CO_2_ uptake was 91.4 cm^3^/g and 60.3 cm^3^/g, respectively.

The CO_2_ and N_2_ sorption properties of two highly crystalline sodalite-type metal–organic frameworks, Cu-BTT (BTT^3−^ = 1,3,5-benzenetristetrazolate) and Cu-BTTri (BTTri^3−^ = 1,3,5-benzenetristriazolate) have been investigated by in situ X-ray and neutron diffraction, which allowed visualization of the CO_2_ and N_2_ binding sites on the internal surfaces of the framework cages [231]. Slightly elongated distances between the open Cu^2+^ sites and surface-bound CO_2_ in Cu–BTTri can be explained by the fact that the triazolate ligand is a better electron donor than tetrazolate. Binding energies at each CO_2_ and N_2_ adsorption site were calculated in the frames of density functional theory. The numerical simulation predicts better separation performance for Cu–BTT. The calculated values for N_2_ adsorption degree by Cu–BTT and Cu–BTTri are, respectively, 4.3 and 1.8 mol/L.

The capture of SO_2_ by dehydrated Na_6_[AlSiO_4_]_6_ sodalite was investigated using the thermodynamics analysis and in frames of the first principles density functional theory [232]. The S atom of the SO_2_ molecule in the sodalite cage is coordinated by the O atom of the aluminosilicate framework and two oxygen atoms of sulfur dioxide are coordinated by Na^+^. Increasing the number of SO_2_ adsorbates results in the deformation of the framework accompanied by its expansion. The estimated saturation limit of SO_2_ sorption by Na_6_[AlSiO_4_]_6_ at room temperature and a low SO_2_ partial pressure of 10^−3^ bar is ~300 mg/g.

Microporous sodalite-type ML_2_ compounds (M = Pd^II^ or Cu^II^, HL = 2-hydroxypyrimidine or 4-hydroxypyrimidine) are thermally stable up to 330 °C in air and readily absorb H_2_, N_2_, CO, and CO_2_ [233]. A remarkable feature of the ML_2_ sodalites is the reversibility of their sorption isotherms; moreover, their enhanced ability for adsorption of molecules other than H_2_. Thus, these materials are suitable for gas storage and separation purposes.

The sodalite-type metal–organic compounds Cu_3_[(Cu_4_Cl)_3_(TPB-3tz)_8_]_2_·11CuCl_2_·8H_2_O·120DMF, Mn_3_[(Mn_4_Cl)_3_(TPT-3tz)_8_]_2_·25H_2_O·15CH_3_OH·95DMF, and Cu_3_[(Cu_4_Cl)_3_(TPT-3tz)_8_]_2_·*x*(solvent) synthesized in the reactions of 2,4,6-tri-p-(tetrazol-5-yl)-phenyl-s-triazine (H_3_TPB-3tz) with corresponding metal chlorides in the presence of dimethyl formamide (DMF) and other solvents readily absorb H_2_, showing hydrogen storage capacity from 2.8 to 4.5 excess wt. % [205].

Gaseous H_2_, N_2_, CO, and CO_2_ are readily incorporated in the porous, 3D sodalitic frameworks of sodalite-type metal–organic compounds ML_2_, where M = Pd^II^ or Cu^II^, L = 2-hydroxypyrimidine or 4-hydroxypyrimidine [233]. These materials are suitable for gas storage and separation purposes.

Membranes based on sodalite with a high porosity can be used for CO_2_ capture and CO_2_/H_2_ separation [234,235,236,237].

Thermal conversion of layered silicates was used to obtain silica sodalite with the surface area of 56.56 m^2^/g and pore volume of 0.181 cm^3^/g (by nitrogen sorption) [237]. These characteristics are significantly better than analogous values for hydrothermally-synthesized hydroxysodalite. Polysulfone membranes used with sodalite synthesized by topotactic conversion of layered silicates are asymmetric with a high mechanical strength.

Increasing the silica sodalite loading up to 10 wt. % enhanced the quality of the polysulfone membrane. Loading the polysulfone with silica sodalite enhanced its H_2_ permeance, but the separation factor significantly decreased as compared to pure polysulfone membrane [237].

In most works, α-alumina was used as a support for sodalite membranes synthesized by hydrothermal methods. A defect-free sodalite membrane has been produced on tubular porous α-Al_2_O_3_ supports by secondary growth method with vacuum seeding [238]. The thickness and the quality of sodalite membrane were controlled by the variation of the conditions of hydrothermal synthesis.

A pre-seeding secondary growth method allowed the synthesis of larger and homogeneous samples with a good reproducibility [239]. The membrane quality was evaluated by single gas permeation studies with both N_2_ and He. Gas transport at 20–160 °C was essentially governed by adsorption and surface diffusion, yielding a maximum He/N_2_ selectivity of 6.2.

A thin-film membrane based on sodalite and ceramic and supported on tubular α-alumina showed the H_2_ permeance of 7.97 × 10^−7^ mol/m^2^·s·Pa with a selectivity of 8.76 and at 100 °C and 0.48 MPa feed pressure [236]. This membrane was tested for H_2_/CO_2_ separation at 25 °C and feed pressure of 0.18 MPa. In the presence of carbon dioxide, the H_2_ permeance and selectivity reduced to 1.06 × 10^−7^ mol/m^2^·s·Pa 4.24, respectively.

Sandwich-type membranes comprising of graphene oxide and sodalite nanocrystals with different morphologies and sizes as an inner layer were prepared [235]. The membrane containing spheroidal sodalite nanocrystals showed higher H_2_ permeance of ~4003 GPU and H_2_/CO_2_ selectivity of ~45.5 at 25 °C in comparison to the pristine graphene oxide membrane (~1642 GPU and ~11.2, respectively).

Hydroxysodalite nanoparticles were prepared from amorphous aluminosilicate precursor containing disordered sodalite cages [240]. During the test of 50 h at high temperature of 200 °C, membranes prepared by transformation of the precursor impregnated in a graphene oxide matrix promoted both the high H_2_ gas permeance 4900 GPU and enhanced selectivity of 56 towards hydrogen for gaseous mixtures containing CO_2_ and H_2_O.

Stable membranes for H_2_ separation from N_2_ were prepared using nanosized sodalite crystals with extremely small crystallites (40–50 nm) synthesized from a colloidal suspension free of organic structural directing agent and uniformly dispersed in the polyetherimide matrix [241]. In a typical experiment with the H_2_/N_2_ gas mixture, the selectivity factor increased to 30.9 at 25–100 °C. Moreover, it is shown that the sodalite filler with a suitable aperture size of 2.8 Å would allow only H_2_ molecules to pass through and rejected N_2_.

Hydroxysodalite membrane synthesized on an α-Al_2_O_3_ support by a microwave-assisted hydrothermal method is a promising candidate for the separation of hydrogen from gas mixtures and important for the emerging hydrogen energy fuel system [242]. Gas permeation experiments showed that the hydrogen/n-butane permselectivity of the hydroxy-sodalite zeolite membrane was larger than 1000.

A double layered hydroxy sodalite membrane prepared on a tubular α-Al_2_O_3_ support in a hot-air oven was characterized by single gas permeation using He, N_2_, and SF_6_ [243]. The permeance ranged from 0.8 to 8 × 10^−8^ mol·m^−2^·s^−1^·Pa^−1^. The He/N_2_ selectivity was 2.5 to 2.7, which indicates that the selectivity was controlled by diffusion.

Membranes for gas separation were synthesized on seeded supports using synthesized hydroxysodalite powders and the effect of seed size was investigated [244]. With increasing the seed size, H_2_ and CH_4_ permeations decrease from 3224 to 279 (Barrer) and from 611 to 45 (Barrer), respectively, while the H_2_/CH_4_ selectivity slightly increases from 5.28 to 6.20.

Supported hydroxysodalite membranes were prepared on α-alumina disks using direct hydrothermal synthesis from a gel with the molar composition 5SiO_2_:Al_2_O_3_:50Na_2_O:1005H2O at 140 °C for 3.5 h [245]. The membranes were impermeable to N_2_ and He, which validated the absence of defects in the membrane structure. The membranes were used in dewatering several organic alcohol/water mixtures, organic alcohol being methanol, ethanol, 1-propanol, 2-propanol, 1-butanol, 2-butanol, 1-pentanol, and 2-pentanol. The membranes exhibited a water/alcohol separation factor larger than 106 and showed excellent thermal, mechanical, and operation stability.

A thin (2 μm thick) defect-free hydroxysodalite membrane was produced on α-Al_2_O_3_ supports using direct hydrothermal synthesis at 140 °C for 3.5 h and the molar ratio of the synthesis solution 5SiO_2_:Al_2_O_3_:50Na_2_O:1005H_2_O [246]. N_2_ and He permeance was as low below detection limit and 10^−11^ mol^−1^·m^−2^·Pa^−1^, respectively. Pervaporation experiments showed that the membrane produced by this method can be used for activated water permeation with water fluxes up to 2.25 kgm^−2^·h^−1^ at 200 °C.

Microporous hydroxysodalite membranes with different morphologies were synthesized via secondary growth technique with vacuum seeding on tubular α-Al_2_O_3_ supports at different synthesis conditions [247]. The permeation results using single gases (H_2_ and N_2_) confirmed the high quality of the hydroxysodalite zeolite membranes manufactured at seeding time of 60 s for the hydrogen purification under low temperatures (<200 K) and/or high pressures (>100 bars).

Permeation of hydrogen, nitrogen, and methane through a hydroxy sodalite membrane prepared hydrothermally on α-alumina support was studied at different pressure differences [248]. The support was prepared by gel-casting method and its surface was modified using a colloidal suspension which was prepared using α-Al_2_O_3_, ammonium polyacrylate and polyethylene glycol as a binder, with subsequent sintering at 1350 °C. It was concluded that the performance of hydroxysodalite on the modified support was not related to the kind of interactions on the interface between the zeolite layer and substrate.

A simple single-step method was developed for the production of homogeneous hydroxysodalite films with a thickness ranging from a few to 28 μm without seeding and by direct deposition during hydrothermal treatment on cheap and porous alumina substrates with 35% porosity and 500–600 nm of mean pore diameter [234]. The films were deposited from a reaction mixture with the stoichiometry 5SiO_2_:1Al_2_O_3_:50Na_2_O:1000H_2_O at 50–60 °C for 0.5 to 24 h. Good results in terms of hydrogen permeance and separation performance in experiments with H_2_ mixtures with methane, carbon dioxide, and nitrogen were achieved for 10–12 μm thick hydroxysodalite films. The membranes showed good stability in operating conditions up to 100 °C and 0.3 MPa of pressure difference across the membrane.

A hydroxy sodalite membrane synthesized hydrothermally, at 140 °C for 3.5 h, on the surface of a polished Al_2_O_3_ surface was employed in desalination of seawater at 2.2 MPa feed pressure over a temperature range of 30–200 °C [249]. The performance of the membrane was also tested with aqueous solutions of sodium chloride and nitrate. The degree of desalination of seawater exceeded 99.99% and the resistance of desalination water was equal to 18.2 MΩ. Membranes of this type exhibit a high water/acetone separation factor larger than 100. Similar membranes were also used to test pervaporation efficiency, acid and base stability at 473 K, 2.2 MPa, and different concentrations of the acid and the base [250]. Under these conditions, in the pH range of 2.9–13.7 the membrane showed a high stability and absolute selectivity towards water.

A high-performance 700 nm thick, compact and with almost no defect sodalite membrane over α-Al_2_O_3_ support was obtained by hydrothermal crystallization [251]. Using this membrane, a water/ethanol separation factor higher than 10,000 at a permeation flux of 4.4 kg·m^−2^·h^−1^ could be achieved.

In [252], macroporous clay support was used instead of α-alumina for the in situ growth of hydroxysodalite membranes. The membrane obtained after four cycles of zeolite deposition was used for separation of SDS surfactant and showed observed rejection in the range of 10–45%.

Desilication of high-alkali sodium aluminate solutions is necessary in the production of sodium aluminate hydrate. A hydroxysodalite compound synthesized from 23.65% NaOH, 15.27% Al(OH)_3_, 16.17% Na_2_SiO_3_·9H_2_O, and 44.91% deionized water by weight at 190–290 °C for 3 h has shown excellent desilication properties in highly caustic aluminate solution at 470–530 g/L Na_2_O, 80–220 g/L Al_2_O_3_, and 4.7–5.2 g/L SiO_2_ [253]. Desilication kinetics is described by the 2.47 order law with the activation energy of 94.04 kJ/mol. The mechanism of desilication may involve either adsorption on hydroxysodalite or hydroxysodalite crystallization.

The silica sodalite nanoparticles synthesized by topotactic conversion and partly functionalized using HNO_3_/H_2_SO_4_ (1:3) were infused into a polysulfone membrane via the phase inversion method to produce mixed matrix membranes [254]. The functionalization resulted in a reduction in surface area and increase in pore diameter of the nanoparticles (by N2 sorption at 77 K). The silica sodalite loaded membrane showed a rejection of 89% for Al^3+^ and <13% rejection for heavy metals tested.

Gas separation on nanocomposite membranes deposited on sodalite nanocrystals dispersed in BTDA-MDA polyimide matrices was studied and structurally characterized [255]. The sodalite support increases the permeability of the membranes for hydrogen, but decrease nitrogen permeability. At room temperature, the sodalite–polyimide membrane containing 35 wt. % sodalite has a H_2_ permeability of 8.0 Barrers and a H_2_/N_2_ selectivity of 281 which is significantly better than analogous data obtained with a polyimide membrane at the same testing temperature (7.0 Barrers and 198, respectively).

The adsorption properties of sodalite prepared hydrothermally from coal fly ash, NaOH, Al(OH)_3_, and sodium carboxymethyl cellulose at 60 °C for 5 h with subsequent heating at 700 °C for 6 h were evaluated by the removal of Pb^2+^ [256]. In comparison with commercial activated carbon particles, the as-prepared sodalite pellets exhibit good adsorption performances. The adsorption rate of sodalite pellets is very fast and the equilibrium adsorption capacity reaches 10 mg/g vs. 3 mg/g for activated carbon.

Modification of the surface of sodalite by polymers, including polyimide [255] or poly(vinyl alcohol) [257] results in significant changes in the interfacial and electrokinetic properties. In particular, modification of sodalite with polyimide increases the gaseous hydrogen permeability of the membranes, while nitrogen permeability decreases that result in the H_2_/N_2_ ideal selectivity of 198 [255].

The phase inversion method was applied to produce polyethersulphone-sodalite membranes loaded with variable content of sodalite particles [258]. Separation performance of the membranes towards metal ions (Mn^2+^, Pb^2+^, Cu^2+^, Al^3+^, and Mg^2+^ during acid mine drainage treatment were studied as a function of sodalite loading on the membrane performance. Both the membrane flux and the selectivity of the membrane towards all studied metal ions increased at increasing sodalite loadings. The membrane rejection varied from 6% for Mn^2+^ to 57.44% Pb^2+^.

### 4.4. Color Centers, Optical Properties, and Luminescence; Sodalite-Based Pigments

According to ab initio electronic and optical calculations using the linear augmented plane wave method within density functional theory, the absorption spectrum of pure sodalite is localized in the ultraviolet range between 40 and 250 nm [259]. In particular, it was shown that the O 2p states and Na 3s states play the major role in optical transitions. The experimentally measured optical band gap of pure hydrous hydroxysodalite is 4.5–4.7 eV which is close to the value of 4.16 eV found as a result of ab initio calculations and attributed to the localized state below Fermi level formed by the hydrogen bonds [260].

Density functional theory (DFT) calculations including state-of-the-art two component time-dependent DFT implementation for hybrid exchange correlation functional have been applied to predict the structure and properties of the PbS quantum dots encapsulated in the sodalite-type host, a system reported to exhibit extremely high nonlinear optical properties [261]. The results obtained are in good agreement with experimental optical adsorption spectra. The sodalite framework can be regarded as a confining dielectric matrix that only modulates the optically active states of the PbS quantum dots.

Blue ultramarine pigment is known for a long time. Renaissance artists produced ultramarine from the natural sodalite-group mineral lazurite to prepare a beautiful blue paint which did not fade in the sun and was not damaged by dampness or fire. Later, blue S-bearing sodium silicate pigment similar to natural ultramarine has been synthesized [262].

For a long time, the term “lazurite” remained ambiguous and was applied to a sulfide-dominant sodalite-group mineral, all blue sodalite-group minerals, or different rocks from lapis lazuli deposits containing these minerals. In the list of minerals of the International Mineralogical Association (http://cnmnc.main.jp/ accessed on 21 August 2024) the idealized formula of this mineral is given as Na_3_Ca(Si_3_Al_3_)O_12_S. This formula means that lazurite is an aluminosilicate sodalite-group mineral in which a sulfide species dominates over other extra-framework anions. However, the S^2−^ anion, which is implied in this formula, is not a blue chromophore, unlike the S_3_^•−^ radical anion. However, in the paper [5] cited by the IMA list of minerals a SO_4_-dominant sodalite-group mineral is described with the name “lazurite” while its formula (Na,Ca)_8_(Al_6_Si_6_O_24_)(SO_4_,S)_2_ corresponds to sulfide-bearing haüyne variety.

The following alternative simplified formulae of lazurite and lazurite-related minerals have been suggested by different authors based on chemical, structural and spectroscopic data of the studied samples:

Na_6_Ca_2_(Al_6_Si_6_O_24_)(SO_4_)_1.4_S_0.6_ [15]; (Na,Ca)_7–8_(Al_6_Si_6_O_24_)(SO_4_,S,Cl)_2_·H_2_O [263]; (Na,Ca)_8_(Al_6_Si_6_O_24_)(SO_4_,S)_2_ [264]; Na_8_Al_6_Si_6_O_24_S [265].

Empirical formulae of most deep blue, sulfide-rich and sulfate-depleted sodalite-group minerals calculated based on [(Si,Al)_12_O_24_], with SO_4_^2−^ and/or S^2−^ are not charge-balanced, with a significant excess of negative charge.

Currently, the term ultramarine is applied to a group of S-bearing sodalite-related pigments which can be not only blue but also violet, lilac, red, green, yellow, and even colorless [266,267].

The acyclic neutral species S_2_, S_3_, and S_4_ absorb visible light while all larger molecules, including S_6_, are cyclic or polymeric and absorb only in the near UV with a wing into the violet region of the visible spectrum [268]. Therefore, these species are more or less yellow at 25 °C. Ab initio calculations in frames of the MO theory have shown that the S_4_ isomer absorbing at 530 nm is of C_2v_ symmetry (*cis*-S_4_) and the one absorbing near 625 nm is of C_2h_ symmetry (*trans*-S_4_). However, it is to be noted that these values were obtained for free isolated molecules, whereas their occurrence in sodalite cages may result in significant shifts of absorption bands.

There is controversy over the identity of the chromophore in ultramarine red as either the neutral S_4_ allotrope or the S_4_^•−^ radical anion [269].

Based on spectroscopic data, it was concluded that the S_3_^•−^ radical anion is the cause of deep blue color of lazurite and ultramarine [270]. Numerous subsequent studies confirmed this conclusion [7,165,269,271,272,273,274].

Initially, lazurite was considered as a sodalite-group mineral with sulfide sulfur occurring mainly in the form of the S^2−^ anion [5,275]. However, empirical formulae of most deep blue, sulfide-rich, and sulfate-depleted sodalite-group minerals, calculated based on [(Si,Al)_12_O_24_] with SO_4_^2−^ and/or S^2−^, are not charge-balanced, with a significant excess of negative charge. This means that a significant part of sulfur in these minerals occurs as S_3_^•−^, S_2_^•−^, and SO_3_^•−^ radical anions and/or HS^−^ and different conformers of the chain-like neutral S_4_ molecule (all these species were detected in sodalite-group minerals from gem lazurite deposits using a combination of spectroscopic methods [17,18,20,21,22,23,24,25,26,27]. The correct idealized formula of lazurite accepted by the IMA Commission on Mew Minerals, Nomenclature and Classification in 2021 is Na_7_Ca(Al_6_Si_6_O_24_)(SO_4_)S_3_^•−^·H_2_O; the charge-balanced empirical formula of the holotype sample from the Malo-Bystrinskoe gem lazurite deposit, Baikal Lake area is (Na_6.97_Ca_0.88_K_0.10_)_∑7.96_[(Al_5.96_Si_6.04_)_∑12_O_24_](SO_4_^2−^)_1.09_(S_3_^•−^)_0.55_S^2−^_0.05_Cl_0.04_·0.72H_2_O. [20].

It was shown that S_3_^•−^ is characterized by a solvent-dependent absorption band in the range 595–620 nm, whereas S_2_^•−^ (yellow) has λ_max_ ≈ 400 nm [276]. S_3_^•−^ is a very strong chromophore: even sodalite-group minerals containing ~0.01 S_3_^•−^ groups per formula unit have an intense blue color [18].

**Figure 20:**
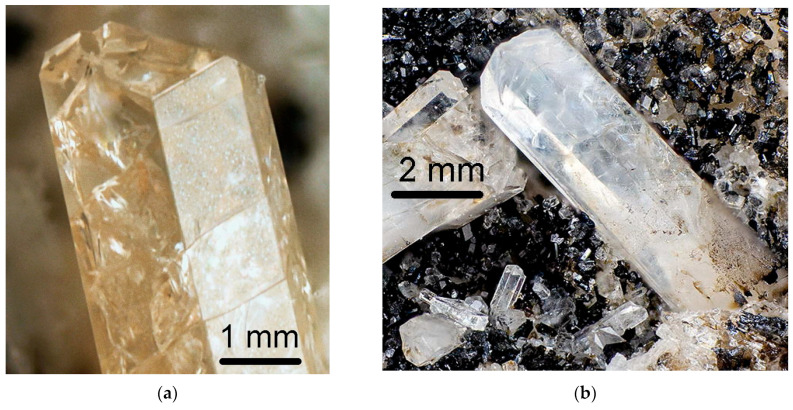

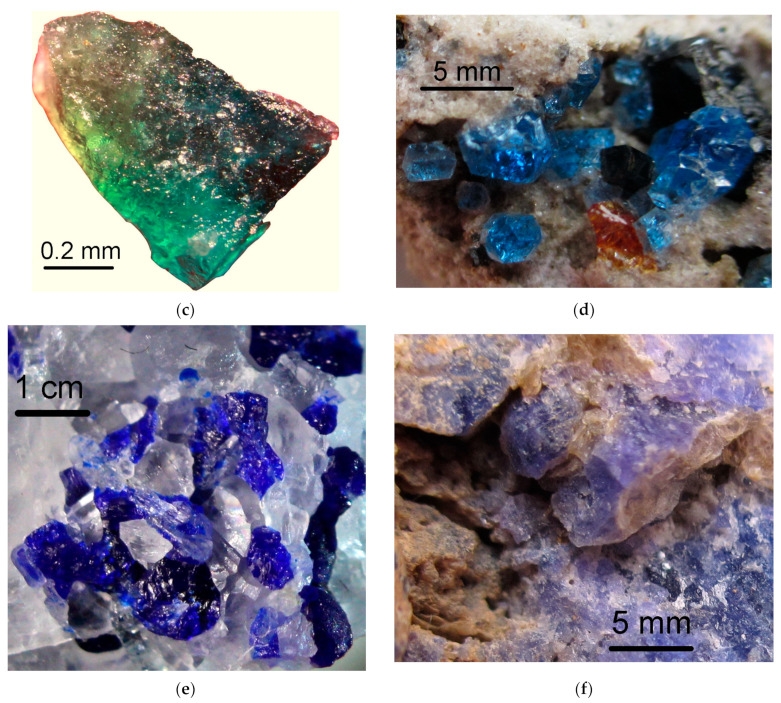
Examples of sodalite-group minerals containing various polysulfide chromophores: (**a**) bolotinaite (S_2_^•−^, yellow), (**b**) nosean without chromophores, (**c**) slyudyankaite (combination of S_6_, yellow, S_3_^•−^, blue and *cis*-S_4_, red; the presence of *trans*-S_4_, green is not excluded), (**d**) haüyne (combination of S_3_^•−^, blue and S_2_^•−^, yellow), (**e**) intermediate member of the haüyne–lazurite solid–solution series (S_3_^•−^, blue) and (**f**) S_4_-rich haüyne (combination of *cis*-S_4_, red and minor S_3_^•−^, blue). The color centers were identified using a complex of spectroscopic methods [18,21,23,24,25,26].

**Figure 21:**
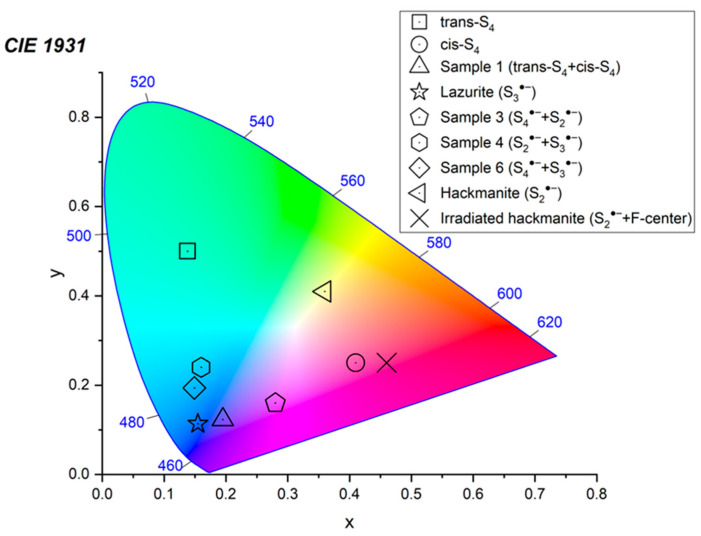
CIE (Commission Internationale de l’Éclairage) color space chromaticity diagram for S-bearing aluminosilicate sodalite-group minerals [24].

Polysulfide groups are the main chromophores in natural minerals belonging to the sodalite group. These groups can be identified by means of Raman spectroscopy, ESR, and absorption spectroscopy in the visible, ultraviolet, and near infrared ranges. Figure 20 shows several examples of sodalite-group minerals containing different color centers. Corresponding color diagram is shown in Figure 21.

**Figure 22:**
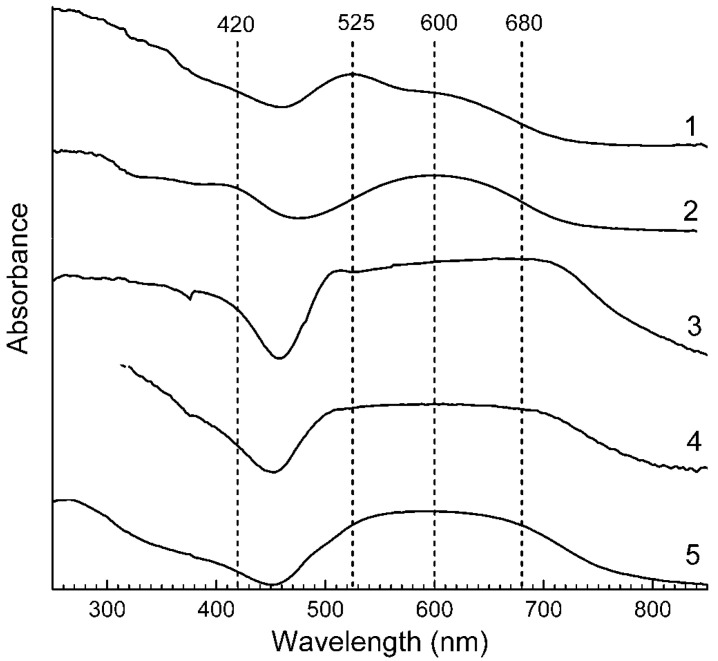
UV-Vis absorption spectra of differently colored sodalite-group minerals [27]: (1) lilac S_4_-bearing haüyne, (2) light blue (with greenish hue) SO_3_^2−^-, S_2_^•−^-, and S_3_^•−^-bearing haüyne, with a minor amount of S_4_ groups (3) blue S_3_^•−^-bearing haüyne, (4) deep-blue S_3_^•−^-rich haüyne, and (5) dark blue lazurite with 0.55 S_3_^•−^ groups per formula unit.

**Figure 23:**
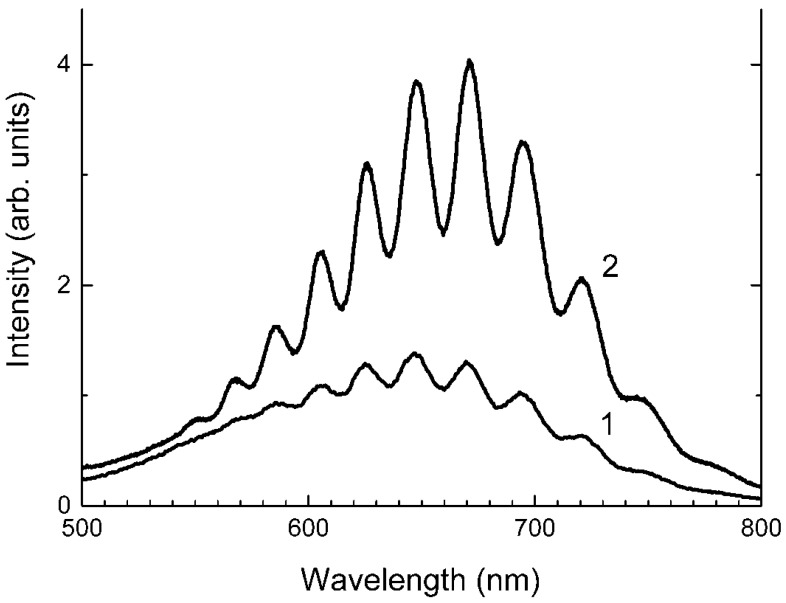
Photoluminescence spectra of the light greenish-blue haüyne sample under 405 nm excitation measured at room temperature (curve 1) and 77 K (curve 2) [27].

Five differently colored sodalite-group minerals from gem lazurite deposits have been studied by diffuse-light optical absorption spectroscopy of in the visible and near UV ranges (UV-Vis spectroscopy) and luminescence spectroscopy ([27], Figure 22 and Figure 23). The UV-Vis spectrum of lazurite (curve 5 in Figure 22) contains a broad absorption band with a maximum at about 600 nm corresponding to absorption of the S_3_^•−^ radical anion. In the absorption spectra of blue S_3_^•−^-bearing haüyne samples (curves 3 and 4 in Figure 22), a broad table-like absorption band is observed in the 450–800 nm region. In the wavelength range below 450 nm, a smooth absorption rise is observed.

The absorption spectra of the lilac and light greenish-blue haüyne samples (curves 1 and 2 in Figure 22) are more complex. In the spectrum of former sample, a band at 525 nm is observed. This band was assigned to the cis-S_4_ neutral molecule (the C_2v_ conformer [277,278]). Another band (at 600 nm) corresponds to the trisulfide radical anion. The absorption spectrum of the latter sample contains bands at 420 and 600 nm and a weakly pronounced maximum at 680 nm. The band at 420 nm corresponds to S_2_^•−^ radical anions. Upon excitation with 420 nm radiation, both samples exhibit characteristic luminescence with a vibrational structure in the range of 530–700 nm. When the sample is cooled to 77 K, the vibrational structure becomes more pronounced (Figure 23). The distance between the components of the vibration structure of about 590 cm^−1^. The observed luminescence is due to the presence of S_2_^•−^ centers [279].

Color mechanisms of sodalite-related beryllosilicates, as well as some sodalite-group minerals which do not contain polysulfide groups, are significantly different from those of their aluminosilicate counterparts with S_n_ and S_n_^•−^ groups.

Single crystals belonging to the helvine–genthelvite solid–solution series, (Mn,Zn)_8_(Be_6_Si_6_O_24_)S_2_, and showing homogenous coloration in the range pale yellowish green to reddish yellow have been investigated by means of optical absorption spectroscopy [280]. The sharpness and energy of the band at ~23,800 cm^−1^ demonstrates that it represents the field-independent ^6^*A_1_*(*S*) → ^4^*E_g_*^4^*A_1g_*(*G*) transition in Mn^2+^ at a tetrahedrally coordinated site. The remaining absorption bands at ca. 21,100, 22,600, 26,800, 28,400, 28,800 cm^−1^ are assigned to the spin-forbidden electronic *d-d* transitions ^6^*A_1_*(*S*) → ^4^*T_1g_*(*G*), ^6^*A_1_*(*S*) → ^4^*T_2g_*(*G*), ^6^*A_1_*(*S*) → ^4^*T_2g_*(*D*), ^6^*A_1_*(*S*) → ^4^*E_g_*(*D*) and ^6^*A_1_*(*S*) → ^4^*T_1g_*(*P*) in ^IV^Mn^2+^, respectively.

**Figure 24:**
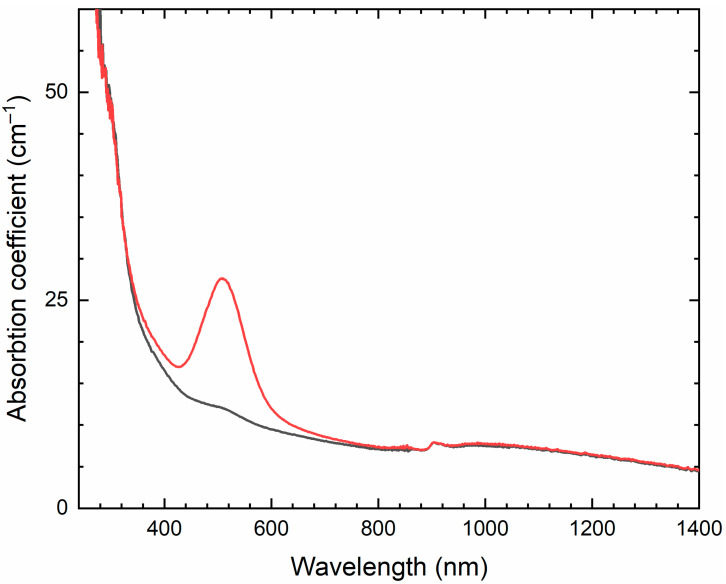
Absorption spectrum of tugtupite: almost colorless sample (black curve) and purple after irradiation with UV light (red curve).

Irradiation of tugtupite with a UV light with a wavelength <450 nm results in the change of its typical pale pink color to deep purple and the appearance of an intense light absorption at ~520 nm [26] (Figure 24). Simultaneously, an intense ESR signal appears. Subsequent irradiation with light having a wavelength of 500–600 nm results in the disappearance of the purple color. This phenomenon is the cause of color fading of tugtupite in sunlight.

**Figure 25:**
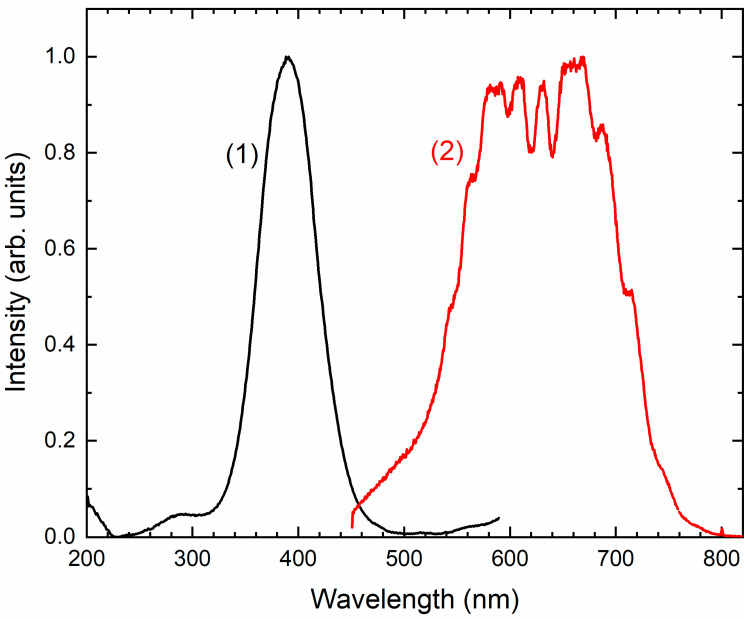
Excitation spectrum of tugtupite monitored at 605 nm (curve 1) and luminescence spectrum of tugtupite monitored at 400 nm (curve 2).

Tugtupite studied in [26] shows a strong luminescence in the range of 600–800 nm upon excitation with a UV radiation with λ = 400 nm related to the presence of S_2_^•−^ radical anions. The luminescence spectrum has a vibrational structure with a distance between the components of about 620 cm^−1^ [26] (Figure 25).

Color change under light exposure in known as the phenomenon of photochromism. In the case of tugtupite, this phenomenon is studied insufficiently and its explanations are ambiguous [281]. Electron transfer from S_2_^2−^ to a Cl vacancy upon UV irradiation may result in the formation of F_Cl_^−^ centers (captured an electron), which have absorption bands near 550 nm [282]. A similar point of view is shared by the authors of other works [283,284]. When tugtupite is heated above 700 °C, photochromism disappears, but the photoluminescence associated with S_2_^•−^ retains, unlike hackmanite.

The mechanism of coloration and discoloration of S_2_^2−^-containing sodalite (hackmanite) was studied by TD-DFT and post-Hartree-Fock (SAC-CI) ab initio calculations, which confirmed charge transfer between the S_2_^2−^ ion and a Cl vacancy [285]. The high stability of the colored state of hackmanite was explained by a significant electronic reorganization stabilizing the F center.

Spectroscopic properties of point defects in sodalite were investigated using time-dependent density functional theory [286]. The F-center absorption spectrum and S_2_^•−^ impurity fluorescence spectrum were simulated by considering different electrostatic environments around the cluster and by coupling the electronic transition with vibrations obtained at the periodic boundary condition level. These results highlight the influence of vibronic coupling in these spectra and of the confinement in the case of S_2_^•−^ fluorescence. The calculated S_2_^•−^ emission spectra in the range of 450–1000 nm show a distinct vibrational structure with the period of ~600 cm^−1^ (the S–S stretching mode) in accordance with experimental data [25]. The model developed in this work was applied to different chemical compositions of sodalite structures, and a color simulation of the trapped electron as a function of the mineral chemical formula gave an idea of the range of color accessible by tuning the sodalite composition.

Color variations of helvine and Mn-bearing genthelvite (from yellow to brown and reddish) could not be explained by single ion absorption of Mn^2+^ having tetrahedral coordination. The absorption spectra of these minerals contain five bands in the range from 21,100 to 28,400 cm^−1^ with different ratios of their intensities. The absorption coefficients of these bands are proportional to the square of the manganese concentration in the samples. It was shown that these bands are partly due to the exchange coupled ^IV^Mn^2+^–^IV^Mn^2+^ pairs [280].

Hackmanite is a sulfide-bearing sodalite variety which turns purple under UV irradiation, and the color fades back to white in a few minutes under regular white light. The absorption band at ~540 nm, which gives the hackmanite pink to purple color, is associated with F-center, which is a chlorine vacancy on which an electron is localized. It is generally believed that the photochromism of hackmanite is due to the trapping of electrons from sulfide ions by chlorine vacancies, thus forming F-centers [287,288,289].

**Figure 26:**
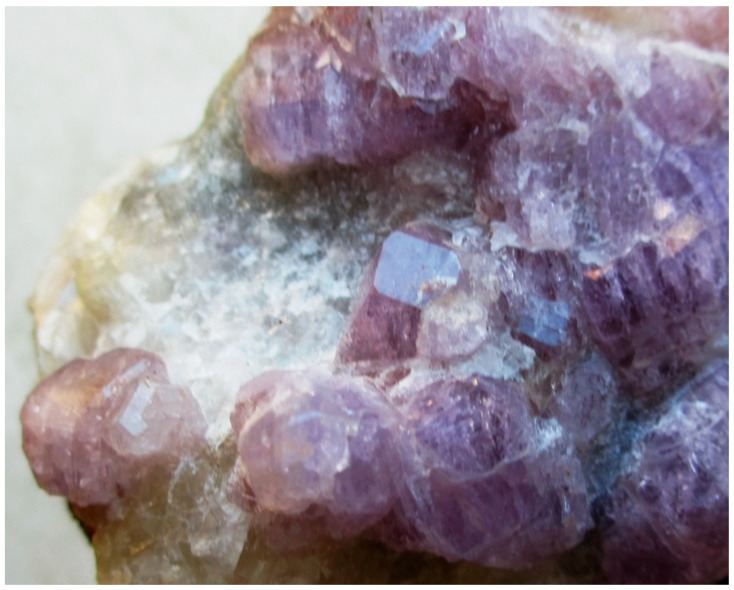
Initially almost-colorless hackmanite from Sar-e Sang, Afghanistan after exposure by visible light.

When hackmanite is placed in darkness or visible light, the electrons are gradually removed from the vacancy and the color disappears [289]. However, in some samples, which are initially colorless, violet color appears and becomes more intensive during exposure by visible light (Figure 26). In darkness, this color disappears over several hours or several days.

Hackmanite was studied by sulfur K X-ray absorption near edge structure (XANES) spectroscopy [290] and it was concluded that this sodalite variety contains reduced sulfur forming various groups, and depending on localities additionally can sulfate sulfur.

The sodalite-group minerals hackmanite and tugtupite, Na_8_[Be_2_Al_2_Si_8_O_24_](Cl,S)_2−*x*_ exhibit tenebrescence (reversible photochromism) and photoluminescence. These features were generally attributed to the presence of sulfide species within their structures [284], but how these optical properties might be affected by intercalating additional amounts of sulfur into their structures was unknown for a long time.

In the ESR spectrum of irradiated tugtupite, which has a saturated purple color, an ESR signal with *g*_1_ = 2.020 and *g*_2_ = 2.001 is observed [26]. This signal was previously observed in the ESR spectra of minerals of the sodalite and cancrinite groups and was attributed to the S_4_^•−^ radical anions [24,25]. Moreover, such radical anions have absorption bands in the region of 500–540 nm, and samples containing S_4_^•−^ and S_2_^•−^ radical anions have a color close to that of tugtupite [24]. During heating, the decomposition of S_4_^•−^ radical anions occurs with a simultaneous increase in the concentration of S_2_^•−^ [13]. This explains the disappearance of photochromism in the heated samples of tugtupite.

Photoluminescent sodalites are generally enriched in S compared to non-photoluminescent samples, although few samples being S-poor still show photoluminescence. A reduced intensity in cathodoluminescence was observed at high S contents for some samples, showing that S can act as cathodoluminescence quencher [291]. The authors of the cited work propose a saturation of F-centers to explain tenebrescence at different S contents.

Excitation of sodalite with either LW- or SW-UV light usually results in a yellow-orange and orange-red photoluminescence, respectively [292,293,294]. Yellow-orange photoluminescence has been ascribed to the disulphide anion S_2_^2−^ [295,296,297] or the radical anion S_2_^•−^ [294,297,298].

Heat-treated sodalite samples exhibited green and red luminescence with maximum intensity at 496 and 687 nm, respectively, under 264 nm excitation at room temperature [294]. The green luminescence efficiency of the sample heat-treated at 900 °C was 6.5 times higher than that of unheated natural sodalite. At 8.5 K, the green luminescence showed a vibronic structure. The luminescence lifetimes of the green and red luminescence at room temperature were 2.1 and 5.1 ms, respectively. It was proposed that the origin of the green luminescence is Mn^2+^ replacing Na^+^, and that of the red luminescence is Fe^3+^ replacing Al^3+^. However, the latter assumption seems questionable taking into account that no green luminescence is observed for Fe^3+^-bearing sodalite-group minerals which do not contain sulfide sulfur.

Luminescence spectra of different sodalite varieties and tugtupite have been collected during X-ray irradiation as a function of temperature between 20 and 673 K [299]. The emission bands observed in these samples were assigned to F-centers (360 nm), paramagnetic oxygen defects (400 and 450 nm), S_2_^•−^ radical anions (620 nm), and Fe^3+^ cations having tetrahedral coordination (730 nm). Yellow luminescence (550 nm) was tentatively attributed to Mn^2+^.

Although sulfur is a crucial element for luminescence in natural sodalites, other luminescence centers, e.g., Fe^3+^, Mn^2+^, Eu^2+^, and Ce^3+^ were found in some sodalite samples [292]. Excitation and emission spectra of Nd^3+^ activated sodalite-type materials Ln_4_(Al_8_Si_4_O_24_)(WO_4_)_2_ in the range 400–1100 nm are given in [300] and explained by the transitions ^4^*I*_9/2_ → ^4^*F*_5/2_, ^4^*F*_9/2_ → ^4^*I*_9/2_ and ^4^*F*_9/2_ → ^4^*I*_11/2_.

A significant near-infrared emission efficiency is observed in the haüyne-type materials Eu_4_(Al_8_Si_4_O_24_)(MoO_4_)_2_ [301]. In this structure, a close proximity of rare earth ions and MoO_4_^2−^ is realized, which leads to quantum efficiencies of 55% upon excitation of the O → Mo ligand-to-metal charge transfer at 254 nm. In the cited work, similar emission spectra were obtained for Eu_4_(Al_8_Si_4_O_24_)(WO_4_)_2_.

Tenebrescence is sensitive to many features such as composition and structure. Contrary to other studies, it was shown that especially the strongest tenebrescent samples showed extremely low S contents and also the lowest overall amount of trace elements. It was shown that the most efficient tenebrescent sodalites have the smallest unit-cell dimensions and a strong link between the atomic structure and the formation of F-centers [291].

Green luminescence phosphors were synthesized by doping natural sodalite with Tb^3+^ in a high-temperature reaction with TbF_3_ in melt, at 1100° [302]. The main phase in the product was sodalite, and minor content of nepheline, NaAlSiO_4_, was created. The excitation bands at 174 and 222 nm were assigned to the absorption by the host crystal and the spin-allowed 4f^8^–4f^7^5d transitions within Tb^3+^, respectively. The emission spectra under VUV–UV light excitation consisted of a series of narrow bands corresponding to the ^5^D_3_ → ^7^F_J_ and ^5^D_4_ → ^7^F_J_ transitions within Tb^4+^. The predominant band at 541 nm corresponds to the ^5^D_4_ → ^7^F_5_ transition. The lifetime of luminescence corresponding to these transitions was from 1.2 to 2.8 ms. The chromaticity coordinate of luminescence from Tb^3+^-doped sodalite is (0.287, 0.301) (see Figure 1 for comparison).

Natural blue sodalite stone from the State of Bahia, Brazil has shown thermoluminescence peaks at 110, 230, 270, 365, and 445 °C [303]. A correlation between the ESR *g* = 2.01132 line and the 365 °C thermoluminescence peak was observed. In order to explain this phenomenon, a model is proposed in which a Na^+^ ion acts as a charge compensator when an Al replaces Si in the framework.

Under short UV (248 and 266 nm) excitation at 300 K, the main visual luminescence color of tugtupite, Na_8_Al_2_Be_2_Si_8_O_24_Cl_2_, is red with green additions [293]. The luminescence center responsible for red emission is characterized by a relatively broad emission band, peaking at 670 nm, with long decay times of 7–8 ms. In addition, two types of green luminescence centers were detected, both characterized by narrow emission bands peaking at 495 and 510 nm with long decay times of s = 5–6 ms and excited mainly in a broad UV band peaking at 300 nm. Under short UV (248 and 266 nm) excitation, several ultraviolet and violet emission bands appear as well.

The green structureless luminescence of tugtupite peaking at 510 and 494 nm was assigned to the Mn^2+^ center substituting Na^+^ [293]. However, the assignment of red luminescence of tugtupite made in the cited work is questionable: Fe^3+^ substituting Al in the framework is a common case among sodalite-group minerals, but most of them do not show red luminescence under UV radiation. Violet luminescence bands peaking at 365 and 410 nm with decay times of approximately several μs detected in tugtupite were assigned to the Eu^2+^ impurity luminescence center [293]. Such an interpretation is supported by the fact that impurities of Eu in quantities up to 500 ppm have been found in natural tugtupite. Yellow-orange luminescence of tugtupite has the same cause as in S-bearing sodalite and is related to the presence of S_2_^•−^ radical anions substituting Cl^−^.

Optical and laser excitation spectroscopy study of the Mn^2+^-doped compound Zn_4_B_6_O_13_ with the sodalite-type framework topology were carried out in the temperature range 10–300 K as functions of Mn^2+^ concentration [304]. The green emission observed at ~540 nm may be due to the exchange coupled ^IV^Mn^2+^–^IV^Mn^2+^ pairs and the strong absorption band at 534 with vibrational satellites at 538, 548, and 560 nm was assigned to the ^4^T_1_ → ^6^A_1_ transition. The emission of the vibrational satellites was observed.

### 4.5. Application of Sodalite-Related Materials for Organic Synthesis

A hydroxy sodalite membrane prepared by means of hydrothermal synthesis on the inner surface of an α-alumina tubular support was applied in the esterification reactions to remove water and shift thermodynamic equilibrium towards ester formation [305]. The membrane showed 100% selectivity towards water and retained its stability under the reaction conditions.

The performance of a sodalite membrane reactor in the conversion of methanol to olefins (MTO process) was evaluated for ethylene and propylene production with in situ steam removal using a three-dimensional computational fluid dynamic technique [306]. The modeling results showed that the sodalite MR in the MTO process had higher performance in methanol conversion compared to the fixed-bed reactor (methanol conversion of 97% and 89% at 460 °C for sodalite and fixed-bed reactor, respectively).

The main area of application of sodalites in organic synthesis is their use as catalysts and catalyst supports.

Tetrahydrobenzo[*b*]pyrans can be used as pigments and photoactive materials and exhibit biological and pharmacological activities such as anticoagulant, anti-cancer, anti-anaphylactic, spasmolytic, and diuretic. Hydroxysodalite that is the waste-product of zeolite manufacturing process was used as an efficient and a very inexpensive catalyst in preparation of tetrahydrobenzo[*b*]pyrans in three-component reactions of aldehydes, alkylnitriles, and dimedone [307]. Under neutral conditions, the reactions proceed with excellent yields, short reaction time, simple work-up, and recovery and reusability of the catalyst.

As an important chemical precursor, epichlorohydrin is used to synthesize epoxy resins, pesticides, and plasticizers [308,309]. Mesoporous sodalite with a surface area reaching 295 m^2^/g was prepared hydrothermally from NaAlO_2_, Na_2_SiO_3_·9H_2_O, and chlorides in the presence of NaOH using a bridged polysilsesquioxane monomer as the mesoporogen. The results from [66] showed a high catalytic activity and selectivity towards performance of 1,3-dichloropropanol during the preparation of epichlorohydrin.

Mesoporous sodalite samples prepared using a bridged polysilsesquioxane monomer as the mesoporogen in the presence of chloride-containing salts (NaCl, KCl, and NH_4_Cl) were used to evaluate the alkali catalytic performance of 1,3-dichloropropanol during the preparation of epichlorohydrin [66]. The results showed that a larger surface area and pore volume were beneficial to the conversion and selectivity of this reaction.

The catalytic activity of mesoporous sodalite synthesized using an organosilane surfactant showing about ten-fold higher surface area and four-fold larger pore volume, as compared with sodalite with solely microporous structure, was evaluated for the base catalyzed reactions involving bulky and small substrates, *viz*. Knoevenagel condensation, Claisen–Schmidt condensation in liquid phase, and acetonylacetone cyclization in vapor phase [310]. The catalyst showed a good recyclability, a higher activity, and longer lifetime than CsNaX and KAlMCM-41 which were used previously as the catalysts in these reactions. It also exhibited high activity and stability toward deactivation in a vapor phase acetonylacetone condensation reaction, and therefore could be used for longer reaction time, whereas CsNaX deactivated rapidly due to coke formation. It can also be used as a stable support for further surface modifications.

The activity of nanocrystalline sodalite with a BET surface area of 73 m^2^/g and an average crystallite diameter of 47 nm, prepared hydrothermally from sodium metasilicate, sodium aluminate, and sodium chloride at 150 °C, in the reaction of epoxidation of α,β-unsaturated ketone with hydrogen peroxide has been investigated [311]. Nanocrystalline sodalite was found to play a role in the pH adjustment of the liquid phase, which was required for the reaction to proceed.

A synthetic method has been established for the fabrication of sodalite monoliths with a hierarchical pore system to be used for basic catalytic reactions under flow [312]. The monoliths were synthesized by pseudomorphic transformation of a meso-/macroporous silica monolith. For this, a pseudomorphic transformation of a Nakanishi-type silica monolith was developed into the sodalite-type counterpart, while maintaining its complete crack-free morphology. The silica monolith was impregnated with a solution prepared by dissolving NaOH, NaAlO_2_, and tetrapropylammonium hydroxide. The impregnation was carried by hydrothermal treatment at 150 °C for 18 h. The monolith was then washed with water, dried, and calcined at 550 °C for 8 h. These materials could replace catalysts that are damaged during catalysis, especially for those where water is formed. The monolith was successfully tested in Knoevenagel reaction, a C–C bond forming reaction, in liquid phase involving bulky substrates, which is a key step in the preparation of several pharmaceutics. The pseudomorphic transformation of monoliths would contribute to the development of flow processes not only in catalysis, but also for different processes like ion-exchange, trapping of radioactive elements (Cs, Sr), or swing adsorption for air-separation processes.

Hydroxysodalite synthesized via the conventional hydrothermal synthesis technique was used as basic solid catalyst to convert waste cooking oil (WCO) to biodiesel [313]. The reaction was conducted at 60 °C for 6 h at a methanol-to-WCO ratio of 7.5:1 and catalyst concentration of 3 wt. %. The results of the analysis of the product revealed that biodiesel was produced. However, no information about the conversion and the biodiesel yield with this catalyst is provided in this article.

Spherical hydroxysodalite particles (HSOD) with a size between 60 and 80 nm were synthesized hydrothermally from Na_2_SiO_3_·5H_2_O and NaAlO_2_ at 80–100 °C and the electrochemical behavior of the modified [Ni–HSOD–chitisan/carbon paste] electrode towards the oxidation of ethanol was evaluated by cyclic voltammograms and chronoamperometry methods [314]. Sodalite and chitosan at the surface of CPE improve catalytic efficiency of the dispersed nickel ions toward oxidation of ethanol and show a good selectivity and stability.

K- and Na-enriched sodalite-type materials with variable morphologies, specific surface areas, basicity, and ion exchange capacity synthesized hydrothermally at 150 °C from thermally activated muscovite and NaOH for 24 to 72 h were used for catalysis of biodiesel production from a 16:1 methanol-to corn oil mixture [315]. The product yield determined after 120 min in the reaction carried out at 70 °C using a catalyst with a high specific surface area and total basicity was 90.5%. The catalyst synthesized during 48 h showed significant regeneration ability and was reused in five cycles, producing valuable biodiesel yields.

A sustainable green catalyst [(Cu_4_Cl)_3_(H_0.5_BTT)_8_(H_2_O)_12_]·3MeOH·9DMF (H_3_BTT = 5,5′-(1,4-phenylene)bis(1H-tetrazole), DMF = dimethyl formamide), which mimics the enzyme oxygen activation is suitable to replace platinum catalysts for the fuel cell [316]. This catalyst reduces oxygen at the onset and half-wave potential of 0.940 V and 0.778 V, respectively. The high oxygen reduction catalytic activity of this compound may be due to the presence of tetrazole ligand and the generation of nascent copper(I).

A sodalite-type porous compound with a metal–organic framework, [(Cu_4_O_0.27_Cl_0.73_)_3_(H_0.5_BTT)_8_(H_2_O)_12_]·3MeOH·DMF (BTT^3−^ = 1,3,5-benzene tristetrazolate, DMF = dimethyl formamide), constructed by square [Cu_4_(μ_4_-O/Cl)] units and triangular BTT ligands can be dehydrated to form [(Cu_4_O_0.27_Cl_0.73_)_3_(H_0.5_BTT)_8_] with coordinatively unsaturated Cu^2+^ centers [317]. The loading of 1 mol% of this catalyst (i.e., as low as one eleventh of that used in related Mn–BTT) leads to as high as 96% conversion of benzaldehyde, indicating that the catalytic activity of M–BTT MOFs was significantly improved via post-modification. In addition, the larger pore volume makes the dehydrated catalyst suitable for selective sorption of N_2_ and O_2_ gases with hysteresis loops over CO_2_ and gaseous H_2_ without hysteresis loops.

Noble-metal-supported catalysts are widely used for hydrogenation performance [318,319,320,321], sodalite-type micro- and mesoporous materials being prospective catalyst supports.

Platinum nanocluster-encapsulated sodalite (PtSOD) with mesoporous structure was successfully synthesized through cation exchange with Pt(NH_3_)_4_Cl_2_ and the catalytic activities of Ni(CH_3_COO)_2_- and Zn(NO_3_)_2_-modified PtSOD-M were evaluated with benzene hydrogenation reactions [322]. During hydroprocessing of benzene, the PtSOD-Ni(CH_3_COO)_2_ catalyst cyclohexane showed improved catalytic activity (with 91.5% conversion) as compared to the Na-form sodalite with microporous structure.

Catalytic hydrogenation activity of Pt-encapsulated sodalite produced by direct hydrothermal synthesis in the presence of Pt(NH_3_)_4_Cl_2_ metal precursor and subsequently performing ion exchange with aqueous H^+^, Na^+^, K^+^, Mg^2+^, Ca^2+^, and Ba^2+^ nitrate solutions was studied in the process of benzene hydrogenation [323]. There is no catalytic hydrogenation activity for Pt/SOD-Na and Pt/SOD-K alone, whereas Pt/SOD-H and Pt/SOD ion exchanged with Mg^2+^, Ca^2+^, and Ba^2+^ show superior catalytic hydrogenation performance without mixing with spillover hydrogen receptor. The catalytic activity was preserved after H_2_S poisoning.

A sulfur-tolerant Pt/Al_2_O_3_ sodalite catalyst with core-shell structure prepared by coating a Pt/Al_2_O_3_ core with a nanosized sodalite shell in combination with a CoMo/Al_2_O_3_ catalyst showed high activity, stability, and high sulfur resistance in the hydrodesulfurization of dibenzothiophene [324]. This system can be used as an active hydrogen emission source, producing active hydrogen and transporting it to the traditional sulfided cobalt–molybdenum catalyst.

Pd^2+^-exchanged mesoporous sodalite and NaA zeolite are suitable as heterogeneous catalysts cross-coupling reactions, such as Suzuki, Heck, and Sonogashira reactions, a versatile route to aryl compounds that are highly useful as pharmaceuticals and fine chemicals [325]. The mesoporous structure with pore diameter >5 nm can allow enhanced diffusion of bulky aryl substrates as compared with solely microporous zeolites. The catalysts were reusable without Pd leaching and agglomeration, as long as the reactions were carried out in air.

A high selectivity of 94.5% was achieved in the semi-hydrogenation of acetylene over sub-1 nm Pd nanoclusters confined within sodalite [326]. The design and utilization of the small-pore zeolite six-membered rings with 2.8 × 2.8 Å in zeolite channels is crucial as it only allows H_2_ diffusion into the channels to reach the encapsulated Pd nanoclusters, and thus avoids over-hydrogenation to form ethane.

Sodalite synthesized by hydrothermal synthetic method from fumed silica, aluminum hydroxide, and sodium hydroxide was employed as a support for holding catalytically active potassium species for stabilization of potassium in combustion of carbonaceous soot matters emitted from diesel engine vehicles [327]. As compared with microporous ZSM-5 zeolite, the ignition temperature of carbon black was lowered by the temperature difference of 100 °C. The catalytic activity enhanced after hydrothermal treatment at 800 °C.

Hydroxysodalite with a specific surface area of 10 m^2^/g, synthesized via a common hydrothermal process in an alkaline medium using coal fly ashes at 100 °C for 24 h, was used as a catalyst for transesterification of soy oil to biodiesel in order to obtain a maximum conversion of 95.5 wt. % at 65 °C with a 4 wt. % catalyst concentration, a 12:1 methanol-to-oil molar ratio, and a reaction time of 2 h [328]. The mechanism of the catalytic transesterification and possible formation of active sites was proposed.

Microporous metal–organic material Zn(5-mtz)(2-eim) with a sodalite-type structure with Zn having tetrahedral coordination and 2-ethylimidazole and 5-methyltetrazole as ligands can be used to entrap 5-fluorouracil exhibiting a high anticancer activity. Before use as a drug delivery carrier, it was activated by solvent exchange with dry ethanol and heated at 80 °C for 24 h [329,330,331].

### 4.6. Electroconductivity

Metallic hydrides of alkaline and rare earths, such as LaH_10_ or YH_10_, form hydrogen-rich sodalite-like clathrates with highly symmetric structures whose critical temperatures (T_c_) are close to room temperature or even higher [101,102,332,333]. The Coulomb screening is rather weak, resulting in a Morel–Anderson pseudopotential μ* = 0.11. The critical temperature for YH_10_ is 310 K at 300 GPa [102].

Among Ca hydrides with different hydrogen contents, the CaH_6_ compound with a body-centered cubic sodalite-type structure (with hydrogen that forms unusual “sodalite” cages containing enclathrated Ca) and superconducting properties is stable at high pressures (above 150 GPa) [334]. The dynamic Jahn–Teller effect enhances electron–phonon coupling and leads to superconductivity of CaH_6_ with the superconducting critical temperature of 220–235 K at 150 GPa, as obtained from the solution of the Eliashberg equations. This is the highest value among all hydrides studied thus far. The stability of this structure is related to the formation of a H_4_ unit.

The partially disordered structure of the semiconducting sodalite-type BaGe_8_As_14_ compound together with a narrow bandgap of 0.43 eV in line with low resistivity of 0.02 Ω·cm, and a high carrier concentration exceeding 10^20^ cm^−3^ at room temperature qualifies this compound as a potential semiconducting thermoelectric material [151].

### 4.7. Magnetic Properties

Two types of ferromagnetic half-metal materials [SOD-(Al,Mn)N and SOD-(Ga,Mn)N with (Al,N)- and (Ga,N)-based frameworks and including Mn regularly substituting Al or Ga] with high Curie temperature, wide half-metallic gap, and large magnetic anisotropy energy in Mn doped III-N low-density cluster-assembled sodalite phases have been predicted based on density functional theory calculations [335]. Due to the strong Mn-N exchange interaction, robust ferromagnetic ground states with high Curie temperatures of up to 788 K and 633 K, respectively, are predicted for these materials. The large half-metal direct band gaps (1.70 eV and 1.33 eV, respectively) make it possible the application of half-metallicity at ambient temperature. The magnetic anisotropy energies are −0.56 and −0.53 meV per Mn atom, respectively, which is two orders of magnitude larger than those of some traditional magnetic materials.

Clusters with the average composition (Rb_2.5_K_1.5_)^3+^ with one unpaired s-electron shared by four alkali cations were introduced in aluminosilicate sodalite obtained by ion-exchange of salt-free Na-sodalite, Na_3_Al_3_Si_3_O_12_, first in a KNO_3_ aqueous solution, and thereafter in a RbNO_3_ aqueous solution at room temperature [336]. Similar antiferromagnetic materials in which Na_4_^3+^, K_4_^3+^, and K_3_Rb^3+^ clusters with an unpaired electron are periodically arranged in a body-centered cubic structure of sodalite have been prepared as well [337,338]. The antiferromagnetism of alkali-metal clusters was investigated using μSR, neutron diffraction, and synchrotron radiation Mössbauer spectroscopy. The magnetic susceptibility and the electron spin resonance of the (Rb_2.5_K_1.5_)^3+^-bearing sodalite show an antiferromagnetic phase transition at a Néel temperature of 90–100 K which is somewhat higher than in analogous materials with K-dominant clusters. This is explained by the systematic change in the size and the spatial distribution of the s-electron wave function in the nanocluster, which may strongly affect the exchange coupling between adjacent nanoclusters. The studied materials do not contain any magnetic elements. Their magnetic order is realized by alkali metal s-electrons. Thus, these data give a new insight into the spatial distribution of the wave function of s-electrons in the nanoclusters, which is responsible for magnetism, unlike the d- and f-electrons in conventional magnets.

According to ^27^Al NMR rata, K,Rb-sodalite with K:Rb = 3:1 has antiferromagnetic transition temperature of 80 K [339]. A monotonous narrow spectrum is seen above the transition temperature and a line-broadened one is observed below 80 K. The formula Rb_2_K_6_(Al_6_Si_6_O_24_) given in [339] for this compound is not charge-balanced: the correct formula may be Rb_2_K_6_(Al_6_Si_6_O_24_)(OH)_2_.

Magnetic properties of alkali-cluster-loaded sodalites have been studied theoretically in frames of an extended Hubbard model for maximally localized Wannier functions. Ab initio screened Coulomb and exchange interactions were calculated by constrained random-phase approximation [340]. It was found that the system resides in the strong-coupling regime, and thus the Heisenberg model is derived as a low-energy model of the extended Hubbard model. The calculated antiferromagnetic couplings are consistent with the experimental temperature dependence of the spin susceptibility.

So-called sodium electrosodalite, i.e., sodalite containing three sodium ions per cage exposed to sodium vapor, undergoes a phase transition to an antiferromagnetic phase when cooled below 48 K, proving that the trapped electrons inside each cage are able to interact over a distance of 7.7 Å [341]. Various alkali-doped electrosodalites form a unique class of magnetic materials where the magnetism comes from non-atom centered unpaired *F*-center electrons found inside body-centered cubic arrangements of alkali ion clusters supported by sodalite host lattices. The crystal structure of potassium electrosodalite has been determined at 20 K using synchrotron powder diffraction [342]. Although potassium electrosodalite has a larger unit cell than sodium electrosodalite, the temperature of magnetic ordering of the former is higher. This phenomenon has been found to be due to both a broadening of the bands belonging to the F-center states and to lower on-site correlations. Non-nuclear maxima are found in the total electron density of both SES and PES and the spin density is found mainly in the center of the sodalite cage. Magnetic properties are sensitive to the size of the *F*-centers. In particular, ^27^Al-NMR measurements demonstrate that sodium electrosodalite and potassium electrosodalite undergo an antiferromagnetic transition at ~48 K and ~70 K, respectively [343].

### 4.8. Thermal Properties and Thermal Conversions

The heat capacity of natural sodalite was measured at temperatures 15–350 and 340–1000 K using adiabatic calorimetry and DSC, respectively [344]. No anomalies were observed except for a peak at 240 K associated with fusion of fluid inclusions. The standard molar entropy is 102.0·*R*. The calculated standard molar enthalpy and standard molar Gibbs free energy of formation of sodalite are −1.6186 × 10^6^·*R*K and −1.5279 × 10^6^·*R*K, respectively.

Heat capacities of sulfate, perrhenate, chloride, and iodide sodalites Na_8_(Al_6_Si_6_O_24_)X_1−2_ (X = SO_4_, ReO_4_, Cl, I) were measured at temperatures from 2 to 300 K [345]. In this temperature interval, heat capacities (J·K^−1^·mol^−1^) determined at constant pressure of 1.2 mPa vary from ~0.030 to ~920 for sulfate sodalite, from ~0.021 to ~990 for chloride sodalite, from ~0.014 to ~930 for iodide sodalite, and from ~0.022 to ~970 for perrhenate sodalite. From the heat capacity data, the standard thermodynamic functions were determined. All four sodalites undergo a phase transition below room temperature for which thermodynamic parameters were determined. It is to be noted that the formula Na_8_(Al_6_Si_6_O_24_)(SO_4_)_2_ given in the cited paper is not charge-balanced: the correct formula should be Na_8_(Al_6_Si_6_O_24_)(SO_4_).

Thermoanalyses of carefully prepared pure phases Na_8_(AI_6_Si_6_O_24_)(OH)_2_·*n*H_2_O (0 ≤ *n* ≤ 4 (“basic series”) and Na_6_(AI_6_Si_6_O_24_)·*n*H_2_O (0 ≤ *n* ≤ 8) (“non-basic series”) reveal an antagonistic volume/concentration effect of hydrate water [34]. Thermogravimetry and X-ray diffraction heating experiments confirm partial collapse of the sodalite framework upon dehydration of phases of the basic hydro-sodalites which is the common behavior of framework compounds in decomposition or ion exchange experiments. In contrast, phases of the non-basic sodalite hydrate series show significant expansion of the aluminosilicate framework, when H_2_O is released at temperatures of 350–450 K under open system conditions.

Sodalite is a high-temperature, low-pressure phase, stable well above the solidus in sodic silica-undersaturated melts enriched in NaCl, and its presence constrains NaCl activities in magmas. The stability of sodalite in the system NaAlSiO_4_–NaCl was studied [346]. The reaction

sodalite → β-nepheline + NaCl

was reversed in solid-medium apparatus in the *P*/*T* ranges 650–900 °C and 7.4–8.6 kbar, and the reaction

sodalite → carnegieite + NaCl

was reversed at 1376–1379 °C and 1 bar.

Potassium carbonate-supported micro- and nanosized sodalite was thermally treated using a muffle-furnace by heating to a temperature of 800 °C at a rate of 10 °C/min and kept at 800 °C for 5 h in a stream of air [347]. According to the X-ray diffraction data, the product of thermolysis is micro- or nanosized nepheline.

**Figure 27:**
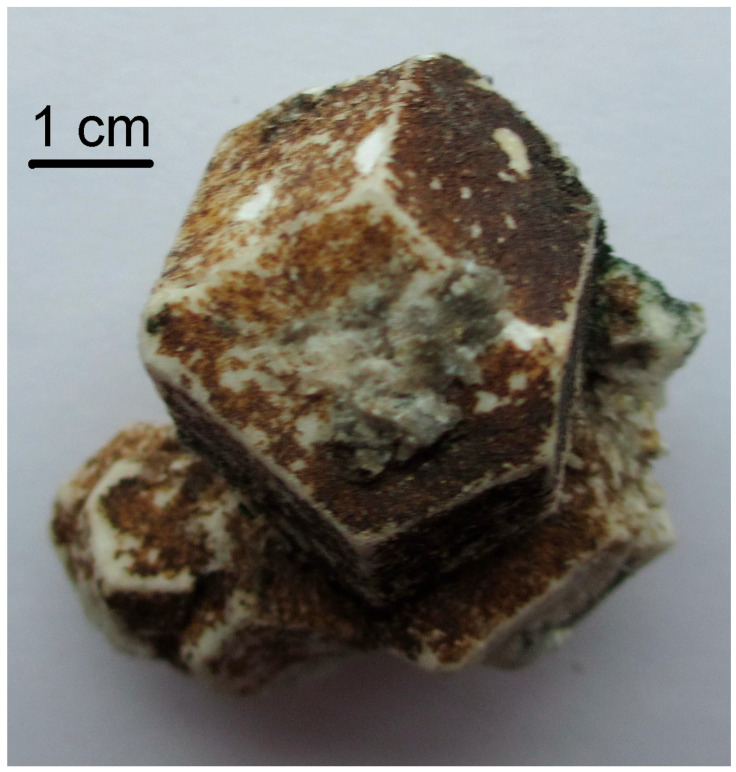
Pseudomorphs of natrolite after sodalite crystals (rhombic dodecahedra) from a hydrothermally altered peralkaline pegmatite at Marchenko Peak, Khibiny alkaline complex, Kola Peninsula.

Sodalite framework is rather stable under low-temperature hydrothermal conditions. However, in natural post-magmatic hydrothermal systems, at temperatures below 250 °C this mineral slowly (over a long period of geological time) transforms to hydrous zeolites (mainly, natrolite, Na_2_(AlSi_3_O_10_)·2H_2_O; rarely, thomsonite-Ca, Ca_2_Na(Al_5_Si_5_O_20_)·6H_2_O, or analcime, Na(AlSi_2_O_6_)·H_2_O), which form partial or complete pseudomorphs after sodalite crystals [348] (Figure 27).

Rietveld refinement of the sodalite structure at temperatures from 28 to 982 °C has shown that the cubic unit-cell parameter for sodalite increases smoothly and non-linearly, and the percent volume change between 28 and 982 °C is 4.8% [349]. In the whole temperature interval, the Al–O and Si–O distances are constant, while the Al–O–Si angle increased from 138.29° to 146.35°. and the angle of rotation of the AlO_4_ tetrahedron decreased from 22.1 to 16.9°. The Na–Cl bond length increased by 0.182 Å. Sodalite melts at about 1079 °C and begins to lose NaCl.

Dehydration of hydroxysodalite proceeds in two steps. Further transformations to α-carnegieite and nepheline take place at 700 °C and 900 °C. These processes were studied by the in situ powder XRD and TG/DTA methods using hydrosodalite samples synthesized hydrothermally at different temperatures (90–140 °C) for 3.5 to 24 h with varying Si/Al ratios in the starting samples [350].

The sodalite-type compounds Sr_8_(Al_12_O_24_)(CrO_4_)_2_ and Sr_8_(Al_12_O_24_)(SO_4_)_2_ show significantly smaller thermal expansion coefficients than aluminosilicate sodalites having similar degrees of structural collapse [351].

Powder X-ray diffraction data of the gallosilicate sodalites Na_8_(Ga_6_Si_6_O_24_)Cl_2_, Na_8_(Ga_6_Si_6_O_24_)Br_2_ and Na_8_(Ga_6_Si_6_O_24_)I_2_ were obtained in the temperature range of 20–900 °C and changes in their crystal structures with temperature were evaluated by Rietveld calculations [352]. Increases of lattice parameters for all three sodalites with increasing temperature were observed and no phase transitions were detected. For the bromide sample, a different nearly linear increase of the lattice parameter was observed up to ~500 °C and above ~600 °C with an invariant behavior between these temperatures. The O–Ga–O angles indicate a disappearance of the tetragonal GaO_4_ tetrahedron distortion around the temperature where this break was observed. The changes of the tilt and Ga–O–Si angle, as well as the interatomic Si–O and Ga–O distances with temperature, were determined.

The temperature dependence of the crystal structure of sodium gallosilicate nitrite sodalite, Na_8_(Ga_12_O_24_)(NO_2_)_2_, was studied between 20 °C and 700 °C from X-ray powder data using the Rietveld method [353]. The linear thermal expansion coefficient was calculated from the lattice expansion data. T–O bond lengths slightly decreased, *T*–O–*T* angle non-linearly increased, tilt of the GaO_4_ tetrahedra decreased, and their tetragonal tetrahedral distortion decreased on heating the polycrystalline sample. In addition, tetragonal tetrahedra distortion of GaO_4_ tetrahedra approached close to zero at higher temperatures. The mobility of sodium atoms above ~330 °C leads to different sodium content in some sodalite cages and results in the appearance of different domains accompanied by drastic changes of the average crystal size, micro-strain. The thermal expansion coefficient remains positive in the whole temperature range. It decreases with temperature below ~330 °C, whereas in the temperature range 330–700 °C, three-fold enhancement of the thermal expansion coefficient takes place.

Rietveld refinement the products of thermal conversions of silica sodalite containing trioxane revealed three steps of the process [354]. At the first step (in the 25–200 °C temperature range), relaxation of the host–guest interactions between trioxane and the framework determines a regularization in the six-membered rings resulting in expansion. The cell volume remains constant until 380 °C, then starts to contract when template molecule decomposition occurs. At the third step, when the trioxane molecule decomposition and expulsion process is completed at above 760 °C, unit-cell volume contraction cannot be fully justified by the negligible mass loss observed on the TG curves, thus suggesting a negative thermal expansion process. The behavior at the third step is unusual for sodalite-type materials, which typically show positive thermal expansion upon heating unless no volatile components are deleted.

According to in situ synchrotron powder diffraction data obtained at temperatures up to 900 °C, expulsion of the trioxane (TR) template from B-bearing silica sodalite Na_0.15_(B_0.07_Si_11.93_O_24_)·1.8TR, synthesized in the presence of boric acid and TR, results in negative thermal expansion above 380 °C [355].

Differential thermal analysis and thermogravimetric analyses of anhydrous natural sodalite-group minerals, sodalite, Na_8_(Si_6_Al_6_O_24_)Cl_2_, tugtupite, Na_8_(Al_2_Be_2_Si_8_O_24_)Cl_2_, danalite, Fe^2+^_8_(Be_6_Si_6_O_24_)S_2_, and helvite, Mn^2+^_8_(Be_6_Si_6_O_24_)S_2_, have been carried out in the temperature range of 20–1450 °C in air [356]. Tugtupite is tetragonal, space group *I*$\bar{4}$; the other studied minerals are cubic, space group *P*$\bar{4}$3*n*. Sodalite melts at 1079 °C and the NaCl component is lost from the melt in two stages (4.5 wt. % at ~1150 °C and 7.0 wt. % at ~1284 °C). Tugtupite melts at 1029 °C and NaCl is also lost in two main stages (1.8 wt. % at about ~1007 °C and 8.2 wt. % in several steps between about 1018 °C and about 1442 °C). Evolution of NaCl from tugtupite directly before its melting may indicate activation of fragmental motion in the crystal or a strong thermal expansion preceding its melting. Such phenomena are quite common and are described in numerous publications [357,358,359,360,361]. Danalite and helvite undergo oxidation of Fe^2+^ to Fe^3+^ and Mn^2+^ to Mn^3+^ and then (about 1300 °C) to Mn^4+^, followed by evolution of sulfur to the gaseous phase and melting at 1060 °C. The maximal oxidation rates are observed at 771 °C for danalite and at 705 °C for helvite.

Iodide sodalite Na_7.7_(Al_6_Si_6_O_24_)_6_I_2_ was examined by temperature-dependent neutron time-of-flight powder diffraction from 5 K to 290 K and by X-ray diffraction from 298 K to 1200 K [362]. The mean structure was refined in space group *P*4$\bar{3}$*n* by Rietveld analysis. A negative slope for the thermal expansion coefficient below 50 K was observed, and the displacement parameters of the iodide ions indicate anharmonic effects. The results of the refinement at very low temperatures indicate a significantly anharmonic potential around I-atoms and isotropic displacements for all other atoms.

Thermal conversions of extra-framework species in sodalite cages are partly discussed above (in Section 3.4). These reactions are accompanied by framework transformations.

Two reversible phase transitions of orthorhombic sodalite-type compound Ca_8_(Al_12_O_24_)(WO_4_)_2_ with the framework totally composed of AlO_4_ tetrahedra occur at 614 and 656 K [363]. Unlike most cubic sodalite-related compounds, thermal transformations of its framework are anisotropic. The anisotropy was attributed to interactions between framework O atoms and WO4 group which is rotated by ~45° about the [001] direction.

The intensity distribution in the MAS NMR signal of the gallosilicate sodalite-related compound Na_6.16_(Ga_1.04_Si_0.96_O_4_)_6_·8H_2_O was modelled to calculate the framework metal second neighbor coordination [364]. The analysis shows a low thermal stability of the cubic sodalite and formation a new intermediate phase which could be regarded as a triclinic distorted cancrinite with three-time increased *c* lattice parameter. Initially, this intermediate phase was described as a compound with an intermediate structure between the structures of sodalite and cancrinite [365,366,367]. Later, the crystal structure of the “intermediate phase” was studied [368]. Its unit-cell parameters are: *a* = 12.6753(9) Å, *c* = 15.526(2) Å; space group *P*3. The framework is characterized by the six-layered cancrinite-related structure with the *CABACB* stacking sequence. The unit cell contains two cancrinite cages, two sodalite cages, and two losod cages. The intermediate phase forms from Na_6.16_(Ga_1.04_Si_0.96_O_4_)_6_·8H_2_O above 600 K and decomposes at around 1000 K to form a beryllonite-type sodium gallosilicate.

Two structural transformations were observed in the cubic sodalite-type compound Na_7.7_(Al_6_Si_6_O_24_)_6_(MnO_4_)_1.7_·0.8H_2_O with *a* = 9.11416 Å [156]. The first one (at 600 K) raises the symmetry from *P*-43*n* to *Pm*-3*n* due to the thermal expansion to a phase with *a* = 9.1819 Å. The second one (at 900 K) lowers the symmetry to *P*23 as a result of thermal decomposition of the MnO_4_^−^ ion. The micro-strain of the sodalite phase decreases up to 900 K, increases with a hump between 900 K and 1020 K, and finally drops to zero.

Cubic Zn-bearing sodalite-related compound Na_6_Zn_2_(Al_6_Si_6_O_24_)(SO_4_)_2_ with the unit-cell parameter of 8.923 Å decomposes above 700 °C, yielding nosean-type Na_8_(Al_6_Si_6_O_24_)(SO_4_) phase, willemite, Zn_2_SiO_4_, gahnite, ZnAl_2_O_4_, and presumably a glass phase with the loss of gaseous SO_3_ [369]. Annealing Na_6_Zn_2_(Al_6_Si_6_O_24_)(SO_4_)_2_ under hydrogen at 700 °C yields sphalerite, ZnS, and a more voluminous sodalite-type phase, Na_6_((Al_6_Si_6_O_24_) with *a* = 9.068 Å. Solid-state NMR indicates that the aluminosilicate framework remains essentially unchanged throughout this reaction, with the largest change observed for ^23^Na.

Temperature-dependent development of the structural parameters of the sodalite-type compound Na_8_(Ga_6_Ge_6_O_24_)(BH_4_)_2_ evaluated by Rietveld X-ray diffraction analysis indicates a beginning oxidation of the enclathrated NaBH_4_ at about 255 °C and a destruction of the sodalite framework starting at about 375 °C [370]. Temperature-dependent Raman and FTIR data confirm these findings.

Thermogravimetry, differential thermogravimetry, and differential thermoanalysis, coupled with mass spectrometry, of the cubic sodalite-type compound Na_8_(AlSiO_4_)_6_[B(OH)_4_]_2_ with *a* = 9.010 Å shows a significant expansion of the framework at elevated temperatures of 573–825 K, when a total of four molecules of H_2_O are released in a two-step decomposition reaction, in accordance with the bulk scheme:

Na_8_(AlSiO_4_)_6_[B(OH)_4_]_2_ → 6NaAlSiO_4_ + 2NaBO_2_ + 4H_2_O(gas),

in accordance with [371]. The resulting phase is orthorhombic, with the unit-cell parameters *a* = 25.510, *b* = 12.750, and *c* = 9.020 Å. However, thermal expansion indicates that borate groups occur in the structure of the final product whose correct formula should be Na_8_(AlSiO_4_)_6_(BO_4_)_2_.

The framework expansion of the sodalite-type Na_8_(Al_6_Si_6_O_24_)(CO_3_) compound was observed starting from room temperature, whereas a decrease in the unit-cell volume of presumed hydrous basic carbonate sodalite Na_8_(Al_6_Si_6_O_24_)(OH)(CO_3_)_0.5_·3H_2_O (synthesized hydrothermally at 80 °C) at elevated temperatures was a result of two-step dehydration followed by decomposition of carbonate groups, which completes at 700 °C [372]. However, direct introduction of carbonate groups in hydrous basic sodalite under mild hydrothermal conditions is questionable. This assumption is confirmed by the IR spectrum given in [372], which contains a rather broad band of C–O stretching vibrations which may be related to an admixed carbonate phase, as well as the absence of stabilizing effect of the sodalite framework. Carbonate groups of Na_8_(Al_6_Si_6_O_24_)(CO_3_) decompose at 850 °C. This process is accompanied by the total destruction of the sodalite framework and the formation of a “stuffed” carnegieite, which transforms into nepheline at higher temperatures.

High temperature X-ray powder diffraction studies on the sodalite-type compound Na_8−*x*_Ag*_x_*(Al_6_Si_6_O_24_)Z_2_ (0 ≤ *x* ≤ 8; Z = Cl, Br, I) show that they display positive thermal expansion from room temperature up to 822 °C [33]. No significant expansion of the M–Z or M–O distances was observed in the whole temperature range. Silver doping results in a decrease of the thermal expansion coefficients of bromide members of this solid–solution system, but no clear trends were found for the chloride members.

Low-temperature thermal properties of silica sodalites with enclosed ethylene glycol, ethanolamine, and ethylenediamine were investigated by differential scanning calorimetry and temperature dependent powder X-ray diffractometry [373]. Heating from 100 K to 300 K results in the transformation of initially low-symmetry (monoclinic or tetragonal) structures to cubic ones. Minimal changes of the guest component, e.g., substitution of hydroxy against amino groups, can lead to drastic changes in the host–guest interactions.

Stability of hydrous hydroxysodalite in high ionic strength solutions at 25–100° has been evaluated by applying a high temperature Al–Si Pitzer model [374]. It was shown that in brines characteristic of salt formations, the solubility products of hydrous hydroxysodalite are very low (~10^−13^ mol/kg at 100 °C). The equilibrium constants obtained in this study may have a wide range of applications, including synthesis of hydroxysodalite, desilication in the Bayer process for extraction of alumina, and the performance of proposed sodalite waste forms in geological repositories.

In situ high-temperature single-crystal X-ray diffraction investigation of modulated haüyne was studied in the temperature range 20–1000 °C [375]. The linearity of its thermal expansion is lost between 600 and 700 °C, and satellite peaks disappeared at 700 °C. This structural change has been attributed to possible rearrangement of the Al/Si distribution, but it could be also due to partial transformation of extra-framework sulfate groups to S_3_^•−^ [168].

Two kinetically different thermally activated processes proceed during heating 3D modulated cubic lazurite up to 750 °C: framework expansion due to Si-O-Al angle increase and equalizing of periodic local distortions via the diffusion-controlled transfer of cage ions between adjacent sub-cells [149]. The latter process is much slower than the first one, especially at lower temperatures. High-temperature transformations are accompanied by the disappearance of satellite reflections and are irreversible, which may be due to chemical conversions of S-bearing extra-framework species (see Section 3.4).

Bicchulite, Ca_8_(Al_8_Si_4_O_24_)(OH)_8_, decomposes with the formation of gehlenite, Ca_2_Al(SiAl)O_7_, and H_2_O above 640 °C. Below this temperature, its thermal expansion coefficient is positive [376].

As a rule, evolution of volatile extra-framework components and products of their decomposition from sodalite cages results in the decrease of the init-cell parameter. Hydrosodalite, Na_6_(Al_6_Si_6_O_24_)·8H_2_O [32,364,377,378,379,380] is an exception of this rule. In this compound, H_2_O molecules form (H_2_O)_4_ tetrahedra and form additional hydrogen bonds with O atoms of the framework which results in contracting of sodalite cages. Thermal dehydration of Na_6_(Al_6_Si_6_O_24_)·8H_2_O at 402 °C results in the formation of the sodalite-type compound Na_6_(Al_6_Si_6_O_24_) and increase of the unit-cell parameter from 8.848 (at 22 °C) to 9.122 Å (at 402 °C) which only partly could be explained by thermal expansion. This transformation is accompanied by the enhancement of the Al–O–Si angle.

Nosean, Na_8_(Al_6_Si_6_O_24_)(SO_4_)·H_2_O, loses water in the temperature range 250–500 °C. Heating of nosean from room temperature to 1200 °C results in an insignificant reduction of its unit-cell parameter from 9.092 to 9.085 Å [135].

### 4.9. Vibrational Spectroscopy of Sodalite-Group Minerals

Minerals belonging to the sodalite group are listed in Table 6. They have aluminosilicate (with the Al:Si ratios of 1:5, 1:1 or 2:1), beryllosilicate (with Be:Si = 1:1), beryllo-aluminosilicate (with Be:Al:Si = 1:1:4), or ferrite frameworks belonging to the sodalite topological type. Sodalite-group minerals are cubic or pseudo-cubic with tetragonal, orthorhombic, monoclinic, or triclinic framework distortions.

Aluminosilicate members of the sodalite group with Al:Si = 1:1 are most common. In these minerals, Al and Si are ordered and wavenumbers of lattice modes involving out-of-phase vibrations of the Si–O–Al fragments are in the range 900–1100 cm^−1^. Corresponding bands are strong in the IR spectra and weak in the Raman spectra [17,18,20,21,22,23,24,25,26,27,381]. The maxima of IR absorption bands of out-of-phase vibrations of the Al–O–Al and Si–O–Si fragments (in bicchulite and tsaregorodtsevite) are observed at 854 and 1091 cm^−1^, respectively [382,383]. The ranges 500–800 and 350–500 cm^−1^ correspond to O–*T*–O and *T*–O–*T* bending modes, respectively (*T* = Si, Al).

The wavenumbers of bending vibrations of the [Be_6_Si_6_O_24_] framework are in the range 510–560 cm^−1^. Be–O stretching modes of sodalite-group minerals with Be-bearing frameworks are observed in the IR spectra in the range 700–790 cm^−1^ [382].

**Figure 28:**
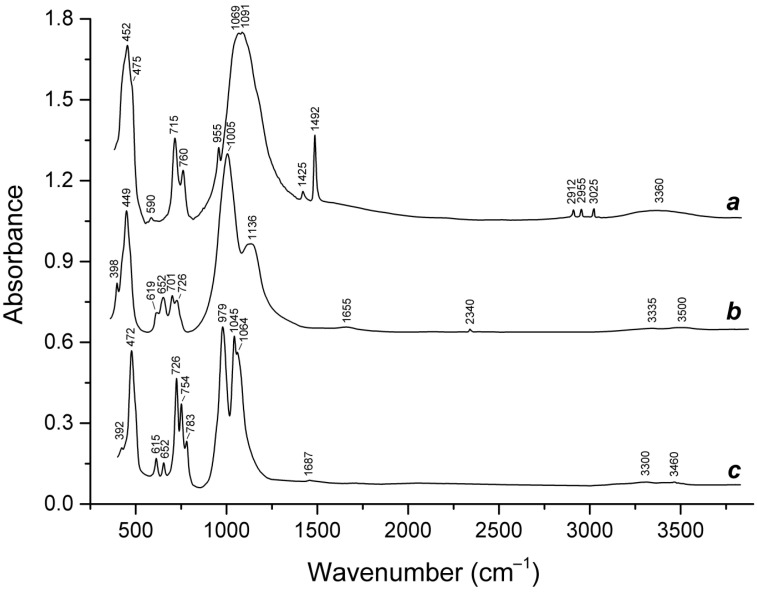
Representative IR spectra of (**a**) H_2_O-bearing tsaregorodtsevite, [N(CH_3_)_4_]_2−x_(Al_2−*x*_Si_10+*x*_O_24_)·*n*H_2_O, (**b**) H_3_O^+^-, H_2_O-, and CO_2_-bearing haüyne, (Na,K,H_3_O)_6_Ca_2−*x*_(Al_6_Si_6_O_24_)(SO_4_)_2−*x*_(CO_2_)*_y_*(H_2_O)_z_ (*x*, *y*, *z* << 1), and (**c**) H_3_O^+^- and H_2_O-bearing tugtupite, (Na,H_3_O)_8−*x*_(Al_2_Be_2_Si_8_O_24_)Cl_2−x_H_2_O)_y_ (*x*, *y* << 1). The spectra are offset for comparison.

**Figure 29:**
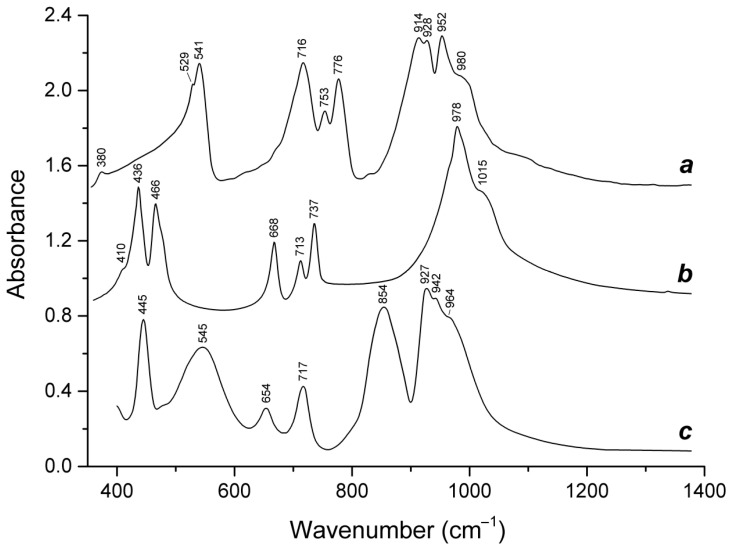
Representative IR spectra of (**a**) Mn-bearing genthelvite, (Zn,Mn)_8_(Be_6_Si_6_O_24_)S_2_, (**b**) sodalite, Na_8_(Si_6_Al_6_O_24_)Cl_2_, and (**c**) bicchuite, Ca_8_(Al_8_Si_4_O_24_)(OH)_8_. The spectra are offset for comparison.

**Figure 30:**
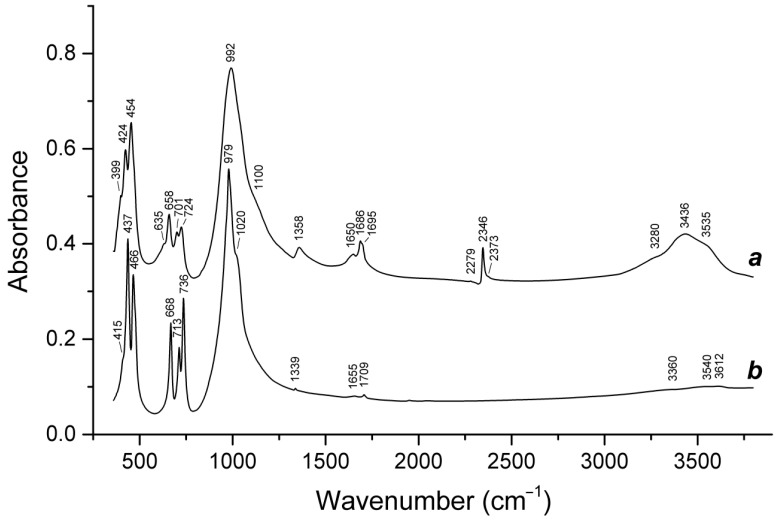
Representative IR spectra of (**a**) CO_2_-rich and H^+^- and HS^−^-bearing bolotinaite, H_0.17_Na_5.92_K_0.82_Ca_0.10_(Si_6.33_Al_5.67_O_24_)(SO_4_)_0.17_F_0.84_Cl_0.16_(H_2_O)_3.36_(CO_2_)_0.38_ and (**b**) sodalite containing minor H^+^ and H_3_O^+^.

**Figure 31:**
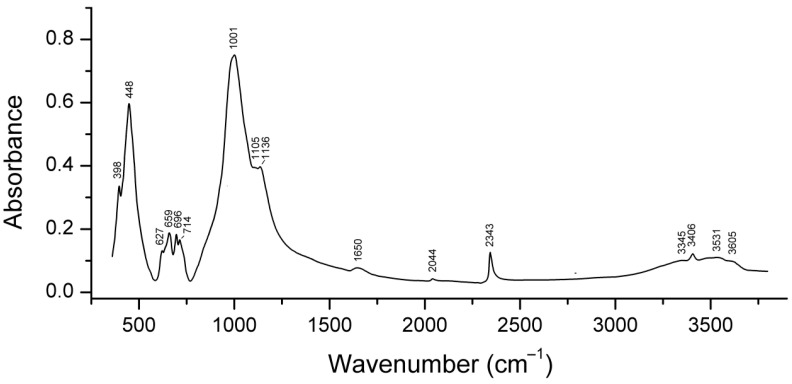
IR spectrum of a CO_2_-rich lazurite-related mineral with the empirical formula (Na_7.43_K_0.16_Ca_0.43_)(Si_6.17_Al_5.75_Fe^3+^_0.08_O_24_)(S^2−^,SO_4_^2−^)_1.21_(S_3_^−^)_0.15_Cl_0.06_(CO_2_)_0.46_(COS)***_x_***·*n*H_2_O (*x* << 1, *n*~1) [23]. The band at 2044 cm^−1^ corresponds to a minor admixture of COS molecules.

**Figure 32:**
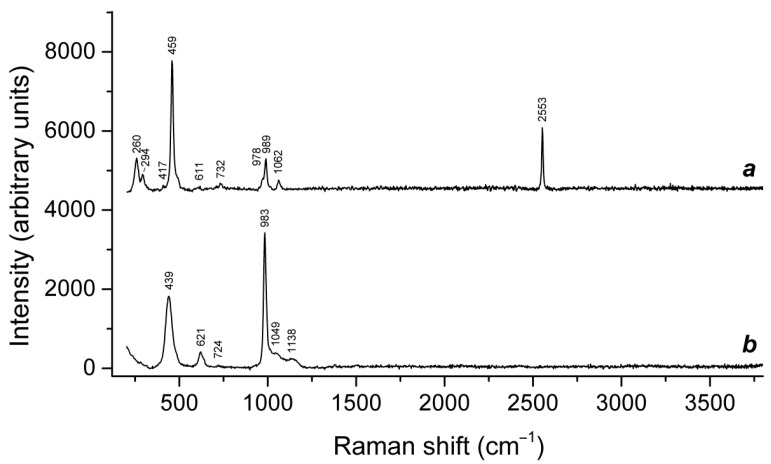
Baseline-corrected Raman spectra of (**a**) Cl-bearing sapozhnikovite, Na_8_(Si_6_Al_6_O_24_)(HS,Cl)_2_, and (**b**) anhydrous nosean analogue, Na_8_(Al_6_Si_6_O_24_)(SO_4_).

**Figure 33:**
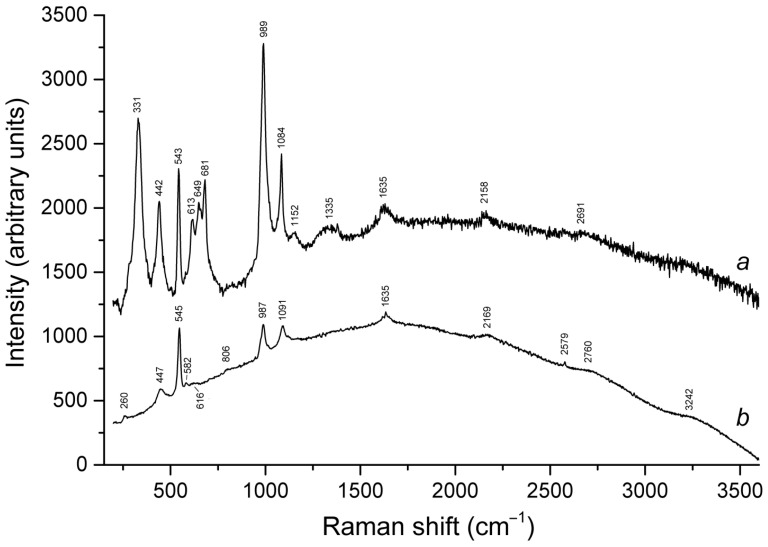
Uncorrected Raman spectra of (**a**) S_4_- and S_2_^•−^-rich and S_3_^•−^-bearing haüyne, (Na,K)_6_Ca_2−*x*_(Al_6_Si_6_O_24_)(SO_4_^2−^,S_4_,S_2_^•−^,S_3_^•−^)_2_, and (**b**) SO_3_^2−^- and S_2_^•−^-bearing haüyne, (Na,K)_6_Ca_2−*x*_(Al_6_Si_6_O_24_)(SO_4_^2−^, SO_4_^2−^,S_3_^•−^,S_2_^•−^)_2_. The luminescence is caused by the presence of the S_2_^•−^ radical anion.

**Figure 34:**
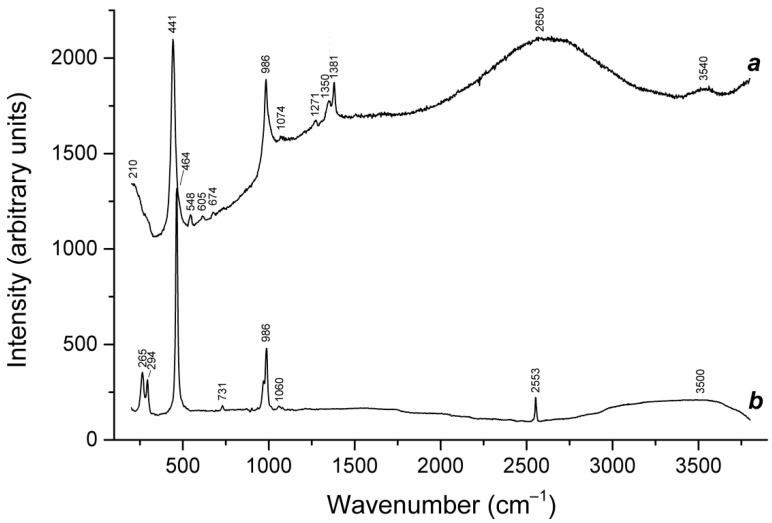
Uncorrected Raman spectra of (**a**) CO_2_-rich and H^+^- and S_2_^•−^-bearing bolotinaite and (**b**) HS^−^-bearing sodalite. The luminescence peaks with the maxima about 2650 and 3500 cm^−1^ are caused by S_2_^•−^ and ^IV^Fe^3+^, respectively.

**Figure 35:**
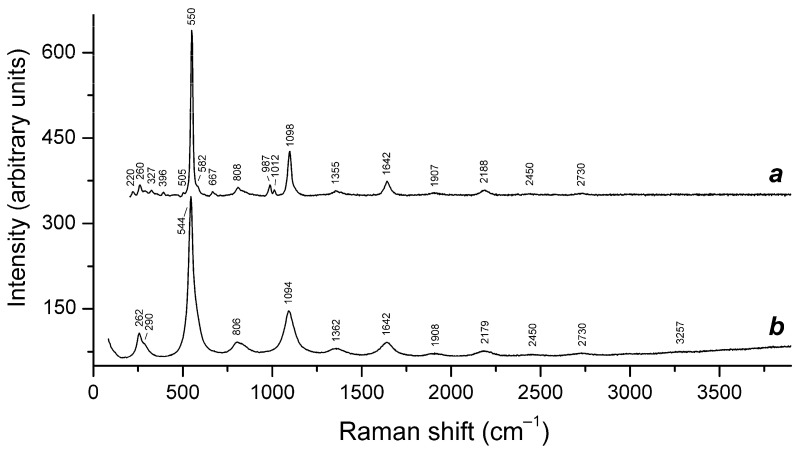
Baseline-corrected Raman spectra of (**a**) S_3_^•−^-bearing haüyne, (Na,K)_6_Ca_2−*x*_(Al_6_Si_6_O_24_)(SO_4_^2−^,S_3_^•−^)_2_, and (**b**) lazurite neotype with the empirical formula (Na_6.97_Ca_0.88_K_0.10_)_∑7.96_[(Al_5.96_Si_6.04_)_∑12_O_24_](SO_4_^2−^)_1.09_(S_3_^•−^)_0.55_S^2−^_0.05_Cl_0.04_·0.72H_2_O.

Representative IR and Raman spectra of minerals belonging to the sodalite group are given in Figure 28, Figure 29, Figure 30, Figure 31, Figure 32, Figure 33, Figure 34 and Figure 35. The assignment of bands of extra-framework anions, radical anions, and neutral molecules in the IR and Raman spectra of sodalite-group minerals is given in Table 7 and Table 8.

As one can see from Table 8, the major extra-framework anions and neutral molecules in sodalite-group minerals are rather diverse. Some other non-cationic components (SO_3_^2−^, S^2−^, MoO_4_^2−^, WO_4_^2−^, AsO_4_^3−^, S_2_^•−^, *cis*- and *trans*-S_4_^•−^, CO_2_, and COS) occur in sodalite cages of some sodalite-group minerals in subordinate amounts [7,17,18,20,21,22,23,24,25,26,27,28]. IR spectroscopy is sensitive to O-bearing extra-framework species, whereas Raman spectroscopy is more sensitive to polysulfide groups and HS^−^ anions whose IR bands are very weak.

**Figure 36:**
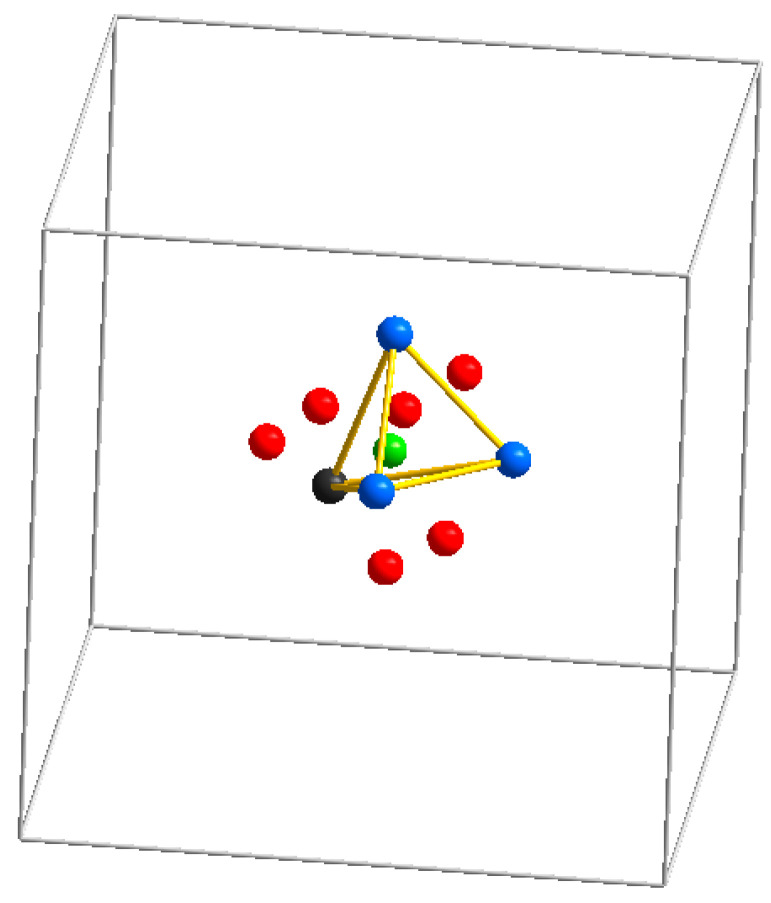
The arrangement of different sites of water molecules (red, blue and black balls) in H^+^-bearing bolotinaite. The site shown as the green ball may be occupied by F with minor admixtures of Cl and S (in the presence of H_2_O molecules at the sites shown as red balls) or by H^+^ (in the presence of water molecules forming the tetrahedron). The unit cell is outlined.

It is to be noted that protons in H^+^-bearing bolotinaite having the empirical formula H_0.17_Na_5.92_K_0.82_Ca_0.10_(Si_6.33_Al_5.67_O_24_)(SO_4_)_0.17_F_0.84_Cl_0.16_(H_2_O)_3.36_(CO_2_)_0.38_ [21] may occur as a part of the Eigen cation, H_9_O_4_^+^. Indeed, a part of H_2_O molecules in bolotinaite occur as tetrahedra with two short O···O distances (2.44 ± 0.04 Å) and two longer O···O distances (2.93 ± 0.04 Å) (Figure 36). Short O···O distances (below 2.6 Å) are typical for hydrated proton complexes, including Eigen cation. According to the correlations ν (cm^−1^) = 152·10^9^·exp[–*d*(O···O)/0.1321] + 2315*d*(O···O) − 2859 [385] and ν (cm^−1^) = 3592 − 304·10^9^·exp[–*d*(O···O)/0.1321] [386], the wavenumber of the band observed in the IR spectra of bolotinaite at 1358 cm^−1^ corresponds to the O···O distance of 2.39 and 2.48 Å, respectively, in agreement with the structural data. The O···O distance corresponding to the largest wavenumber of O–H stretching vibrations in the IR spectrum of bolotinaite (3535 cm^−1^) calculated using the latter correlation is equal to 2.96 Å.

## 5. Conclusions

The sodalite topological type is the most widespread among microporous materials and is realized in compounds of a wide variety of chemical compositions, including those with element–oxygen, halide, and metal–organic frameworks, and a wide range of extra-framework cations, anions, radical anions, and neutral molecules. This indicates a wide range of conditions of thermodynamic stability of these materials. As a consequence, most sodalite-related materials are characterized by high thermal stability. This fact, along with a microporous structure with a system of intersecting channels and a high degree of elasticity of the framework, allows us to consider SOD-type compounds as promising materials with useful ion-exchange, immobilization, ion-conducting, and catalytic properties, and matrices for hydrogen storage and stabilization of various particles, including chromophores and phosphors.

Significant reserves of cheap raw materials that can be used for the synthesis of sodalite-related microporous materials are available. Among them, there are various waste products (fly ash, furnace slag, rice husk ash, etc.)

Sodalite group minerals are among the main components of some rocks. Extra-framework components in these minerals are important geochemical markers that make it possible to reconstruct the conditions of mineral formation.

## Figures and Tables

**Table 1:** Mean *T*–O–*T* angles, the ranges *T*–O–*T* angles of individual (ind) SOD-type compounds, the number of observations (*N*) for chemically different samples, and the mean *T*–O distances for all studied samples [76].

Angle	*T*–O–*T*, Mean [°]	Range of *T*–*X*–*T*, Ind [°]	*N*	*T*–O, Mean [Å], All Samples
Al–O–P	no data	no data	no data	1.63
Si–O–Si	150.6	141–160	4	1.60
Al–O–Al	144.3	128–166	16	1.74
Al–O–Si	142.0	135–156	32	1.67
Al–O-Ge	133.9	130–138	7	1.74
Ge–O–Ge	129.3	125–134	2	1.74
Ga–O–Si	130.2	130	2	1.72
Be–O–Si	129.9	123–144	9	1.61
Ga–O–Ge	129.7	125–137	5	1.79
B–O–B	124.7	120–128	5	1.47

**Table 2:** Unit-cell parameters of synthetic aluminosilicate sodalites with different anionic groups [21,129,130,131,132].

Extra-Framework Anions and Groups	*r*, Å	*a*, Å	*V*, Å^3^
(OH)^−^	1.32	8.885	701.4
Cl–	1.81	8.873	698.6
Br–	1.96	8.932	712.6
(CO_3_)^2−^	2.57	9.005	730.2
(NO_3_)^−^	2.60	8.997	728.3
(SO_4_)^2−^	2.98	9.072	746.6
(WO_4_)^2−^	3.40	9.148	765.5
(MoO_4_)^2−^	2.00	9.152	766.5
(ReO_4_)^2−^	2.60	9.153	766.8
(AlF_6_)^3−^+H_2_O	2.56	9.046	749.3
N_3_^−^	1.95	8.982	724.5
(H_2_O)_4_	2.90	9.034	737.2

**Table 3:** Powder X-ray diffraction data of S_3_^•−^-bearing haüyne from the Malo-Bystrinskoe deposit, Baikal Lake area [27].

*h*, *k*, *l* *	*d*_meas_ (Å)	*d*_calc_ (Å)	*I* (%)	*h*, *k*, *l* *	*d*_meas_ (Å)	*d*_calc_ (Å)	*I* (%)
1 1 0	6.42	6.41	13.5	3 + *n*, 3, *n*	2.060	2.060	3.8
1 + *n*, 1, *n*	5.72	5.71	4.4	4 + *n*, 1 + *n*, 1	2.015	2.016	2.5
2 − 0.5, 1 − 0.5, 1	4.859	4.847	3.4	3 3 2	1.9328	1.9331	3.3
2 0 0	4.538	4.534	5.8	4 2 2	1.8487	1.8508	2.2
2 − *n*, 1 − *n*, 1	4.139	4.134	4.5	4 + *n*, 2 − *n*, 2	1.8144	1.8154	1.9
2 1 0	4.058	4.055	3.6	5 1 0, 4, 3, 1	1.7781	1.7782	7.6
2, 1 − *n*, 1 − *n*	3.961	3.962	4.2	4, 3 + *n*, 1 − *n*	1.7482	1.7466	1.8
2 − 0.5, 1 + 0.5, 1	3.862	3.866	2.9	5 + *n*, 1 − *n*, 0	1.7188	1.7197	1.6
2 1 1	3.703	3.702	100	5 2 1	1.6553	1.6554	2.8
2 + *n*, 1 − *n*, 1	3.551	3.551	3	4 − *n*, 4, *n*	1.6446	1.6449	2
2, 1 + *n*, 1 + *n*	3.440	3.441	4.5	4 − *n*, 3 − *n*, 3	1.6253	1.6258	1.6
2+ *n*, 1 + *n*,1	3.339	3.339	4.3	4 4 0	1.6028	1.6029	7.2
2 2 0	3.203	3.206	3.2	4 − *n*, 3 + *n*, 3	1.5551	1.5596	3.8
2 − 0.5, 2 + 0.5, 0	3.111	3.110	2.9	4 4 2	1.5109	1.5112	3.5
2 + 0.5, 1 + 0.5, 1	2.943	2.942	2.9	6 1 1	1.4710	1.4709	4.3
3 1 0	2.868	2.867	13.9	4 + *n*, 4, 2 + *n*	1.4586	1.4584	1.7
2, 2 − *n*, 2 − *n*	2.816	2.814	4.2	5, 4 − *n*, 1 + *n*	1.4199	1.4193	1.7
3 + *n*, 1 − *n*, 0	2.732	2.740	2.7	5 4 1	1.3988	1.3991	1.6
2 2 2	2.618	2.617	25.9	6 − *n*, 2, 2 + *n*	1.3926	1.3927	1.6
2 + *n*, 2 + *n*, 2	2.441	2.441	3.8	6 2 2	1.3669	1.3669	5.4
3 2 1	2.423	2.423	7.9	6 3 1	1.3371	1.3369	2.2
4 0 0	2.267	2.267	6.9	4 4 4	1.3085	1.3087	3.5
4 − *n*, 1 + *n*, 1	2.211	2.211	3.3	7 1 0	1.2825	1.2823	2.2
4, 1 − *n*, 1 − *n*	2.183	2.184	2.6	7, 2 − *n*, 1 − *n*	1.2479	1.2477	1.4
4 1 1	2.137	2.137	16.1	7 2 1	1.2339	1.2339	3.7
3 + 2*n*, 2 + 2*n*, 1	2.092	2.100	2.7	7, 2 + *n*, 1 + *n*	1.2184	1.2185	1.4

* Simple symbols (1 1 0 etc.) denote the indices of basic reflections; symbols containing ±0.5 (2 − 0.5, 1 − 0.5, 1 etc.) refer to the powder diffraction lines of commensurate superstructure; symbols (*h* ± *n*, *k* ± *n*, *l*), containing *n* (the incommensurate modulation parameter) refer to the lines of incommensurate superstructure (satellite reflections). Note: the cubic sub-cell parameter *a* = 9.067(2) Å, *n* = 0.214.

**Table 4:** Data on selected LRM with modulated structures [28].

Mineral	Parameters of the Basic Unit Cells *a*. *b c* (Å) α, β, γ (°)	Symmetry Group	Wave Vector
Cubic LRM-1	*a_cub_* = 9.087(3)	*P*23	**q** ~ 0.30c* (in orthorhombic setting)
Cubic LRM-2	*a_cub_* = 9.077(1)	*P*23	**q** ~ 0.43c* (in orthorhombic setting)
Orthorhombic LRM *a*~*a*_cub_, *b*~*a*_cub_√2 *c*~3*a*_cub_√2	*a* = 9.057 *b* = 12.843 *c* = 38.513	*Pnaa*	**q** = 0.33c*
Monoclinic LRM *a*~*a*_cub_, *b*~*c*~*a*_cub_√2	*a* = 9.069(1) *b* = 12.868(1) *c* = 12.872(1) γ = 90.19(1)	*P*11*a*(00δ)0	**q** ~ 0.43c*
Triclinic LRM *a*~*a*_cub_, *b*~*a*_cub_√2 *c*~2*a*_cub_√2	*a* = 9.0523(4) *b* = 12.8806(6) *c* = 25.681(1) α = 89.988(2) β = 90.052(1) γ = 90.221(1) (*T* = 170 K)	*P*1	**q** = 0.5c*

Note: In triclinic LRM, modulation is due to the alternation of SO_4_^2−^ and S_6_. In other samples SO_4_^2−^ alternates with S_3_^•−^.

**Table 5:** Assignment of Raman bands of slyudyankaite and products of its thermal conversions.

Raman Shift (cm^−1^)			Assignment
Initial Sample	Preheated Sample	Sample Heated at 800 °C in Air	
219			*trans*-S_4_ bending
260		262	S_3_^•−^ bending A_2_ (ν_2_) and S_6_ (with D_3d_ symmetry) bending
283			Framework bending vibrations (resonance with a S_6_ bending mode?)
298	302s		S_4_^•−^ bending vibrations
330			*cis*-S_4_ mixed ν_4_ mode (symmetric bending + stretching)
380 w			*cis*-S_4_ mixed ν_3_ mode
437		440w	SO_4_ [bending E (ν_2_) mode] and/or S_6_ (mixed mode)
	461s		[(HS)Na_4_]^3+^ stretching vibrations
477		474	S_6_ stretching mode and/or mixed ν_4_ mode of *trans*-S_4_
503			Bending vibrations of the framework
545 s		548s	S_3_^•−^ symmetric stretching (ν_1_) (possibly, overlapping with the stretching band of *gauche*-S_4_)
580		586	S_3_^•−^ antisymmetric stretching mode (ν_3_)
	603		S_2_^•−^ stretching mode
614			SO_4_^2−^ [bending F_2_ (ν_4_) mode]
645			*cis*-S_4_ stretching
682			*trans*-S_4_ symmetric stretching ν_3_ mode
	724, 756w	722w	O–C–O bending vibrations of oxalate anions
807		810	S_3_^•−^ combination mode (ν_1_ + ν_2_)
985 s	983		SO_4_^2−^ [symmetric stretching A_1_ (ν_1_) mode] (possibly, overlapping with the weak band of framework stretching vibrations)
	1077w		CO_3_^2−^ symmetric stretching mode
1088 s		1097s	S_3_^•−^ overtone (2 × ν_1_) [possibly, overlapping with the SO_4_^•−^ stretching band (ν_3−_F_2_)]
	1163		S_2_^•−^ overtone (2 × ν_1_)
1279, 1381			Symmetric stretching vibrations of CO_2_ molecules (Fermi doublet, resonance with the overtone of bending vibrations).
1340	1342	1360	Symmetric C–O stretching vibrations of CO_2_ molecules—involved in strong dipole–dipole interactions and/or symmetric C–O stretching vibrations of acid oxalate anions
	1480w		CO_3_^2−^ asymmetric stretching mode
	1609s		Antisymmetric C–O stretching vibrations of acid oxalate anions
1631		1651	S_3_^•−^ overtone (3 × ν_1_)
	1768, 1832		C = O stretching vibrations of acid oxalate groups
1891		1915	S_3_^•−^ combination mode (3 × ν_1_ + ν_2_)
2172		2191	S_3_^•−^ overtone (4 × ν_1_)
2428 w			S_3_^•−^ combination mode (4 × ν_2_ + ν_1_)
	2553s		HS^−^ stretching mode
2575 w			H_2_S symmetric stretching mode
2710		2737	S_3_^•−^ overtone (5 × ν_1_)
2964 w			S_3_^•−^ combination mode (5 × ν_1_ + ν_2_)
3025			O–H stretching vibrations
3247 w			S_3_^•−^ overtone (6 × ν_1_)

**Table 6:** Sodalite-group minerals.

Name	Simplified Formula	Sp. Gr.	Unit-Cell Parameters, Å	Ref.
Sodalite	Na_8_(Si_6_Al_6_O_24_)Cl_2_	*P-*43*n*	*a* = 8.87–8.88	[2]
Sapozhnikovite	Na_8_(Si_6_Al_6_O_24_)(HS)_2_	*P-*43*n*	*a* = 8.9146	[17]
Bolotinaite	(Na_6_K□)(Al_6_Si_6_O_24_)F·4H_2_O	*I-*43*m*	*a* = 9.027	[21]
Haüyne	(Na,K)_6_Ca_2_(Al_6_Si_6_O_24_)(SO_4_)_2_	*P-*43*n*	*a* = 9.09–9.13	[4]
Lazurite	Na_7_Ca(Al_6_Si_6_O_24_)(SO_4_)S_3_^•−^·H_2_O	*P-*43*n* (for the sub-cell)	*a* = 9.08 (for the sub-cell) The structure is modulated	[7,20]
Lazurite-related mineral	Na_8_(Al_6_Si_6_O_24_)(S^2−^,SO_4_^2−^,S_3_^•−^)_1+*x*_ (H_2_O,CO_2_)	No data	No data	[23]
Nosean	Na_8_(Al_6_Si_6_O_24_)(SO_4_)·H_2_O	*P-*43*n*	*a* = 9.05–9.08	[8]
Bicchulite	Ca_8_(Al_8_Si_4_O_24_)(OH)_8_	*I-*43*m*	*a* = 8.82–8.83	[9]
Kamaishilite	Ca_8_(Al_8_Si_4_O_24_)(OH)_8_	No data (tetragonal)	*a* = 8.850, *c* = 8.770	[10]
Valleyite	Ca_8_(Fe_12_O_24_)O_2_	*I-*43*m*	*a* = 8.8852	[11]
Vladimirivanovite	Na_6+*x*_Ca_2–*x*_(Al_6_Si_6_O_24_) (SO_4_,S_3_^•−^)_2_·H_2_O	*Pnaa*	*a* = 9.053, *b* = 12.837, *c* = 38.445	[12]

**Table 7:** Assignment of absorption bands of extra-framework components in IR spectra of sodalite-group minerals and related feldspathoids [7,17,18,20,21,22,23,24,25,26,27,28,265,268,269,277,278,384].

Wavenumber (cm^−1^)	Assignment
448–454	S_5_^2−^ stretching mode 2
530	Stretching vibrations of *trans*-S_4_
580–585	S_3_^•−^ antisymmetric stretching mode (ν_3_)
614–622	SO_4_^2−^ bending vibrations [*F*_2_(ν_4_) mode]
955	C–N stretching mode of N(CH_3_)_4_^+^
1130–1160	SO_4_^2−^ asymmetric stretching vibrations [*F*_2_(ν_3_) mode]
1339–1358	H···O stretching vibrations of hydrated proton complexes
1400–1500	CH_3_ bending modes of N(CH_3_)_4_^+^
1610–1660	H_2_O bending vibrations
1685–1720	H_3_O^+^ bending vibrations
2037–2040	C=O stretching vibrations of COS
2340–2343	Antisymmetric stretching mode of CO_2_
2554	Stretching vibrations of HS^−^
2900–3030	CH_3_ stretching modes of N(CH_3_)_4_^+^
3300–3600	H_2_O stretching vibrations
3600–3670	Stretching vibrations of OH^−^

**Table 8:** Assignment of Raman bands of extra-framework components in sodalite-group minerals and related feldspathoids [7,17,18,20,21,22,23,24,25,26,27,28,265,268,269,277,278,384].

Raman Shift (cm^−1^)	Assignment
219–223	*trans*-S_4_ or S_4_^2−^ bending mode
254–25	S_3_^•−^ bending mode (ν_2_)
254–260	Bending vibrations of the [ClNa_4_] and [(HS)Na_4_] clusters
262	S_5_^2−^ stretching mode?
283–294w	Combination of low–frequency lattice modes involving Na^+^ cations and S_6_ bending mode
298	S_4_^•−^ bending vibrations
327–332w	*cis*-S_4_ mixed ν_4_ mode (combined symmetric bending + stretching vibrations)
380	*cis*-S_4_ mixed ν_3_ mode
413–422	S_5_^2−^ stretching mode 1
435	S_5_^2−^ stretching mode 1 (a different conformation)
436–447	SO_4_^2−^ [the *E*(ν_2_) mode]
454–466	S_5_^2−^ stretching mode 2
459–464	Stretching vibrations of the [ClNa_4_] and [(HS)Na_4_] clusters
477–481	S_6_ stretching mode and/or mixed ν_4_ mode of *trans*–S_4_ or S_4_^2−^
543–550s	S_3_^•−^ symmetric stretching (ν_1_) and/or AlF_6_ stretching mode
578–585sh	S_3_^•−^ antisymmetric stretching (ν_3_)
602–612	S_2_^•−^ stretching mode
594–605	Stretching vibrations of the [(S^2−^)Na_4_] cluster
615–673	HF translational modes ?
613–625	SO_4_^2−^ bending vibrations [*F*_2_(ν_4_) mode]
645	*cis*-S_4_ symmetric stretching mode
649–652	*gauche-*S_4_ symmetric stretching vibrations [A_1_(ν_1_) mode]
667–684w	*trans*-S_4_ symmetric stretching ν_3_ mode
802–814	S_3_^•−^ combination mode (ν_1_ + ν_2_)
975–990	SO_4_^2−^ symmetric stretching vibrations [*A*_1_(ν_1_) mode]
1058	CO_3_^2−^ symmetric stretching vibrations
1074	HF libration?
1084–1098	S_3_^•−^ overtone (2’ν_1_)
1135–1152w	SO_4_^2−^ asymmetric stretching vibrations [*F*_2_(ν_3_) mode], possibly, overlapping with S_2_^•−^ overtone (2 × ν_1_)
1160–1166w	S_2_^•−^ overtone (2 × ν_1_) ?
1271–1279w	CO_2_ Fermi resonance
1335	Overtone of the *cis*-S_4_ antisymmetric stretching mode (2 × ν_3_)
1340	Symmetric C–O stretching vibrations of CO_2_ molecules involved in strong dipole–dipole interactions with H_2_O molecules
1349–1350	H···O stretching vibrations of hydrated proton complexes
1351–1363	S_3_^•−^ combination mode (2ν_1_ + ν_2_)
1381	CO_2_ Fermi resonance
1442w	CO_3_ asymmetric stretching mode
1632–1642	S_3_^•−^ overtone (3 × ν_1_)
1894–1908w	S_3_^•−^ combination mode (3 × ν_2_ + ν_1_)
2168–2188	S_3_^•−^ overtone (4 × ν_1_)
2420–2450w	S_3_^•−^ combination mode (4 × ν_2_ + ν_1_)
2553–2581	HS^−^ stretching mode
2691	*cis*-S_4_ antisymmetric stretching (4 × ν_3_)
2712–2730w	S_3_^•−^ overtone (5 × ν_1_)
2904	CH_4_ stretching vibrations
2975w	S_3_^•−^ combination mode (5 × ν_1_ + ν_2_)
3242–3257w	S_3_^•−^ overtone (6 × ν_1_)
3243	H_3_O^+^ stretching mode
3495–3670	H_2_O stretching vibrations
3796	S_3_^•−^ overtone (7 × ν_1_)

## Appendix A

**Table A1:** Data on the chemical composition and synthesis conditions of sodalite-type compounds (with the exception of sodalite *s.s*. and basic sodalite).

Kind of the Framework	Formula	Synthesis Conditions (If Available)	References
[Al–Si–O]	Na_8_(Al_6_Si_6_O_24_)(HS)_2_	Synthesized hydrothermally from kaolin, Na_2_S·10H_2_O, and NaOH at 180 °C to 230 °C for 16–24 h.	[26]
	Na_8_(Al_6_Si_6_O_24_)(NO_3_)_2_	First, dry gel was prepared by the addition of a solution of aluminum nitrate ethanol solution to a solution of tetraethylorthosilicate. The mixture was held at 70 °C for 17 h, then dried in air at 120 °C for 2 h. The final product was synthesized hydrothermally from the dry gel, at 90 °C for 3 h in the presence of NaOH.	[387]
	Na_8_(Al_6_Si_6_O_24_)(OH)(CO_3_)_0.5_·3H_2_O	Synthesized in the Na_2_O-SiO_2_-Al_2_O_3_-Na_2_CO_3_-H_2_O system in the presence of 8M NaOH solution, under hydrothermal conditions, at 80 °C.	[372]
	Na_8_(Al_6_Si_6_O_24_)(CO_3_)	Synthesized in a two-step anion exchange reaction at 700–800 °C in a CO_2_ atmosphere using basic nitrite sodalite as a starting material.	[372]
	Na_8_(Al_6_Si_6_O_24_)Cl_2−*x*_I*_x_*	Prepared hydrothermally using zeolite A as a starting material with different proportions of NaCl and NaI, along with appropriate amount of NaOH, at 100 °C for one week. The products obtained were then heated at 300 °C to evaporate adsorbed water.	[388]
	Na_8_(Al_6_Si_6_O_24_)I_2_	Prepared hydrothermally from AgI and Zeolite 13 X at 150 °C for 48 h.	[186]
	Rb_2_K_6_(Al_6_Si_6_O_24_)(OH)_2_	Ion exchange of the Na analogue with K and Rb nitrate solutions.	[339]
	Na_8_(Al_6_Si_6_O_24_)(WO_4_)_2_	Synthesized by heating a mixture of zeolite X and WO_3_ at 660 °C to 800 °C in air for 12 h.	[389]
	Na_5.12_Ca_1.92_[Al_6_Si_6_O_24_] (MoO_4_)_1.52_	Prepared using a mixture of nepheline gel, anorthite gel, and Na_2_MoO_4_·2H_2_O; first reacted at 1200 °C and 4 kbar during 5–6 h and then at 750 °C and *p* = 3 kbar during 10 days.	[129]
	Na_7.68_[(Al,Si)_12_O_24_] [(Mo_0.65_W_0.35_)O_4_]_0.78_·H_2_O	Prepared using a mixture of nepheline gel, Na_2_MoO_4_·2H_2_O, and Na_2_WO_4_∙ 2H_2_O at 750 °C and 3 kbar.	[129]
	Na_8_(Al_6_Si_6_O_24_)(ReO_4_)_2_	Synthesized in equilibrium with Na–Fe-rich aluminosilicate melt and Re metal at 1100 °C and 500 MPa.	[179]
	Na_8−*x*_(Al_6_Si_6_O_24_)(ReO_4_, CO_3_,SO_4_,MnO_4_,WO_4_,Cl)_2−*y*_	Synthesized from zeolite 4A in binary solutions containing ReO_4_ and a corresponding competing anion in the presence of NaOH, at 90 °C for 96 h.	[185]
	Na_8_[Al_6_Si_6_O_24_](ReO_4_,TcO_4_)_2_	Synthesized using hydrothermal methods by treating zeolite 4A with 8 M NaOH in the presence different amounts of sodium perrhenate and/or sodium pertechnetate at 225 °C for 7 days.	[178]
	Na_8_[Al_6_Si_6_O_24_](ReO_4_)_2_	Synthesized hydrothermally from NaAlO_2_, Na_2_SiO_3_·9H_2_O, and NaReO_4_, in the presence of NaOH at 175 °C for 24 h.	[130]
	Na_8_[Al_6_Si_6_O_24_](ReO_4_,NO_3_)_2_	Synthesized hydrothermally from zeolite A in sodium hydroxide, nitrate, and perrhenate solutions at 90 °C for 24 h.	[181,182]
	Na_8_[Al_6_Si_6_O_24_](SO_4_)·*n*H_2_O	Obtained by precipitation of the desilication product, LTA zeolite, in synthetic Bayer liquor prepared from gibbsite, at 90 °C, in the presence of Na_2_SO_4_.	[390]
	Ca_8_(Al_12_O_24_)(SO_4_)_2_	Synthesized in a solid-state reaction at 1300 °C using calcite, Al_2_O_3_, and gypsum as the starting materials.	[116]
	Na_7+*x*_[Si_5+2*x*_Al_7–2*x*_O_24_](AlF_6_)*_x_*(H_2_O)_4–4*x*_·4H_2_O (*x* = 0–1).	Synthesized hydrothermally in the Si–Al–Na–H–O–F system at temperatures of 400–800 °C and H_2_O pressures of 1–2 kbar. The crystal structure is solved.	[131]
	Na_7.38_[Si_6.74_Al_5.26_O_24_] (AlF_6_)_0.70_(H_2_O)_4–4*x*_·4.88H_2_O	Synthesized hydrothermally from a mixture of nepheline gel and NaF, in the presence of natural sodalite added as a seed, at 650 °C and H_2_O pressure of 2 kbar. The crystal structure is solved.	[132]
	Ag_8_[Al_6_Si_6_O_24_]I_2_	Iodine was captured from the vapour phase using a silver exchanged zrolite and converted to sodalite in hot-isostatic pressing canisters.	[190]
	Na_8_(Al_6_Si_6_O_24_)(NO_2_)_2_	Prepared hydrothermally from kaolin, sodium nitrite, and NaOH at 570 K and 0.1 GPa.	[174]
	Na_7.7_(Al_6_Si_6_O_24_)_6_(MnO_4_)_1.7_ ·0.8H_2_O	Synthesized hydrothermally from Na_2_SiO_3_, NaAlO_2_, and NaMnO_4_·H_2_O in a molar ratio of 1:1:2.6, in the presence of NaOH, at 80 °C, for 48 h.	[156]
	Na_6_Mn_2_[Al_6_Si_6_O_24_](SO_4_)_2_	Prepared by reacting zeolite A, Na_6_(Al_6_Si_6_O_24_), with MnSO_4_ as a compacted mixed-powder monolith at 650 °C under air.	[391]
	Na_6_Zn_2_[Al_6_Si_6_O_24_](SO_4_)_2_	Prepared in a solid-state reaction of a charge containing appropriate amounts of ZnSO_4_·H_2_O and zeolite A at 700 °C for 8 h.	[369]
	Li_8_[Al_6_Si_6_O_24_]Cl_2_	No data.	[392]
	Li_7.4_Na_0.6_[Al_6_Si_6_O_24_](ClO_4_)_2_	Prepared by ion exchange of the sodium perchlorate sodalite (synthesized hydrothermally from the sodium silicate glass, NaClO_4_ and NaOH at 120 °C under reflux, for 24 h) with LiCl solution at 110 °C for 48 h.	[393]
	Na_8_(Al_6_Si_6_O_24_)Br_2_	Synthesized hydrothermally from fly ash and NaBr at 500–550 °C for 2 h.	[394]
	(Na,Ag)_8_[Al_6_Si_6_O_24_]*X*_2_ (*X* = Cl, Br, I)	Aqueous exchanges were used to replace Na^+^ by Ag^+^ in hydrothermally synthesized Na_8_[Al_6_Si_6_O_24_]*X*_2_ sodalites (*X* = Cl, Br, I).	[395,396]
	Na_8_[Si_6_Al_6_O_24_]_6_·(ClO_4_)_2_	Synthesized by addition of sodium aluminate solution to a concentrated solution of sodium perchlorate and sodium silicate.	[377]
	Na_8_[Si_6_Al_6_O_24_]_6_·(ClO_4_)_2_	Synthesized by addition of sodium aluminate solution to a concentrated solution of sodium perchlorate and sodium silicate.	[152]
	K_7.7_Na_0.3_[Al_6_Si_6_O_24_](ClO_4_)_2_	Prepared by ion exchange of the sodium perchlorate sodalite (synthesized hydrothermally from the sodium silicate glass, NaClO_4_ and NaOH at 120 °C under reflux, for 24 h) with KCl solution at 110 °C for 48 h.	[393]
	Na_8_[Al_6_Si_6_O_24_](*X*O_4_)_2_ (*X* = Cl, Mn, Re)	Partly prepared hydrothermally from NaAlO_2,_ Na_2_SiO_3_ and corresponding Na salts (perchlorate, permanganate, and perrhenate), at 230 °C for perrhenate.	[183,397]
	Na_8_[Al_6_Si_6_O_24_](*X*O_4_)_2_ (*X* = Cl, Mn)	Synthesized hydrothermally from the sodium silicate glass and NaClO_4_ or NaMnO_4_ in the presence of NaOH at 120 °C under reflux, for 24 h.	[393]
	Na_8−*x*_Li*_x_*[Al_6_Si_6_O_24_](ClO_3_)_2−*y*_(OH)*_y_*	Obtained by aqueous cation exchange of a pure sodium analogue.	[398]
	K_8−x_Na_x_[Al_6_Si_6_O_24_](XO_3_)_2−*y*_(OH)*_y_* (X = Cl, Br)	Obtained by aqueous cation exchange of a pure sodium analogue.	[398]
	Na_8_[Al_6_Si_6_O_24_](XO_3_)_2−*y*_(OH)*_y_* (*X* = Cl, Br)	Synthesized by a low-temprtature hydrothermal method.	[398]
	Li_8−*x*_Na*_x_*[Al_6_Si_6_O_24_](BrO_3_)_2−*y*_(OH)*_y_*	Obtained by aqueous cation exchange of a pure sodium analogue.	[398]
	Na_8_[Si_6_Al_6_O_24_]_6_·(IO_3_)_2_	Synthesized by hydrothermal treatment from coal fly ash, NaIO_3_, and NaOH at 100 °C	[197]
	Na_8_[Al*_x_*Ga_6−*x*_Si_6_O_24_](NO_2_)_2_ 0 ≤ *x* ≤ 6)	Prepared hydrothermally from stoichiometric quantities of Na_2_SiO_3_, NaAlO_2_, NaGaO_2_, and NaNO_2_, in the presence of NaOH, at 200 °C for 48 h.	[399]
	K_8_[Al_6_Si_6_O_24_]Cl_2_	No data.	[392]
	Ag_6_[Al_6_Si_6_O_24_]	Prepared by ion exchange of hydrous hydroxysodalite, Na_8_[AlSiO_4_]_6_(OH)_2_·2H_2_O, with 0.7 M aqueous AgNO_3_ solution in two 24 h cycles, at 70 °C.	[400]
	Tl_6_[Al_6_Si_6_O_24_]	Prepared by ion exchange of hydrous hydroxysodalite, Na_8_[AlSiO_4_]_6_(OH)_2_·2H_2_O, with 1 M aqueous TlNO_3_ solution at 100 °C.	[400]
	(C_4_H_12_N)[Al_2_Si_10_O_24_]	Synthesized under hydrothermal conditions in the absence of metal cations.	[401]
	Na_8_[Si_6_Al_6_O_24_]_6_·(MnO_4_)_2_	Synthesized by addition of sodium aluminate solution to a concentrated solution of sodium permanganate and sodium silicate.	[156]
	Na_8_(Al_6_Si_6_O_24_)(BH_4_)_2_	Synthesized hydrothermally from NaAlO_2_, Na_2_SiO_3_, and NaBH_4_ at 60 °C for 12 h.	[217]
	~K_4_Na_4_(Al_6_Si_6_O_24_)(BH_4_)_2_	Produced by cation exchange between the Na_8_(Al_6_Si_6_O_24_)(BH_4_)_2_ sodalite-type compound and 1 mol/l aqueous solution of KNO_3_ at 200 °C for 48 h.	[175]
	Na_8_[AlSiO_4_]_6_(BH_4_)_2_	Synthesized hydrothermally from kaolinite, NaOH, and NaBH_4_, at 100 °C, for 24 h.	[219]
	Na_8_[AlSiO_4_]_6_(N_3_)_2_	Synthesized hydrothermally from zeolite A and NaN_3_, at 160 °C, for two weeks.	[162]
	Na_8_[Al_6_Si_6_O_24_]I_2_	Prepared hydrothermally from zeolite 4A, kaolinite, and meta-kaolin, as well as colloidal silica and NaAlO_2_ at 140–180 °C. Decreasing the Al/Si ratio by half increased the crystallization of basic cancrinite.	[402]
[Al–Fe^3+^–Si–O]	(Na,Fe^2+^)_8−*x*_[(Si,Al,Fe^3+^)_12_O_24_] (OH,Cl)_2_·*n*H_2_O?	Synthesized from Fe- and Al-containing gels, prepared from Fe(NO_3_)_3_·9H_2_O and AlCl_3_·6H_2_O in hydrochloric acid solution, and Aldrich type waterglass at 88 °C for 2–2.5 h.	[403]
[Al–P–O]	(C_3_H_7_NO)_2_[Al_6_P_6_O_24_]·*n*H_2_O	Synthesized hydrothermally.	[153,404,405,406,407]
[Mg–Al–P–O]	(C_4_H_12_N)_2_[Mg_2_Al_4_P_6_O_24_]	Prepared hydrothermally from a gel based on tetramethylammonium hydroxide, MgO, Al_2_O_3_, and P_2_O_5_.	[408]
[Al–O]	Ca_8_[Al_12_O_24_]S_2_	Obtained by reduction of the corresponding sulfate material, Ca_4_[Al_96_O_192_]·(SO_4_)_16_ via an intra-cage reaction.	[409,410]
	Ca_8_[Al_12_O_24_]*X*_2_ (*X* = O, Te)	Prepared by solid-state reaction of a stoichiometric mixture of CaO, Al_2_O_3_, and CaTe (for *X* = Te) at 1100 °C for 48.	[411]
	Cd_8_[Al_12_O_24_]O_2_	Prepared by solid-state reaction of a stoichiometric mixture of CdO and Al_2_O_3_ at 1100 °C for 48.	[411]
	Ca_8_[Al_12_O_24_](*X*O_4_)_2_ (*X* = S, Mo, W, Cr)	Synthesized in solid-state reactions.	[154,363,412,413,414,415]
	Sr_8_[Al_12_O_24_]*X*_2_ (*X* = S, Te)	Prepared by reduction of the corresponding sulfate and tellurite materials via intra-cage reactions.	[409]
	Sr_8_[Al_12_O_24_](TeO_3_)_2_	Prepared using solid-state synthesis from the stoichiometric mixture of oxides at 1200 °C for 48 h.	[416]
	Sr_8_[Al_12_O_24_]Te_2_	Prepared by the reduction of Sr_8_[Al_12_O_24_](TeO_3_)_2_ in a stream of hydrogen at 850 °C for 8 h.	[416]
	Sr_8_[Al_12_O_24_](*X*O_4_)_2_ (*X* = Mo, W, Cr)	Crystals partly grown from the B_2_O_3_ flux.	[417,418,419,420]
	Cd_8_[Al_12_O_24_]Te_2_	Prepared by solid-state reaction of a stoichiometric mixture of CdO, Al_2_O_3_ and CdTe at 1100 °C for 48.	[411]
	Pb_8_*REE*_4_[Al_12_O_24_]O_8_ (*REE* = Ho, Lu)	Crystals grown from the melts prepared from corresponding oxides and PbF_2_ at 850 °C for 7 days.	[421]
[Si–O]	SiO_2_	Prepared by thermal treatment of ethylene glycol silica sodalite at 680 °C.	[422]
	SiO_2_	Obtained by heating the AcOH-treated double four-ring lamellar 8TMA(Si_8_O_20_)·*x*H_2_O precursor (TMA = tetramethylammonium) at 800 °C for one week.	[423]
	SiO_2_	Silica sodalite formed as a result of the interlayer condensation of the layered silicate RUB-15 during a heat treatment at 800 °C in an inert atmosphere.	[424]
	(C_2_H_4_(OH)_2_)_2_[Si_12_O_24_]	Partly synthesized from essentially nonaqueous medium in which ethylene glycol is acting both as a solvent and as a structure-directing agent.	[425,426,427,428]
	(C_3_H_6_O_3_)_2_[Si_12_O_24_]	Synthesized from essentially nonaqueous medium in which 1,3,5-trioxane is acting both as a solvent and as a structure-directing agent.	[117,354]
	□_2−*x*_(C_3_H_6_O_3_)*_x_*[Si_12_O_24_]	Synthesized from essentially nonaqueous medium in which 1,3,5-trioxane is acting both as a solvent and as a structure-directing agent.	[354]
[Al–Ga–Si–O]	Na_6_[Al_1–*y*_Ga*_y_*SiO_4_]_6_(H_2_O)_8_ (0 ≤ y ≤ 1)	Prepared from Na_6_[Al_1–*y*_Ga*_y_*SiO_4_]_6_(OH·H_2_O)*_x_*(H_2_O)_8–4*x*_ sodalites by NaOH/H_2_O exchange methods carried out in acidic aqueous media at pH = 5.5–6.6.	[429]
[Ga–Si–O]	Na_6_[GaSiO_4_]_6_(H_2_O)_8_	Obtained from Na_8_[GaSiO_4_]_6_I_2_ by a hydrothermal transformation process in the excess of water, at 200 °C for 24 h.	[429]
	Na_8_[Ga_6_Si_6_O_24_]*X*_2_ (*X* = Cl, Br, I)	Prepared under hydrothermal conditions by heating aqueous solutions of NaOH, Ga_2_O_3_, Na_2_SiO_3_, and Na*X* for at 160 °C for 100 h.	[352]
	Li_8_[Ga_6_Si_6_O_24_]*X*_2_ (*X* = Cl, Br, I)	Obtained by replacement of Na^+^ by Li^+^ in sodalite of composition Na_8_[GaSiO_4_]_6×2_, where X is a halide, in nitrate melt at 240 °C.	[430]
	Na_6_[Ga_6_Si_6_O_24_]·8H_2_O	Synthesized hydrothermally from corresponding sodium salts.	[364,377,378,379,380]
	Na_8_[Ga_6_Si_6_O_24_]*X*_2_ (*X* = OH, Cl, Br, I)	Synthesized hydrothermally from corresponding sodium salts.	[352,377,431]
	Na_8_[Ga_6_Si_6_O_24_](*X*O_4_)_2_ (*X* = Cl, Mn)	Synthesized hydrothermally from corresponding sodium salts.	[162,432]
	Na_8_[Ga_6_Si_6_O_24_](*X*O_3_)_2_ (*X* = Cl, N, C)	Synthesized hydrothermally from corresponding sodium salts.	[377]
	Na_8_[Ga_6_Si_6_O_24_](NO_2_)_2_	Synthesized hydrothermally from corresponding sodium salts.	[353,377,433]
	Na_8_[Ga_6_Si_6_O_24_](BH_4_)_2_	Synthesized under mild hydrothermal conditions from NaAlGeO_4_ and NaBH_4_ at 80–120 °C for 12–48 h. Increasing temperatures above 120 °C favor the decomposition of the tetrahydroborate anions before encapsulation within the sodalite framework cavities.	[434]
	Na_8_[Ga_6_Si_6_O_24_](SCN)_2_	Synthesized hydrothermally from corresponding sodium salts.	[377]
	Na_8_[Ga_6_Si_6_O_24_](HCO_2_)_2_	Synthesized hydrothermally from corresponding sodium salts.	[377]
	K_4.88_Na_3.12_[Ga_6_Si_6_O_24_](NO_2_)_2_	Obtained by cation exchange of a pure sodium analogue at 100 °C.	[435]
	Ag_5.98_Na_2.02_[Ga_6_Si_6_O_24_](NO_2_)_2_	Obtained by cation exchange of a pure sodium analogue at 100 °C.	[435]
[Be–Si–O]	Na_8_[Be_3_Si_9_O_24_]Cl_2_	Prepared by a solid-state synthesis method from a sodium silicate glass, Na_6_Si_9_O_21_, stoichiometric quantity of BeO, and a four-fold excess of NaCl at 800 °C for 48 h.	[436]
	Mn_8_[Be_6_Si_6_O_24_]*X*_2_ (*X* = S, Se, Te)	Synthesized by solid state reactions of the constituent oxides and Mn*X* at 1000−1100 °C.	[118]
	Cd_8_[Be_6_Si_6_O_24_]*X*_2_ (*X* = S, Se, Te)	Synthesized by hydrothermal reaction of CdO, BeO, SiO_2_, and *X* at 750 °C and 2 kbar.	[437]
[Be–Si–O]	Na_6_[Zn_6_As_6_O_24_]·8H_2_O	Synthesized hydrothermally from Na_2_HSO_4_·7H_2_O, NaOH, and Zn(NO_3_)_2_ at 70 °C for 12 h.	[378,438]
[Zn–P–O]	Na_6_[Zn_6_P_6_O_24_]·Br_2_? (No data on the chemical composition are provided)	Synthesized from a solution containing Zn(NO_3_)_2_, (H_3_PO_4_), NaBr, and NaOH at pH 7.	[439]
[Ga–As–O]	BaGe_8_As_14_	Synthesized via solid-state reactions. No details provided.	[151]
[Zn–P–O]	Na_6_[Zn_6_P_6_O_24_]·8H_2_O	Synthesized hydrothermally from ZnO, H_3_PO_4_, and NaOH at 50 °C for 12 h.	[438]
[Be–P–O]	Li_8_[Be_6_P_6_O_24_]*X*_2_ (*X* = Cl, Br)	Synthesized at 550 °C and 0.3447 GPa.	[119,440]
[Al–Ge–O]	Na_8_(Al_6_Ge_6_O_24_)*X*_2_ (*X* = Cl, Br, I)	Crystallyzed by solvothermal method at 120 °C, using 50% ethanol + 50% water as a solvent, and NaAlO_2_, GeO_2_, Na*X*, and NaOH as rectants.	[128]
	Na_8_(Al_6_Ge_6_O_24_)(ClO_3_)_2_	Synthesized hydrothermally from NaAlO_2_, GeO_2_, NaClO_3_, and NaOH at 120 °C for 120 h.	[158]
	Na_8_(Al_6_Ge_6_O_24_)_6_(SCN)_2_	Synthesized hydrothermally from GeO_2_ and NaAlO_2_ in the presence of NaOH, at 150 °C for three days.	[161]
	Na_8_(Al_6_Ge_6_O_24_)Br_2_	Synthesized from NaAlO_2_, GeO_2_, SiO_2_, and NaOH under hydrothermal conditions at 320–450°.	[150]
	Li_8_[Al_6_Ge_6_O_24_]*X*_2_ (*X* = Cl, Br, I)	Obtained by replacement of Na^+^ by Li^+^ in sodalite of composition Na_8_[AlGeO_4_]_6×2_, where X is a halide, in nitrate melt at 240 °C.	[430]
	Na_6_[Al_6_Ge_6_O_24_]·8H_2_O	Synthesized hydrothermally from corresponding sodium salts.	[377,378]
	Na_6+*x*_[Al_6_Ge_6_O_24_](OH)*_x_*·3H_2_O	Synthesized by reacting Al_2_O_3_, GeO_2_, and NaOH solution under mild hydrothermal conditions.	[441]
	Na_8_[Al_6_Ge_6_O_24_]*X*_2_ (*X* = OH, Cl, Br, I)	Synthesized hydrothermally from sodium aluminate, GeO_2_ and corresponding sodium salts.	[120,377,442,443]
	Na_8_[Al_6_Ge_6_O_24_](S_2_/S_3_)_2_	Synthesized hydrothermally from corresponding sodium salts.	[377]
	Na_8_[Al_6_Ge_6_O_24_](Se_2_)_2_	Synthesized hydrothermally from corresponding sodium salts.	[377]
	Na_8_[Al_6_Ge_6_O_24_](*X*O_4_)_2_ (*X* = Cl, S, Mn)	Synthesized hydrothermally from sodium aluminate, GeO_2_, and corresponding sodium salts.	[377,444]
	Na_8_[Al_6_Ge_6_O_24_](NO_2_)_2_	Synthesized hydrothermally in the system Na_2_O–GeO_2_–Al_2_O_3_–NaNO_2_–H_2_O at 200 °C.	[445]
	Na_8_[Al_6_Ge_6_O_24_](*X*O_3_)_2_ (*X* = Cl, Br, N, C, Se)	Synthesized hydrothermally from corresponding sodium salts.	[377]
	Na_8_[Al_6_Ge_6_O_24_](SCN)_2_	Synthesized hydrothermally from corresponding sodium salts.	[377]
	Na_8_[Al_6_Ge_6_O_24_](HCO_2_)_2_	Synthesized hydrothermally from corresponding sodium salts.	[377]
	Na_8_[Al_6_Ge_6_O_24_](H_3_C_2_O_2_)_2_	Synthesized hydrothermally from corresponding sodium salts.	[377]
	K_8−*x*_Na*_x_*[Al_6_Ge_6_O_24_](ClO_4_)_2_	Obtained by cation exchange of corresponding pure Na analogue.	[444]
[Zn–Si–O]	(C_4_H_12_N)_2−*x*_Na*_x_*[Zn_1.6_Si_10.4_O_24_]	Obtained from a silicate gel with included Zn^2+^.	[446]
[Ga–Co–Si–O]	(C_4_H_10_N_2_)_2_[Ga_2_Co_4_P_6_O_24_]	Synthesized hydrothermally from C_4_H_10_N_2_, Ga_2_O_3_, and Co phosphate.	[447,448]
[Be–Ge–O]	Mn_8_[Be_6_Ge_6_O_24_]*X*_2_ (*X* = S, Se)	Synthesized by solid state reactions of the constituent oxides and MnX at 1000−1100 °C in sealed quartz ampoules.	[118]
[Al–Co–P–O]	(C_4_H_10_N_2_)_2_[Al_2_Co_4_P_6_O_24_]	Synthesized either hydrothermally from C_4_H_10_N_2_, Al_2_O_3_, and Co phosphate or by an ionothermal method (an ionic liquid as both solvent and structure-directing agent has been used).	[447,449]
[Zn–Ga–P–O]	(C_4_H_12_N)_2_[Zn_2_Ga_4_P_6_O_24_]	Synthesized hydrothermally from C_4_H_12_N, Ga_2_O_3_, and Zn phosphate.	[448]
[Zn–Ga–As–O]	(C_4_H_12_N)_2_[Zn_2_Ga_4_As_6_O_24_]	Synthesized hydrothermally from C_4_H_12_N, Ga_2_O_3_, and Zn arsenate.	[448,450]
	(C_4_H_10_N_2_)_2_[Zn_4_Ga_2_As_6_O_24_]	Synthesized hydrothermally from C_4_H_10_N_2_, Ga_2_O_3_, and Zn arsenate.	[450]
[Zn–Al–As–O]	(C_4_H_12_N)_2_[Zn_2_Al_4_As_6_O_24_]	Obtained by successive adding of As_2_O_5_ + C_4_H_12_N, Al nitrate, Zn arsenate, and HNO_3_ to a mixed water–ethylene glycol solvent to pH of 5.25 and heating at 150 °C for 4 days.	[450]
[Ga–Ge–O]	Na_8−*x*_(Ga_6_Ge_6_O_24_) [B(OH)_4_]_2−*x*_(H_2_O)*_x_* (?)	Synthesized hydrothermally, using NaGaGeO_4_ and Na[B(OH)_4_] as the starting materials, at 120 °C for 24 h.	[451]
	(Am)_2_[Ga_2_Ge_10_O_24_] (Am = ammonium cation)	Synthesized hydrothermally from GeO_2_, Ga(NO_3_)_3_·*n*H_2_O, and corresponding amine at 180 °C for 8 days.	[452]
	Na_6_[Ga_6_Ge_6_O_24_]·*x*H_2_O	Prepared from sodium germanate and sodium gallate in aqueous sodium tetramethylammonium hydroxide solution, in the presence of Na_2_SO_4_, at 100 °C.	[377,453]
	Na_8_[Ga_6_Ge_6_O_24_](OH)_2_·6H_2_O	Prepared from sodium germanate and sodium gallate in aqueous sodium tetramethylammonium hydroxide solution, in the presence of Na_2_SO_4_, at 100–150 °C.	[453]
	Na_8_[Ga_6_Ge_6_O_24_]X_2_ (X = Cl, Br, I)	Synthesized hydrothermally from corresponding sodium salts.	[377]
	Na_8_[Ga_6_Ge_6_O_24_](ClO_4_)_2_	Synthesized hydrothermally from corresponding sodium salts.	[377]
	Na_8_[Ga_6_Ge_6_O_24_](NO_2_)_2_	Synthesized starting with the formation of gallogermanate basic-hydro-sodalite under hydrothermal conditions, at 100 °C, followed by hydrothermal treatment in concentrated NaNO_2_ solution.	[454]
	Na_8_[Ga_6_Ge_6_O_24_](BH_4_)_2_	Separate suspensions of NaGaGeO_4_ and NaBH_4_ in sodium hydroxide solutions were combined before the hydrothermal treatment in the presence of NaOH at 150 °C for 4 h.	[370]
[Al–Be–Si–O]	Na_8_[Al_2_Be_2_Si_8_O_24_]Cl_2_	Prepared by a solid-state synthesis method from a sodium silicate glass, Na_6_Si_8_O_19_, stoichiometric quantities of BeO and Al_2_O_3_, and a four-fold excess of NaCl at 800 °C for 48 h.	[436]
	Na_8_[Al_4_BeSi_7_O_24_]*X*_2_ (*X* = Cl, Br)	Prepared by a solid-state synthesis method from a sodium silicate glass, Na_6_Si_7_O_17_, stoichiometric quantities of BeO and Al_2_O_3_, and a four-fold excess of NaCl or NaBr at 800 °C for 48 h.	[436]
[Be–As–O]	Li_8_[Be_6_As_6_O_24_]Cl_2_	No data.	[119]
[B–O]	(Zn,Ga)_8_(B_12_O_24_)(Se,P)_2_	Prepared by a solid-state synthesis method from a mixture of ZnB_4_O_7_, ZnO, ZnSe, and GaP at 900–950 °C for 12–36 h.	[121]
	Co[B_12_O_24_]S_2_	Synthesized from a stoichiometric mixture of B_2_O_3_, CoO, and CoS at 920 °C.	[455]
	Zn[B_12_O_24_]S_2_	Synthesized from a stoichiometric mixture of B_2_O_3_, ZnO, and ZnS at 920 °C.	[455]
	(Zn,Mn)_4_[B_6_O_12_]O	Synthesized via solid-state reaction from ZnO, H_3_BO_3_ and MnCO_3_, first at 500 °C for 5 h in air atmosphere, then at 700 and 900 °C for 7 h in a reduced carbon atmosphere.	[304]
[B–Ge–O]	Cs_2_[B_2_Ge_10_O_24_]	Obtained by the solvothermal reaction of H_3_BO_3_, GeO_2_, CsCl, HF, pyridine, and H_2_O at 200 °C for 7 days.	[456]
Organic and metal–organic	[(CH_3_)_2_NH_2_]_4_[In_6_(BTC)_12_]_2_[(M_3_OH)_4_(H_2_O)_36_][(In_2_MO)_4_(BTC)_4_(H_2_O)_12_]·(solvent)*_x_* (M = Mg, Mn, Co, Ni, and Cd).	Synthesized by a single-step low-temperature method of simultaneous formation of sodalite-type frameworks and covalent attachment of transition- and non-transition-metal clusters in a solution containing 1,3,5-benzenetricarboxylate (BTC).	[457,458]
	Zn(im)_2_ (zinc imidazolate)	Synthesized mechanochemically at room temperature.	[459,460]
	Zn(im)_2_·*n*C_60_ (im = imidazolate, *n* = 0.15–1)	Fullerene C_60_ was entrapped in the cages of the sodalite-type imidazolate framework by mechanochemical processing.	[460]
	Zn(im)_2_ (zinc imidazilate)	Synthesized via solvent-assisted linker exchange of ZIF-8 (Zn(Me-im)_2_).	[461]
	Zn(5-mtz)(2-eim)	Obtained by heating a mixture of Zn(CH_3_CO_2_)_2_·2H_2_O, 5-methyltetrazole (5-mtz) and 2-ethylimidazole (2-eim) in dimethylformamide and ethanol to 120 °C.	[329,330,331]
	Zn(Me-im)_2_	Synthesized from methyl imidazol (Me-im) and a Zn salt at room temperature under aqueous conditions for 10 min.	[462]
	Co(Me-im)_2_	Synthesized from methyl imidazol (Me-im) and a Co salt at room temperature under aqueous conditions for 10 min.	[462]
	[(Cu_4_Cl)_3_(H_0.5_BTT)_8_(H_2_O)_12_]·3MeOH·9DMF	Prepared from CuCl_2_, 5,5′-(1,4-phenylene) bis(1H-tetrazole) (H_3_BTT), methanol, and dimethyl formamide (DMF).	[316]
	Cu_3_[(Cu_4_Cl)_3_(TPB-3tz)_8_]_2_·11CuCl_2_·8H_2_O·120DMF	Obtained in the reactions of 2,4,6-tri-p-(tetrazol-5-yl)-phenyl-s-triazine (H_3_TPB-3tz) with CuCl_2_in the presence of dimethyl formamide (DMF).	[205]
	Mn_3_[(Mn_4_Cl)_3_(TPT-3tz)_8_]_2_ ·25H_2_O·15CH_3_OH·95DMF	Obtained in the reactions of 2,4,6-tri-p-(tetrazol-5-yl)-phenyl-s-triazine (H_3_TPB-3tz) with MnCl_2_ in the presence of dimethyl formamide (DMF).	[205]
	Cu_3_[(Cu_4_Cl)_3_(TPT-3tz)_8_]_2_·*x*(solvent)	Obtained in the reactions of 2,4,6-tri-p-(tetrazol-5-yl)- phenyl-s-triazine (H_3_TPB-3tz) with CuCl_2_ in different solvents.	[205]
	ML_2_ (M = Pd^II^ or Cu^II^, L = 2-hydroxypyrimidine or 4-hydroxypyrimidine)	Prepared by a solid–liquid sorption method.	[233]
	(Et_2_NH_2_)_3_[(Cu_4_Cl)_3_(TTCA)_8_] ·26DEF	Synthesized in the solvothermal reaction of triphenylene-2,6,10-tricarboxylic acid (H_3_TTCA)_9_ with CuCl_2_·2H_2_O in N,N-diethylformamide (DEF) at 110 °C for 48 h.	[214]
	Li_3_[(Cu_4_Cl)_3_(TTCA)_8_]·26DEF	Ion exchange of (Et_2_NH_2_)_3_[(Cu_4_Cl)_3_(TTCA)_8_]·26DEF with Li^+^.	[214]
	Fe_3_[(Fe_4_Cl)_3_(BTT)_8_]_2_·22DMF ·32DMSO·11H_2_O	Synthesized in the reaction between FeCl_2_ and H3BTT·2HCl (BTT_3_^−^ = 1,3,5-benzenetristetrazolate) in a mixture of dimethylformamide (DMF) and dimethylsulfoxide (DMSO).	[215]
	[Zn(HL)]·DMA	Solvothermally synthesized based on an N-rich aromatic ligand L = 4,5-di(1H-tetrazol-5-yl)-2H-1,2,3-triazole in dimethyl acetate (DMA)	[230]

## References

[1] Peterson R.C. (1983). The structure of hackmanite, a variety of sodalite, from Mont St-Hilaire, Quebec. Can. Mineral..

[2] Hassan I., Grundy H.D. (1984). The crystal structures of sodalite-group minerals. Acta Crystallogr. Sect. B Struct. Sci..

[3] Löhn J., Schulz H. (1968). Strukturverfeinerung am gestörten Haüyn, (Na_5_K_1_Ca_2_)Al_6_Si_6_O_24_(SO_4_)_1.5_. Neues Jahrb. Mineral. Abh..

[4] Hassan I., Grundy H.D. (1991). The crystal structure of haüyne at 273 and 153 K. Can. Mineral..

[5] Hassan I., Peterson R.C., Grundy H.D. (1985). The structure of lazurite, ideally Na_6_Ca_2_(Al_6_Si_6_O_24_)S_2_, a member of the sodalite group. Acta Crystallogr. Sect. C Cryst. Struct. Commun..

[6] (2020). IMA, List of Mineral Species. http://cnmnc.main.jp/.

[7] Sapozhnikov A.N., Tauson V.L., Lipko S.V., Shendrik R.Y., Levitskii V.I., Suvorova L.F., Chukanov N.V., Vigasina M.F. (2020). On the crystal chemistry of sulfur-rich lazurite, ideally Na_7_Ca(Al_6_Si_6_O_24_)(SO_4_)(S_3_)^–^·*n*H_2_O. Am. Mineral..

[8] Hassan I., Grundy H.D. (1989). The structure of nosean, ideally Na_8_[Al_6_Si_6_O_24_]SO_4_∙H_2_O. Can. Mineral..

[9] Sahl K. (1980). Refinement of the crystal structure of bicchulite, Ca_2_[Al_2_SiO_6_](OH)_2_. Z. Krist. Cryst. Mater..

[10] Uchida E., Iiyama J.T. (1981). On kamaishilite, Ca_2_Al_2_SiO_6_(OH)_2_, a new mineral dimorphous (tetragonal) with bicchulite from the Kamaishi mine, Japan. Proc. Jpn. Acad. Ser. B Phys. Biol. Sci..

[11] Lee S., Xu H., Xu H., Jacobs R., Morgan D. (2019). Valleyite: A new magnetic mineral with the sodalite-type structure. Am. Mineral..

[12] Sapozhnikov A.N., Kaneva E.V., Cherepanov D.I., Suvorova L.F., Levitsky V.I., Ivanova L.A., Reznitsky L.Z. (2012). Vladimirivanovite, Na_6_Ca_2_[Al_6_Si_6_O_24_](SO_4_,S_3_,S_2_,Cl)_2_·H_2_O, a new mineral of sodalite group. Geol. Ore Depos..

[13] Sokolova E.V., Rybakov V.B., Pautov L.A. (1991). Crystal structure of a new natural tetramethyammonium aluminosilicate [N(CH_3_)_4_][Si_2_(Si_0.5_Al_0.5_)O_6_]_2_. Proc. USSR Acad. Sci..

[14] Pautov L.A., Karpenko V.Y., Sokolova E.V., Ignatenko K.I. (1993). Tsaregorodtsevite N(CH_3_)_4_[Si_2_(Si_0.5_Al_0.5_)O_6_]_2_—A new mineral. Zap. Vses. Miner. Obsh..

[15] Hassan I., Grundy H.D. (1991). The crystal structure and thermal expansion of tugtupite, Na_8_[Al_2_Be_2_Si_8_O_24_]Cl_2_. Can. Mineral..

[16] Hassan I., Grundy H.D. (1985). The crystal structures of helvite group minerals, (Mn,Fe,Zn)_8_(Be_6_Si_6_O_24_)S_2_. Am. Mineral..

[17] Chukanov N.V., Zubkova N.V., Pekov I.V., Shendrik R.Y., Varlamov D.A., Vigasina M.F., Belakovskiy D.I., Britvin S.N., Yapaskurt V.O., Pushcharovsky D.Y. (2022). Sapozhnikovite, Na_8_(Al_6_Si_6_O_24_)(HS)_2_, a new sodalite-group mineral from the Lovozero alkaline massif, Kola Peninsula. Mineral. Mag..

[18] Sapozhnikov A.N., Bolotina N.B., Chukanov N.V., Shendrik R.Y., Kaneva E.V., Vigasina M.F., Ivanova L.A., Tauson V.L., Lipko S.V. (2023). Slyudyankaite, Na_28_Ca_4_(Si_24_Al_24_O_96_)(SO_4_)_6_(S_6_)_1/3_(CO_2_)·2H_2_O, a new sodalite group mineral from the Malo-Bystrinskoe lazurite deposit, Baikal Lake area, Russia. Am. Mineral..

[19] Dunn P.E. (1976). Genthelvite and the helvine group. Mineral. Mag..

[20] Sapozhnikov A.N., Chukanov N.V., Shendrik R.Y., Vigasina M.F., Tauson V.L., Lipko S.V., Belakovskiy D.I., Levitskii V.I., Suvorova L.G., Ivanova L.A. (2022). Lazurite: Validation as a mineral species with the formula Na_7_Ca(Al_6_Si_6_O_24_)(SO_4_)S_3_^•–^·H_2_O and new data. Geol. Ore Depos..

[21] Chukanov N.V., Zubkova N.V., Schäfer C., Pekov I.V., Shendrik R.Y., Vigasina M.F., Belakovskiy D.I., Britvin S.N., Yapaskurt V.O., Pushcharovsky D.Y. (2022). Bolotinaite, (Na_6_K^−^)(Al_6_Si_6_O_24_)F·4H_2_O, a new sodalite-group mineral from the Eifel paleovolcanic region, Germany. Mineral. Mag..

[22] Chukanov N.V., Aksenov S.M., Rastsvetaeva R.K. (2021). Structural chemistry, IR spectroscopy, properties, and genesis of natural and synthetic microporous cancrinite- and sodalite-related materials: A review. Microporous Mesoporous Mater..

[23] Chukanov N.V., Vigasina M.F., Zubkova N.V., Pekov I.V., Schäfer C., Kasatkin A.V., Yapaskurt V.O., Pushcharovsky D.Y. (2020). Extra-framework content in sodalite-group minerals: Complexity and new aspects of its study using infrared and Raman spectroscopy. Minerals.

[24] Chukanov N.V., Shendrik R.Y., Vigasina M.F., Pekov I.V., Sapozhnikov A.N., Shcherbakov V.D., Varlamov D.A. (2022). Crystal chemistry, isomorphism, and thermal conversions of extra-framework components in sodalite-group minerals. Minerals.

[25] Chukanov N.V., Vigasina M.F., Shendrik R.Y., Varlamov D.A., Pekov I.V., Zubkova N.V. (2022). Nature and isomorphism of extra-framework components in cancrinite- and sodalite-related minerals: New data. Minerals.

[26] Chukanov N.V., Shchipalkina N.V., Shendrik R.Y., Vigasina M.F., Tauson V.L., Lipko S.V., Varlamov D.A., Shcherbakov V.D., Sapozhnikov A.N., Kasatkin A.V. (2022). Isomorphism and mutual transformations of S-bearing components in feldspathoids with microporous structures. Minerals.

[27] NChukanov V., Sapozhnikov A.N., Shendrik R.Y., Vigasina M.F., Steudel R. (2020). Spectroscopic and crystal-chemical features of sodalite-group minerals from gem lazurite deposits. Minerals.

[28] NBolotina B., Sapozhnikov A.N., Chukanov N.V., Vigasina M.F. (2023). Structure modulations and symmetry of lazurite-related sodalite-group minerals. Crystals.

[29] Dumańska-Słowik M., Heflik W., Pieczka A., Sikorska M., Dąbrowa Ł. (2015). The transformation of nepheline and albite into sodalite in pegmatitic mariupolite of the Oktiabrski Massif (SE Ukraine). Spectrochim. Acta A Mol. Biomol. Spectrosc..

[30] Schneider J.B., Jenkins D.M. (2020). Stability of sodalite relative to nepheline in NaCl–H_2_O brines at 750 °C: Implications for hydrothermal formation of sodalite. Can. Mineral..

[31] Hassan I., Grundy H.D. (1983). Structure of basic sodalite, Na_8_Al_6_Si_6_O_24_(OH)_2_·2H_2_O. Acta Crystallogr. Sect. C Cryst. Struct. Commun..

[32] Felsche J., Luger S., Baerlocher C. (1986). Crystal structures of the hydro-sodalite Na_6_[AlSiO_4_]_6_·8H_2_O and of the anhydrous sodalite Na_6_[AlSiO_4_]_6_. Zeolites.

[33] Fazal T. (2011). High Temperature Studies of Sodalites. Master’s Thesis.

[34] Felsche J., Luger S. (1987). Phases and thermal decomposition characteristics of hydro-sodalites Na_6+*x*_[AISiO_4_]_6_(OH)*_x_*·*n*H_2_O. Thermochim. Acta.

[35] Sari M.E.F., Suprapto S., Prasetyoko D. (2018). Direct synthesis of sodalite from kaolin: The influence of alkalinity. Indones. J. Chem..

[36] Khalifah S.N., Cahyawati M., Cahyani D.K.D., Arifah A., Prasetyo A. (2019). Synthesis of sodalite from indonesian kaolin with conventional and alkali fusion method. IOP Conf. Ser. Mater. Sci. Eng..

[37] Reyes C.A.R., Williams C., Mauricio O., Alarcón C. (2013). Nucleation and growth process of sodalite and cancrinite from kaolinite-rich clay under low-temperature hydrothermal conditions. Mater. Res..

[38] Li J., Zeng X., Yang X., Wang C., Luo X. (2015). Synthesis of pure sodalite with wool ball morphology from alkali fusion kaolin. Mater. Lett..

[39] Maia A.Á.B., Neves R.F., Angélica R.S., Pöllmann H. (2015). Synthesis of sodalite from Brazilian kaolin wastes. Clay Miner..

[40] Zhou X., Liu Q., Liu Y. (2012). Ionothermal synthesis of sodalite from metakaolin. Adv. Mater. Res..

[41] Wahyuni T., Prasetyoko D., Suprapto S., Qoniah I., Bahruji H., Dawam A., Triwahyono S., Jalil A.A. (2019). Direct synthesis of sodalite from indonesian kaolin for adsorption of Pb^2+^ solution, kinetics, and isotherm approach. Bull. Chem. React. Eng. Catal..

[42] Esaifan M., Warr L.N., Grathoff G., Meyer T., Schafmeister M.-T., Kruth A., Testrich H. (2019). Synthesis of hydroxy-sodalite/cancrinite zeolites from calcite-bearing kaolin for the removal of heavy metal ions in aqueous media. Minerals.

[43] Yanga J., Li T., Bao X., Yue Y., Liua H. (2020). Mesoporogen-free synthesis of hierarchical sodalite as a solid basecatalyst from sub-molten salt-activated aluminosilicate. Particuology.

[44] Passos F.A.C.M., Castro D.C., Ferreira K.K., Simoes K.M.A., Bertolino L.C., Barbato C.N., Garrido F.M.S., Felix A.A.S., Silva F., Ikhmayies S., Li B., Carpenter J.S., Li J., Hwang J.Y., Monteiro S.N., Firrao D., Zhang M., Peng Z., Escobedo Diaz J.P. (2017). Synthesis and Characterization of Sodalite and Cancrinite from Kaolin.

[45] Golbad S., Khoshnoud P., Abu-Zahra N. (2017). Hydrothermal synthesis of hydroxy sodalite from fly ash for the removal of lead ions from water. Int. J. Environ. Sci. Technol..

[46] Zong Y.-B., Zhao C.-Y., Chen W.-H., Liu Z.-B., Cang D.-Q. (2020). Preparation of hydro-sodalite from fly ash using a hydrothermal method with a submolten salt system and study of the phase transition process. Int. J. Miner. Metall. Mater..

[47] Shabani J.M., Babajide O., Oyekola O., Petrik L. (2019). Synthesis of hydroxy sodalite from coal fly ash for biodiesel production from waste-derived maggot oil. Catalysts.

[48] Luo J., Zhangb H., Yang J. (2016). Hydrothermal synthesis of sodalite on alkali-activated coal fly ash for removal of lead ions. Procedia Environ. Sci..

[49] Oh J.E., Moon J., Mancio M., Clark S.M., Monteiro P.J.M. (2011). Bulk modulus of basic sodalite, Na_8_[AlSiO_4_]6(OH)_2_·2H_2_O, a possible zeolitic precursor in coal-fly-ash-based geopolymers. Cem. Concr. Res..

[50] Musyoka N.M., Petrik L.F., Gitari W.M., Balfour G., Hums E. (2012). Optimization of hydrothermal synthesis of pure phase zeolite Na-P1 from South African coal fly ashes. J. Environ. Sci. Health A.

[51] Sun L., Wu J., Wang J., Yu G., Liu J., Du Y., Li Y., Li H. (2020). Controlled synthesis of Zeolite adsorbent from lowgrade diatomite: A case study of self-assembled sodalite microspheres. J. Environ. Sci..

[52] Kamyab S.M., Williams C.D., Badiei A. (2020). Synthesis of sodalite from sepiolite by alkali fusion method and its application to remove Fe^3+^, Cr^3+^, and Cd^2+^ from aqueous solutions. Environ. Eng. Sci..

[53] Xie L., Wang P., Li Z., Peng H., Li X., Zhou Y. (2018). Preparation and performance study on sodalite/magnetite from tourmaline. Mater. Sci. Eng..

[54] Esaifan M., Hourani M., Khoury H., Rahier H., Wastiels J. (2017). Synthesis of hydroxysodalite zeolite by alkali-activation of basalt powder rich in calc-plagioclase. Adv. Powder Technol..

[55] Jiang J., Gu X., Feng L., Duanmu C., Jin Y., Hu T., Wu J. (2012). Controllable synthesis of sodalite submicron crystals and microspheres from palygorskite clay using a two-step approach. Powder Technol..

[56] Li Y., Peng T., Man W., Ju L., Zheng F., Zhang M., Guo M. (2016). Hydrothermal synthesis of mixtures of NaA zeolite and sodalite from Ti-bearing electric arc furnace slag. R. Soc. Chem. Adv..

[57] Kim J.-C., Choi M., Kim D.-S., Song H.J., Kim D.-W. (2015). Windshield-waste-driven synthesis of hydroxy sodalite. J. Ceram. Soc. Jpn..

[58] Naskar M.K., Kundu D., Chatterjee M. (2011). Coral-like hydroxy sodalite particles from rice husk ash as silica source. Mater. Lett..

[59] Choy H., Lee S.-R., Han Y.-S., Park M., Park G.-S. (2003). Solid–solid transformation mechanism for nanocrystalline sodalite from pillared clay. Chem. Commun..

[60] Belviso C., Cavalcante F., Niceforo G., Lettino A. (2017). Sodalite, faujasite and A-type zeolite from 2:1dioctahedral and 2:1:1 trioctahedral clay minerals. A singular review of synthesis methods through laboratory trials at a low incubation temperature. Powder Technol..

[61] Mundus C., Müller-Warmuth W., Buhl J.-C. (1996). Crystallization of a basic sodalite under hydrothermal conditions studied by MAS-NMR, XRD and DTA/DTG. Eur. J. Mineral..

[62] Fan W., Morozumi K., Kimura R., Yokoi T., Okubo T. (2008). Synthesis of nanometer-sized sodalite without adding organic additives. Langmuir.

[63] You S., Zhang Y., Chen F., Cao S., Zhang Y. (2014). Transformation of NaCaHSiO_4_ to sodalite and katoite in sodium aluminate solution. Hydrometallurgy.

[64] Naskar M.K., Kundu D., Chatterjee M.I. (2011). An aqueous-based synthesis of flower-like hydroxy sodalite particles in the presence of cetyltrimethylammonium bromide. Am. Ceram. Soc..

[65] Lapari S.S., Ramli Z., Triwahyono S. (2015). Effect of different templates on the synthesis of mesoporous sodalite. J. Chem..

[66] Cui L., Han R., Yang L., Wu Y., Pei R., Li F. (2020). Synthesis and characterization of mesoporous sodalite and investigation of the effects of inorganic salts on its structure and properties. Microporous Mesoporous Mater..

[67] Huang Y., Yao J., Zhang X., Kong C., Chen H., Liu D., Tsapatsis M., Hill M.R., Hill A.J., Wang H. (2011). Role of ethanol in sodalite crystallization in an ethanol–Na_2_O–Al_2_O_3_–SiO_2_–H_2_O system. CrystEngComm.

[68] Naskar M.K., Kundu D., Chatterjee M. (2011). Effect of process parameters on surfactant-based synthesis of hydroxyl sodalite particles. Mater. Lett..

[69] Barnes M.C., Addai-Mensah J., Gerson A.R. (1999). The mechanism of the sodalite-to-cancrinite phase transformation in synthetic spent Bayer liquor. Microporous Mesoporous Mater..

[70] Xu B., Smith P., Wingate C., De Silva L. (2010). The effect of calcium and temperature on the transformation of sodalite to cancrinite in Bayer digestion. Hydrometallurgy.

[71] Buhl J.-C. (2016). Enhanced methods of crystallization: The crossover synthesis from gel to melt flow—A case study on sodalites. Microporous Mesoporous Mater..

[72] Zeng S., Wang R., Zhang Z., Qiu S. (2016). Solventless green synthesis of sodalite zeolite using diatomite as silica source by a microwave heating technique. Inorg. Chem. Commun..

[73] Zeng S., Wang R., Li A., Huang W., Zhang Z., Qiu S. (2016). Solvent-free synthesis of nanosized hierarchical sodalite zeolite with a multi-hollow polycrystalline structure. CrystEngComm.

[74] Novembre D., Gimeno D., Pasculli A., Di Sabatino B. (2010). Synthesis and characterization of sodalite using natural kaolinite: An analytical and mathematical to simulate the loss in weight of chlorine during the synthesis process. Fresenius Environ. Bull..

[75] Pascull A., Novembre D. (2012). Phenomenological–mathematical approach in simulating the loss in weight of chlorine during sodalite synthesis. Comput. Geosci..

[76] Song Q., Shen J., Yang Y., Wang J., Yang Y., Sun J., Jiang B., Liao Z. (2020). Effect of temperature on the synthesis of sodalite by crystal transition process. Microporous Mesoporous Mater..

[77] Hayashi T., Shiga H., Sadakata M., Okuboa T. (1998). Hydrothermal growth of millimeter-sized aluminosilicate sodalite single crystals in noble metal capsules. J. Mater. Res..

[78] Klunk M., Dasgupta S., Das M., Cunha M.G., Wander P.R. (2019). Synthesis of sodalite zeolite and adsorption study of crystal violet dye. ECS J. Solid State Sci. Technol..

[79] Ding L., Yang H., Rahimi P., Omotoso O., Friesen W., Fairbridge C., Shi Y., Ng S. (2010). Solid transformation of zeolite NaA to sodalite. Microporous Mesoporous Mater..

[80] Greer H., Wheatley P.S., Ashbrook S.E., Morris R.E., Zhou W. (2009). Early stage reversed crystal growth of zeolite A and its phase transformation to sodalite. J. Am. Chem. Soc..

[81] Depmeier W. (2005). The sodalite family—A simple but versatile framework structure. Rev. Mineral. Geochem..

[82] Baur W.H., Fischer R.X. (2008). ZeoBase, a Databank for Zeolitetype Crystal Structures.

[83] Fischer R.X., Baur W.H. (2009). Symmetry relationships of sodalite (SOD)-type crystal structures. Z. Krist..

[84] Baerlocher C., McCusker L.B. (2007). Atlas of Zeolite Framework Types.

[85] Furukawa H., Cordova K.E., O’Keeffe M., Yaghi O.M. (2013). The chemistry and Applications of Metal-Organic Frameworks. Science.

[86] Park K.S., Ni Z., Côté A.P., Choi J.Y., Huang R., Uribe-Romo F.J., Chae H.K., O’Keeffe M., Yaghi O.M. (2006). Exceptional chemical and thermal stability of zeolitic imidazolate frameworks. Proc. Natl. Acad. Sci. USA.

[87] Khay I., Chaplais G., Nouali H., Ortiz G., Marichal C., Patarin J. (2016). Assessment of the energetic performances of various ZIFs with SOD or RHO topology using high pressure water intrusion–extrusion experiments. Dalton Trans..

[88] Zheng B., Zhu Y., Fu F., Wang L.L., Wang J., Du H. (2017). Theoretical prediction of the mechanical properties of zeolitic imidazolate frameworks (ZIFs). RSC Adv..

[89] Andres-Garcia E., López-Cabrelles J., Oar-Arteta L., Roldan-Martinez B., Cano-Padilla M., Gascon J., Espallargas G.M., Kapteijn F. (2019). Cation influence in adsorptive propane/propylene separation in ZIF-8 (SOD) topology. Chem. Eng. J..

[90] Bose R., Ethiraj J., Sridhar P., Varghese J.J., Kaisare N.S., Selvam P. (2020). Adsorption of hydrogen and carbon dioxide in zeolitic imidazolate framework structure with SOD topology: Experimental and modelling studies. Adsorption.

[91] Chen C., Kim J., Yang D.-A., Ahn W.-S. (2011). Carbon dioxide adsorption over zeolite-like metal organic frameworks (ZMOFs) having a sod topology: Structure and ion-exchange effect. Chem. Eng. J..

[92] Li M.-Y., Wang F., Zhang J. (2020). Zeolitic Tetrazolate–Imidazolate Frameworks with SOD Topology for Room Temperature Fixation of CO 2 to Cyclic Carbonates. Cryst. Growth Des..

[93] Huh S., Kwon T.-H., Park N., Kim S.-J., Kim Y. (2009). Nanoporous In-MOF with multiple one-dimensional pores. Chem. Commun..

[94] Sun L., Xing H., Liang Z., Yu J., Xu R. (2013). A 4 + 4 strategy for synthesis of zeolitic metal–organic frameworks: An indium-MOF with SOD topology as a light-harvesting antenna. Chem. Commun..

[95] Alsadun N., Mouchaham G., Guillerm V., Czaban-Jóźwiak J., Shkurenko A., Jiang H., Bhatt P.M., Parvatkar P., Eddaoudi M. (2020). Introducing a Cantellation Strategy for the Design of Mesoporous Zeolite-like Metal–Organic Frameworks: Zr-sod-ZMOFs as a Case Study. J. Am. Chem. Soc..

[96] Lee J.H., Jeoung S., Chung Y.G., Moon H.R. (2019). Elucidation of flexible metal-organic frameworks: Research progresses and recent developments. Coord. Chem. Rev..

[97] Gao M., Huang R.-K., Zheng B., Wang P., Shi Q., Zhang W.-X., Dong J. (2022). Large breathing effect in ZIF-65(Zn) with expansion and contraction of the SOD cage. Nat. Commun..

[98] Trojan I.A., Semenok D.V., Ivanova A.G., Kvashnin A.G., Zhou D., Sadakov A.V., Sobolevsky O.A., Pudalov V.M., Lyubutin I.S., Oganov A.R. (2022). High-temperature superconductivity in hydrides. Usp. Fiz. Nauk..

[99] Semenok D.V., Kvashnin A.G., Ivanova A.G., Svitlyk V., Fominski V.Y., Sadakov A.V., Sobolevskiy O.A., Pudalov V.M., Troyan I.A., Oganov A.R. (2020). Superconductivity at 161 K in thorium hydride ThH10: Synthesis and properties. Mater. Today.

[100] Semenok D.V., Kruglov I.A., Savkin I.A., Kvashnin A.G., Oganov A.R. (2020). On Distribution of Superconductivity in Metal Hydrides. Curr. Opin. Solid State Mater. Sci..

[101] Liu H., Naumov I.I., Hoffmann R., Ashcroft N.W., Hemley R.J. (2017). Potential high- T c superconducting lanthanum and yttrium hydrides at high pressure. Proc. Natl. Acad. Sci. USA.

[102] Heil C., di Cataldo S., Bachelet G.B., Boeri L. (2019). Superconductivity in sodalite-like yttrium hydride clathrates. Phys. Rev. B.

[103] Semenok D.V., Troyan I.A., Ivanova A.G., Kvashnin A.G., Kruglov I.A., Hanfland M., Sadakov A.V., Sobolevskiy O.A., Pervakov K.S., Lyubutin I.S. (2021). Superconductivity at 253 K in lanthanum–yttrium ternary hydrides. Mater. Today.

[104] Du M., Song H., Zhang Z., Duan D., Cui T. (2022). Room-Temperature Superconductivity in Yb/Lu Substituted Clathrate Hexahydrides under Moderate Pressure. Research.

[105] Tsuppayakorn-aek P., Phaisangittisakul N., Ahuja R., Bovornratanaraks T. (2021). High-temperature superconductor of sodalite-like clathrate hafnium hexahydride. Sci. Rep..

[106] Ma L., Wang K., Xie Y., Yang X., Wang Y., Zhou M., Liu H., Yu X., Zhao Y., Wang H. (2022). High-temperature superconducting phase in clathrate calcium hydride CaH_6_ up to 215 K at a pressure of 172 GPa. Phys. Rev. Lett..

[107] Karttunen A.J., Fässler T.F., Linnolahti M., Pakkanen T.A. (2011). Structural Principles of Semiconducting Group 14 Clathrate Frameworks. Inorg. Chem..

[108] Zhu L., Borstad G.M., Liu H., Guńka P.A., Guerette M., Dolyniuk J.-A., Meng Y., Greenberg E., Prakapenka V.B., Chaloux B.L. (2020). Carbon-boron clathrates as a new class of sp 3 -bonded framework materials. Sci. Adv..

[109] Shen J., Xie T., Zhang L., Wang P., Fang Z. (2020). Si_2_Ge: A New VII-Type Clathrate with Ultralow Thermal Conductivity and High Thermoelectric Property. Sci. Rep..

[110] Gatta G.D. (2008). Does porous mean soft? On the elastic behaviour and structural evolution of zeolites under pressure. Z. Krist. Cryst. Mater..

[111] Bau W.H., Fischer R.X. (2023). The flexibility of the T–X–T hinges between the coordination tetrahedra in various zeolitic frameworks: An empirical structural study. Mineral. Petrol..

[112] Li Z., Nevitt M.V., Ghose S. (1989). Elastic constants of sodalite Na_4_Al_3_Si_3_O_12_Cl. Appl. Phys. Lett..

[113] Ulian G., Valdrè G. (2022). Structural and Elastic Behaviour of Sodalite Na_8_(Al_6_Si_6_O_24_)Cl_2_ at High-Pressure by First-Principle Simulations. Minerals.

[114] Hazen R.M., Sharp Z.D. (1988). Compressibility of sodalite and scapolite. Am. Mineral..

[115] Elhachemi K., Khellafi H., Bendouba M., Djebli A. (2024). Quantification of Young's modulus of kaolin, sodalite and nanocomposite based polycaprolactone/sodalite using atomic force microscopy. Mater. Res. Express.

[116] Hargis C.W., Moon J., Lothenbach B., Winnefeld F., Wenk H.-R., Monteiro P.J.M. (2014). Calcium sulfoaluminate sodalite (Ca_4_Al_6_O_12_SO_4_) crystal structure evaluation and bulk modulus determination. J. Am. Ceram. Soc..

[117] Fütterer K., Depmeier W., Altorfer F., Behrens P., Felsche J. (1994). Compression mechanism in trioxane silica sodalite, [Si_12_O_24_]·2C_3_H_6_O_3_. Z. Krist. Cryst. Mater..

[118] Dann S.E., Weller M.T., Rainford B.D., Adroja D.T. (1997). Synthesis, structure, optical properties, and magnetism of the manganese chalcogenide ceryllosilicate and beryllogermanate sodalites. Inorg. Chem..

[119] Harrison W.T.A., Gier T.E., Stucky G.D. (1994). Two lithium chloroberyllo(phosphate/arsenate) sodalites: Li_4_Cl(BePO_4_)_3_ and Li_4_Cl(BeAsO_4_)_3_. Acta Crystallogr. Sect. C Cryst. Struct. Commun..

[120] Johnson G.M., Weller M.T. (1997). Synthesis and characterisation of gallium and germanium containing sodalites. Stud. Surf. Sci. Catal..

[121] Moran K.L., Gier T.E., Harrison W.T.A., Stucky G.D., Eckert H., Eichele K., Wasylishen R.E. (1993). Synthesis and characterization of mixed ZnSe/GaP semiconductor species included in the sodalite structure. J. Am. Chem. Soc..

[122] Moon D.J., Lim W.T. (2020). Minireview of pentatomic cations in sodalite cavities. J. Porous Mater..

[123] Kim S.H., Ha S.G., Heo N.H., Seff K. (2011). A crystallographic study of the decomposition of NO in fully indium exchanged zeolite Y. In^+^, In^3+^, and In^3+^–NO_3_^–^ complexes with facially coordinating nitrate ions are in supercages. Distorted cubic In_4_O_4_^4+^ clusters fill sodalite cavities. J. Phys. Chem. C.

[124] Frank S.M., Barber T.L., Lambregts M.J. (2005). Powder diffraction of sodalite in a multiphase ceramic used to immobilize radioactive waste. Powder Diffr..

[125] Riley B.J., Peterson J.A., Chong S., Vienna J.D. (2021). Influence of ion site occupancies on the unit cell parameters, specific volumes, and densities of *M*_8_(AlSiO_4_)_6_*X*_2_ sodalites where *M* = Li, Na, K, Rb, and Ag and *X* = Cl, Br, and I. Phys. Chem. Miner..

[126] Shannon R.D. (1976). Revised effective ionic radii and systematic studies of interatomic distances in halides and chalcogenides. Acta Cryst A.

[127] Taylor D., Henderson C.M.B. (1978). A computer model for the cubic sodalite structure. Phys. Chem. Miner..

[128] Borhade A.V., Dholi A.G., Tope D.P., Wakchaure S.G. (2012). Solvithermal synthesis and crystal structure of aluminogermanate halide sodalites using organic solvent. Indian J. Pure Appl. Phys..

[129] Shchipalkina N.V., Zubkova N.N., Kotelnikov A.R., Koshlyakova N.N., Pekov I.V., Ksenofontov D.A., Britvin S.N. (2021). Crystal chemistry and Raman spectroscopy of two synthetic sodalite-type aluminosilicates with [MoO_4_]^2−^ and [WO_4_]^2−^ groups. Phys. Chem. Miner..

[130] Mattigod S.V., McGrail B.P., McCready D.E., Wang L.-Q., Parker K.E., Young J.S. (2006). Synthesis and structure of perrhenate sodalite. Microporous Mesoporous Mater..

[131] Gramenitskii E.N., Kotel’nikov A.R., Shchekina T.I., Yakubovich O.V., Devyatova V.N., Zubkov E.S., Suk N.I., Vigasina M.F., Kotel’nikova Z.A. (2018). Composition, structure, and conditions of formation of fluorine-bearing sodalite: Experimental evidence. Geochem. Int..

[132] Yakubovich O.V., Kotel’nikov A.R., Shchekina T.I., Gramenitskiy E.N., Zubkov E.S. (2011). New representative in the dodalite dtructure type with extraframework anions [AlF_6_]^3−^. Crystallogr. Rep..

[133] Van Peteghem J.K., Burley B.J. (1963). Studies on solid solution between sodalite, nosean and haüyne. Can. Mineral..

[134] Ito T., Nakajima Y., Morimoto N., Sadanaga R. (1966). On the polysynthetic structure of haüyne. Acta Crystallogr..

[135] Taylor D. (1967). The sodalite group of minerals, Contrib. Mineral. Petrol..

[136] Schulz H. (1970). Struktur- and Überstrukturuntersuchungen an Nosean-Einkristallen. Z. Kristallogr..

[137] Tsuchiya N., Takéuchi Y. (1985). Fine texture of hauyne having modulated structure. Z. Kristallogr..

[138] Hassan I., Buseck P.R. (1989). Cluster ordering and antiphase domain boundaries in haüyne. Can. Mineral..

[139] Xu H., Veblen D.R. (1995). Transmission electron microscopy study of optically anisotropic and isotropic haüyne. Am. Mineral..

[140] Sapozhnikov A.N. (1992). Modulated structure of lazurite from deposits in southwestern Pamir. Sov. Phys. Crystallogr..

[141] Sapozhnikov N., Ivanov V.G., Levitsky V.I., Piskunova L.F. (1993). Structural-mineralogical peculiarities of lazurite from the south-western Pamir. Zap. Vses. Mineral. Obsh..

[142] Evsyunin V.G., Sapozhnikov A.N., Rastsvetaeva R.K., Kashaev A.A. (1998). Modulated structure of orthorhombic lazurite. Crystallogr. Rep..

[143] Rastsvetaeva R.K., Bolotina N.B., Sapozhnikov A.N., Kashaev A.A., Schoenleber A., Chapuis G. (2002). Average structure of cubic lazurite with a three-dimensional incommensurate modulation. Crystallogr. Rep..

[144] Bolotina N.B., Rastsvetaeva R.K., Sapozhnikov A.N., Kashaev A.A., Shönleber A., Chapuis G. (2003). Incommensurately modulated structure of isotropic lazurite as a product of twinning of two-dimensionally modulated domains. Crystallogr. Rep..

[145] Bolotina N.B., Rastsvetaeva R.K., Chapuis G., Schönleber A., Sapozhnikov A.N., Kashaev A.A. (2004). On the symmetry of optically isotropic modulated lazurites from the Baikal region. Ferroelectrics.

[146] Bolotina N.B., Rastsvetaeva R.K., Sapozhnikov A.N. (2006). Average structure of incommensurately modulated monoclinic lazurite. Crystallogr. Rep..

[147] Bolotina N.B. (2006). Isotropic lazurite: A cubic single crystal with an incommensurate three-dimensional modulation of the structure. Crystallogr. Rep..

[148] Bolotina N. (2007). Forms and origin of structure modulation in lazurites. Philos. Mag..

[149] Tauson V.L., Sapozhnikov A.N., Kaneva E.V., Lipko S.V. (2014). Reversion of incommensurate modulation in cubic lazurite: Example of reversible forced equilibrium?. Nat. Resour..

[150] Demyanets L.N. (1997). The growth of germanium bromide sodalite crystals under hydrothermal conditions. Crystallogr. Rep..

[151] Weippert V., Chau T., Witthaut K., Eisenburger L., Johrendt D. (2021). BaGe_8_As_14_: A semiconducting sodalite-type compound. Chem. Commun..

[152] Weller M.T., Haworth K.E. (1991). Synthesis and thermal decomposition of sodalites Na_8_[SiAlO_4_]_6_·(XO_4_)_2_, X = CI, Mn. J. Chem. Soc. Chem. Commun..

[153] Vidal L., Paillaud J., Gabelica Z. (1998). A novel monoclinic AlPO_4_-sodalite formed in the presence of dimethylformamide as template and solvent. Microporous Mesoporous Mater..

[154] Depmeier W., Yamamoto A. (1991). Powder profile refinement of a commensurately modulated aluminate sodalite. Mater. Sci. Forum.

[155] Sapozhnikov A.N., Tauson V.L., Matveeva L.N. (2001). Discrete change of the modulation wave in the structure of cubic lazurite from the Baikal Lake area during its annealing. Zap. Vseross. Mineral. Obsh..

[156] Petersen H., Robben L., Šehović M., Gesing T.M. (2017). Synthesis, temperature-dependent X-ray diffraction and Raman spectroscopic characterization of the sodalite to nosean phase transformation of |Na_7.7(1)_(MnO_4_)_1.7(2)_(H_2_O)_0.8(2)_|[AlSiO_4_]_6_. Microporous Mesoporous Mater..

[157] Bolotina N.B., Chukanov N.V., Schäfer C. (2022). Growth twins and deformation twins of sodalite-type microporous compounds. Microporous Mesoporous Mater..

[158] Borhade A.V., Dholi A.G. (2013). Synthesis and crystal structure of chlorate-enclathrated in aluminogermanate sodalite Na_8_[AlGeO_4_]_6_(ClO_3_)_2_. Mater. Sci. Pol..

[159] Yoshimori T., Asano Y., Toriumi Y., Shiota T. (1978). Investigation on the drying and decomposition of sodium oxalate. Talanta.

[160] Robben L. (2020). Cage reactions in sodalites—A phenomenological approach using cellular automata. Microporous Mesoporous Mater..

[161] Borhade A.V., Dholi A.G., Kshirsagar T.A. (2020). Synthesis and characterization of a new aluminogermanate thiocyanate aluminogermanate sodalite Na_8_[AlGeO_4_]_6_(SCN)_2_. Russ. J. Phys. Chem. A.

[162] Borhade A.V., Wakchaure S.G., Dholi A.G., Kshirsagar T.A. (2017). Hydrothermal synthesis, characterization, and thermal properties of alumino silicate azide sodalite, Na_8_[AlSiO_4_]_6_(N_3_)_2_. Russ. J. Phys. Chem. A.

[163] Herbstein F.H., Ron G., Weissman A. (1971). The thermal decomposition of potassium permanganate and related substances. Part I. Chemical aspects. J. Chem. Soc. A.

[164] Šehović M., Robben L., Gesing T.M. (2015). Carbon dioxide uptake in nitrite-sodalite: Reaction kinetics and template ordering of the carbonate-nosean formation. Z. Krist. Cryst. Mater..

[165] Gobeltz-Hautecoeur N., Demortier A., Lede B., Lelieur J.P., Duhayon C. (2002). Occupancy of the sodalite cages in the blue ultramarine pigments. Inorg. Chem..

[166] Pokrovski G.S., Dubrovinsky L.S. (2011). The S_3_^−^ ion is stable in geological fluids at elevated temperatures and pressures. Science.

[167] Pokrovski G.S., Dubessy J. (2015). Stability and abundance of the trisulfur radical ion S_3_^−^ in hydrothermal fluids. Earth Planet. Sci. Lett..

[168] Caggiani M.C., Mangone A., Acquafredda P. (2022). Blue coloured haüyne from Mt. Vulture (Italy) volcanic rocks: SEM-EDS and Raman investigation of natural and heated crystals. J. Raman Spectrosc..

[169] Ballirano O., Maras A. (2005). Crystal chemical and structural characterization of an unusual CO_3_-bearing sodalite-group mineral. Eur. J. Mineral..

[170] Rastsvetaeva R.K., Ivanova A.G., Chukanov N.V., Verin I.A. (2007). Crystal structure of alloriite. Dokl. Earth Sci..

[171] Chukanov N.V., Zubkova N.V., Pekov I.V., Giester G., Pushcharovsky D.Y. (2021). Sulfite analogue of alloriite from Sacrofano, Latium, Italy: Crystal chemistry and specific features of genesis. Geol. Ore Depos..

[172] Chukanov N.V., Zubkova N.V., Varlamov D.A., Pekov I.V., Belakovskiy D.I., Britvin S.N., Van K.V., Ermolaeva V.N., Vozchikova S.A., Pushcharovsky D.Y. (2022). Steudelite, (Na_3_)[(K,Na)_17_Ca_7_]Ca_4_(Al_24_Si_24_O_96_)(SO_3_)_6_F_6_·4H_2_O, a new cancrinite-group mineral with afghanite-type framework topology. Phys. Chem. Miner..

[173] Zubkova N.V., Chukanov N.V., Varlamov D.A., Vigasina M.F., Pekov I.V., Ksenofontov D.A., Pushcharovsky D.Y. (2022). Sulfite-bearing analogue of marinellite. Zap. Vseross. Mineral. Obsh..

[174] Buhl J.-C. (1991). Hydrothermal synthesis and characterization of nitrite sodalite single crystals. J. Cryst. Growth.

[175] Matussek T., Buhl J.-C. (2009). Stability and thermal behaviour of BH_4_-anions inside pores of sodium-potassium-tetrahydroborate aluminosilicate sodalite (Na_1–x_K_x_)_8_[AlSiO_4_]_6_(BH_4_)_2_; x~0.5. React. Kinet. Catal. Lett..

[176] Gilbert M.R. (2015). Pressureless sintering of sodalite waste-forms for the immobilization of pyroprocessing wastes. Mater. Res. Soc. Symp. Proc..

[177] Richmann M.K., Reed D.T., Kropf A.J., Aase S.B., Lewis M.A. (2001). EXAFS/XANES studies of plutonium-loaded sodalite/glass waste forms. J. Nucl. Mater..

[178] Pierce E.M., Lilova K., Missimer D.M., Lukens W.W., Wu L., Fitts J., Rawn C., Huq A., Leonard D.N., Eskelsen J.R. (2017). Structure and thermochemistry of perrhenate sodalite and mixed guest perrhenate/pertechnetate sodalite. Environ. Sci. Technol..

[179] Anenburg M., Le Losq C. (2019). Perrhenate sodalite growth from alkali silicate melts by noble metal catalysis. SN Appl. Sci..

[180] Lilova K., Pierce E.M., Wu L., Jubb A.M., Subramani T., Navrotsky A. (2020). Energetics of salt-bearing sodalites, Na_8_Al_6_Si_6_O_24_X_2_ (X = SO_4_, ReO_4_, Cl, I): A treatment option for pertechnetate-enriched nuclear waste streams. ACS Earth Space Chem..

[181] Dickson J.O., Harsh J.B., Flury M., Pierce E.M. (2015). Immobilization and exchange of perrhenate in sodalite and cancrinite. Microporous Mesoporous Mater..

[182] Dickson J.O., Harsh J.B., Flury M., Lukens W.W., Pierce E.M. (2014). Competitive incorporation of perrhenate and nitrate into sodalite. Environ. Sci. Technol..

[183] Petersen H., Robben L., Gesing T.M. (2020). On the nature of the phase transitions of aluminosilicate perrhenate sodalite. Z. Krist. Cryst. Mater..

[184] Luksic S.A., Riley B.J., Parker K.E., Hrma P. (2016). Sodalite as a vehicle to increase Re retention in waste glass stimulant during vitrification. J. Nucl. Mater..

[185] Dickson J.O., Harsh J.B., Lukens W.W., Pierce E.M. (2015). Perrhenate incorporation into binary mixed sodalites: The role of anion size and implications for technetium-99 sequestration. Chem. Geol..

[186] Hirabayashi D., Tanada Y., Sugiyama T., Enokida Y. (2012). Low-temperature conversion of spent adsorbent to iodine sodalite by a mechanochemical route. J. Am. Inst. Chem. Eng..

[187] Sheppard G.P., Hriljac J.A., Maddrell E.R., Hyatt N.C. (2006). Silver zeolites: Iodide occlusion and conversion to sodalite—A potential ^129^I waste form?. Mat. Res. Soc. Symp. Proc..

[188] Vance E.R., Gregg D.J., Grant C., Stopic A., Maddrell E.R. (2016). Silver iodide sodalite for ^129^I immobilization. J. Nucl. Mater..

[189] Maddrell E., Gandy A., Stennett M. (2014). The durability of iodide sodalite. J. Nucl. Mater..

[190] Maddrell E.R., Vance E.R., Grant C., Aly Z., Stopic A., Palmer T., Harrison J., Gregg D.J. (2019). Silver iodide sodalite—Wasteform/Hip canister interactions and aqueous durability. J. Nucl. Mater..

[191] Lepry W.C., Riley B.J., Crum J.V., Rodriguez C.P., Pierce D.A. (2013). Solution-based approaches for making high-density sodalite waste forms to immobilize spent electrochemical salts. J. Nucl. Mater..

[192] De Angelis G., Nannicini R., Martini F., Mazzocchia C., Modica G. (2008). Different methods to synthesize sodalite, as a matrix for conditioning chloride spent salts from pyroprocesses. Radiochim. Acta.

[193] Giacobbo F., da Ros M., Macerata E., Mariani M., Giola M., de Angelis G., Capone M., Fedeli C. (2018). An experimental study on Sodalite and SAP matrices for immobilization of spent chloride salt waste. J. Nucl. Mater..

[194] Capone M., Fedeli C., de Angelis G., da Ros M., Giacobbo F., Giola M., Macerata E., Mariani M. (2017). A study on sodalite pellets as matrix for spent chloride salts confinement. J. Mater. Res. Soc..

[195] Riley B.J., Lepry W.C., Crum J.V. (2016). Solution-Derived Sodalite Made with Si- and Ge-Ethoxide Precursors for Immobilizing Electrorefiner Salt. J. Nucl. Mater..

[196] Riley B.J., Vienna J.D., Frank S.M., Kroll J.O., Peterson J.A., Canfield N.L., Zhu Z., Zhang J., Kruska K., Schreiber D.K. (2017). Glass binder development for a glass-bonded sodalite ceramic waste form. J. Nucl. Mater..

[197] Borhade A.V., Dholi A.G., Wakchaure G., Tope D.R. (2012). Chemical modification of coal fly ash into iodate sodalite and its use for the removal of Cd^2+^, Pb^2+^, and Zn^2+^ from their aqueous solutions. Desalination Water Treat..

[198] Esfandiana H., Parvinia M., Khoshandama B., Samadi-Maybodi A. (2015). Removal of diazinon from aqueous solutions in batch systems using Cu-modified sodalite zeolite: An application of response surface methodology. Int. J. Eng. Trans. Appl..

[199] Canfield G.M., Bizimis M., Latturner S.E. (2007). Sodalite ion exchange in polyethylene oxide oligomer solvents. J. Mater. Chem..

[200] van den Berg A.W.C. (2006). Opportunities and Limitations of Hydrogen Storage in Zeolitic Clathrates. Ph.D. Thesis.

[201] van den Berg A.W.C., Bromley S.T., Flikkema E., Wojdel J., Maschmeyer T., Jansen J.C. (2004). Molecular-dynamics analysis of the diffusion of molecular hydrogen in all-silica sodalite. J. Chem. Phys..

[202] van den Berg A.W.C., Bromley S.T., Flikkema E., Jansen J.C. (2004). Effect of cation distribution on self-diffusion of molecular hydrogen in Na_3_Al_3_Si_3_O_12_ sodalite: A molecular dynamics study. J. Chem. Phys..

[203] van den Berg A.W.C., Bromley S.T., Jansen J.C. (2005). Thermodynamic limits on hydrogen storage in sodalite framework materials: A molecular mechanics investigation. Microporous Mesoporous Mater..

[204] Weitkamp J., Ernst S., Cubero F., Wester F. (1997). Nitrido-sodalite Zn_6_[P_12_N_24_] as a material for reversible hydrogen encapsulation. Adv. Mater..

[205] Dincă M., Dailly A., Tsay C., Long J.R. (2008). Expanded sodalite-type metal–organic frameworks: Increased stability and H_2_ adsorption through ligand-directed catenation. Inorg. Chem..

[206] Dincă M., Dailly A., Liu Y., Brown C.M., Neumann D.A., Long J.R. (2006). Hydrogen storage in microporous metal-organic framework with exposed Mn^2+^ coordination sites. J. Am. Chem. Soc..

[207] Luo J.H., Xu H.W., Liu Y., Zhao Y.S., Daemen L.L., Brown C., Timofeeva T.V., Ma S.Q., Zhou H.C. (2008). Hydrogen adsorption in a highly stable porous rare-earth metal-organic framework: Sorption properties and neutron diffraction studies. J. Am. Chem. Soc..

[208] Prasad T.K., Hong D.H., Suh M.P. (2010). High gas sorption and metal-ion exchange of microporous metal-organic frameworks with incorporated imide groups. Chem. Eur. J..

[209] Sumida K. (2012). Design and Synthesis of Metal-Organic Frameworks for Hydrogen Storage and Carbon Dioxide Capture. Ph.D. Thesis.

[210] Yang S.J., Im J.H., Nishihara H., Jung H., Lee K., Kyotani T., Park C.R. (2012). General relationship between hydrogen adsorption capacities at 77 and 298 K and pore characteristics of the porous adsorbents. J. Phys. Chem. C.

[211] Pillai Z.S., Prasad P.P.M.H., Latheef A.S.A., Pillai A., Chandrasekhar A., Jacob G. (2023). Synthesis and fine tuning of MOF for hydrogen storage. Recent Trends in the Application of Metal-Organic Frameworks.

[212] Leonard A.D., Hudson J.L., Fan H., Booker R., Simpson L.J., O’Neill K.J., Parilla P.A., Heben M.J., Pasquali M., Kittrell C. (2009). Nanoengineered carbon scaffolds for hydrogen storage. J. Am. Chem. Soc..

[213] Basova T.V., Polyakov M.S. (2020). Hybrid materials based on carbon nanotubes and polyaromatic molecules: Methods of functionalization and sensor properties. Macroheterocycles.

[214] Gong Y.-N., Meng M., Zhong D.-C., Huang Y.-L., Jiang L., Lu T.-B. (2012). Counter-cation modulation of hydrogen and methane storage in a sodalite-type porous metal–organic framework. Chem. Commun..

[215] Sumida K., Horike S., Kaye S.S., Herm Z.R., Queen W.L., Brown C.M., Grandjean F., Long G.J., Dailly A., Long J.R. (2010). Hydrogenstorage and carbon dioxide capture in an iron-based sodalite-type metal–organic framework (Fe-BTT) discovered *via* high-throughput methods. Chem. Sci..

[216] Gygi D., Bloch E.D., Mason J.A., Hudson M.R., Gonzalez M.I., Siegelman R.L., Darwish T.A., Queen W.L., Brown C.M., Long J.R. (2016). Hydrogen Storage in the Expanded Pore Metal–Organic Frameworks M_2_(dobpdc) (M = Mg, Mn, Fe, Co, Ni, Zn). Chem. Mater..

[217] Buhl J.-C., Schomborg L., Rüscher C.H. (2010). Tetrahydroborate sodalite nanocrystals: Low temperature synthesis and thermally controlled intra-cage reactions for hydrogen release of nano- and micro crystals. Microporous Mesoporous Mater..

[218] Buhl J.-C., Rüscher C.H., Schomborg L., Stemme F. (2010). Nanocrystalline NaBH_4_-enclathrated zeolite SOD: A model for the improvement of safeness and reactivity of boron hydride based hydrogen storage systems. Clean Technol..

[219] Buhl J.-C., Gesing T.M., Rüscher C.H. (2005). Synthesis, crystal structure and thermal stability of tetrahydroborate sodalite Na_8_[AlSiO_4_]_6_(BH_4_)_2_. Microporous Mesoporous Mater..

[220] Rüscher C.H., Stemme F., Schomborg L., Buhl J.-C. (2010). Low-temperature hydrogen release from borontetrahydride-sodalite and its reloading: Observations in in-situ and ex-situ TIR experiments. Ceram. Environ. Energy Appl..

[221] Kumar S., Jain A., Miyaoka H., Ichikawa T., Kojima Y. (2017). Study on the thermal decomposition of NaBH_4_ catalyzed by ZrCl_4_. Int. J. Hydrogen Energy.

[222] Martelli P., Caputo R., Remhof A., Mauron P., Borgschulte A., Züttel A. (2010). Stability and decomposition of NaBH_4_. J. Phys. Chem..

[223] Buhl J.-C., Stemme F., Poltz I. (2009). Hydrothermal stability of NaBH_4_ enclathrated sodalites with aluminosilicate and gallosilicate framework. Microporous Mesoporous Mater..

[224] Buhl J.-C. (2017). NaBH_4_ Sodalites, synthesized by modified methods: (1) autothermal synthesis and (2) crossover reaction from gel to melt flow. Adv. Chem. Eng. Sci..

[225] Zheng Z., Guliants V.V., Misture S. (2009). Sodalites as ultramicroporous frameworks for hydrogen separation at elevated temperatures: Thermal stability, template removal, and hydrogen accessibility. J. Porous Mater..

[226] De Luca G., Pullumbi P., Barbieri G., Famà A.D., Bernardo P., Drioli E. (2004). Gusev and Suter calculation of the diffusion coefficients of light gases in silicalite-1 membrane and silica-sodalite zeolite. Sep. Purif. Technol..

[227] Yao J., Zhang L., Wang H. (2008). Synthesis of nanocrystalline sodalite with organic additives. Mater. Lett..

[228] Asgari M., Jawahery S., Bloch E.D., Hudson M.R., Flacau R., Vlaisavljevich B., Long J.R., Brown C.M., Queen W.L. (2018). An experimental and computational study of CO_2_ adsorption in the sodalite-type M-BTT (M = Cr, Mn, Fe, Cu) metal–organic frameworks featuring open metal sites. Chem. Sci..

[229] Eterigho-Ikelegbe O., Bada S., Daramola M.O., Falcon R. (2021). Synthesis of high purity hydroxy sodalite nanoparticles via poreplugging hydrothermal method for inorganic membrane development: Effect of synthesis variables on crystallinity, crystal size and morphology. Mater. Today Proc..

[230] Qin J.-S., Du D.-Y., Li W.-L., Zhang J.-P., Li S.-L., Su Z.-M., Wang X.-L., Xu Q., Shao K.-Z., Lan Y.-Q. (2012). N-rich zeolite-like metal–organic framework with sodalite topology: High CO_2_ uptake, selective gas adsorption and efficient drug delivery. Chem. Sci..

[231] Asgari M., Semino R., Schouwink P.A., Kochetygov I. (2020). Understanding how ligand functionalization influences CO_2_ and N_2_ adsorption in a sodalite metaloorganic framework. Chem Mater..

[232] Wang Y., Jiang Y., Hu S., Peng S., Xu C., Lu A. (2019). Dehydrated Na_6_[AlSiO_4_]_6_ sodalite as a promising SO_2_ sorbent material: A first principles thermodynamics prediction. J. Am. Ceram. Soc..

[233] Navarro J.A.R., Barea E., Salas J.M., Masciocchi N., Galli S., Sironi A., Ania C.O., Parra J.B. (2006). H_2_, N_2_, CO, and CO_2_ sorption properties of a series of robust sodalite-type microporous coordination polymers. Inorg. Chem..

[234] Fasolin S., Romano M., Boldrini S., Ferrario A., Fabrizio M., Armelao L., Barison S. (2019). Single-step process to produce alumina supported hydroxy-sodalite zeolite membranes. J. Mater Sci..

[235] Yang G., Guo H., Kang Z., Feng S., Zhao L., Mintova S. (2020). Sandwich-type H_2_/CO_2_ membranes comprising of graphene oxide and sodalite crystals with adjustable morphology and size. Microporous Mesoporous Mater..

[236] Eterigho-Ikelegbe O., Bada S.O., Daramola M.O. (2020). Preparation and evaluation of nanocomposite sodalite/α-Al_2_O_3_ tubular membranes for H_2_/CO_2_ separation. Membranes.

[237] Eden C.L., Daramola M.O. (2021). Evaluation of silica sodalite infused polysulfone mixed matrix membranes during H_2_/CO_2_ separation. Mater. Today Proc..

[238] Lee S.-R., Son Y.-H., Julbe A., Choy J.-H. (2006). Vacuum seeding and secondary growth route to sodalite membrane. Thin Solid Films.

[239] Julbe A., Motuzas J., Cazevielle F., Volle G., Guizard C. (2003). Synthesis of sodalite/αAl_2_O_3_ composite membranes by microwave heating. Sep. Purif. Technol..

[240] Guo H., Kong G., Yang G., Pang J., Kang Z., Feng S., Zhao L., Fan L., Zhu L., Vicente A. (2020). Cross-linking between sodalite nanoparticles and graphene oxide in composite membranes to trigger high gas permeance, selectivity, and stability in hydrogen separation. Angew. Chem. Int. Ed..

[241] Yang G., Guo H., Kang Z., Zhao L., Feng S., Jiao F., Mintova S. (2019). Green hydrogen separation from nitrogen by mixed-matrix membranes consisting of nanosized sodalite crystals. ChemSusChem.

[242] Xu X., Bao Y., Song C., Yang W., Liu J., Lin L. (2004). Microwave-assisted hydrothermal synthesis of hydroxy-sodalite zeolite membrane. Microporous Mesoporous Mater..

[243] van Niekerk A., Zaha J., Breytenbach J.C., Krieg H.M. (2007). Direct crystallisation of a hydroxy sodalite membrane without seeding using a conventional oven. J. Membr. Sci..

[244] Nabavi M.S., Mohammadi T., Kazemimoghadam M. (2014). Hydrothermal synthesis of hydroxysodalite zeolite membrane: Separation of H_2_/CH_4_. Ceram. Int..

[245] Khajavi S., Jansen J.C., Kapteijn F. (2009). Application of hydroxy sodalite films as novel water selective membranes. J. Membr. Sci..

[246] Khajavi S., Kapteijn F., Jansen J.C. (2007). Synthesis of thin defect-free hydroxy sodalite membranes: New candidate for activated water permeation. J. Membr. Sci..

[247] Bayati B., Babaluo A.A., Namini P.A. (2009). Synthesis and seeding time effect on the inter-crystalline structure of hydroxy-sodalite zeolite membranes by single gas (H_2_ and N_2_) permeation. Iran. J. Chem. Chem. Eng..

[248] Kalantari N., Vaezi M.J., Yadollahi M., Babaluo A.A., Bayati B., Kazemzadeh A. (2015). Synthesis of nanostructure hydroxy sodalite composite membranes via hydrothermal method: Support surface modification and synthesis method effects. Asia-Pac. J. Chem. Eng..

[249] Khajavi S., Jansen J.C., Kapteijn F. (2010). Production of ultra pure water by desalination of seawater using a hydroxyl sodalite membrane. J. Membr. Sci..

[250] Khajavi S., Jansen J.C., Kapteijn F. (2010). Performance of hydroxy sodalite membranes as absolute water selective materials under acidic and basic conditions. J. Membr. Sci..

[251] Wei X.-L., Pan W.-Y., Li X., Pan M., Huo C.-F., Yang R., Chao Z.-S. (2020). MCM-22 zeolite-induced synthesis of thin sodalite zeolite membranes. Chem. Mater..

[252] Workneh S., Shukla A. (2008). Synthesis of sodalite octahydrate zeolite-clay composite membrane and its use in separation of SDS. J. Membr. Sci..

[253] Jiang X., Zhang Y., Zhang Y. (2019). Desilication mechanism and kinetics of synthesized hydroxy-sodalite in high-alkali sodium aluminate solutions. React. Kinet. Mech. Catal..

[254] Ntshangase N.C., Sadare O.O., Daramola M.O. (2021). Effect of silica sodalite functionalization and PVA coating on performance of sodalite infused PSF membrane during treatment of acid mine drainage. Membranes.

[255] Li D., Zhu H.Y., Ratinac K.R., Ringer S.P., Wang H. (2009). Synthesis and characterization of sodalite–polyimide nanocomposite membranes. Microporous Mesoporous Mater..

[256] Yu H., Shen J., Li J., Sun X., Han W., Liu X., Wang L. (2014). Preparation, characterization and adsorption properties of sodalite pellets. Mater. Lett..

[257] Wiśniewska M., Fijałkowska G., Nosal A., Franus M., Panek R. (2019). Adsorption mechanism of poly(vinyl alcohol) on the surfaces of synthetic zeolites: Sodalite, Na-P1 and Na-A. Adsorption.

[258] Daramola M.O., Silinda B., Masondo S., Oluwasina O.O. (2015). Polyethersulphone-sodalite (PES-SOD) mixed-matrix membranes: Prospects for acid mine drainage (AMD) treatment. J. South. Afr. Inst. Min. Metall..

[259] Cano N.F., Ayta W.E.F., Watanabe S. (2010). The electronic and optical properties of sodalite (Na_8_Al_6_Si_6_O_24_Cl_2_) from first principles. Solid State Commun..

[260] Pan L., Liu W., Chen W., Yan K., Yang H., Yu J. (2016). Crystal structure and band gap studies of sodalite: Experimental and calculated results. J. Mol. Struct..

[261] Grajciar L. (2016). PbS clusters embedded in sodalite zeolite cavities of different compositions: Unraveling the structural evolution and optical properties using *ab initio* calculations. J. Phys. Chem. C.

[262] Gmelin C.G. (1828). Üeber die künstliche Darstellung einer dem Ultramarin ähnlichen Farbe. Naturwiss. Abhandl..

[263] Deer W.A., Howie R.A., Wise W.S., Zussman J. (2004). Rock-forming minerals. Volume 4B. Framework silicates: Silica minerals. Feldspathoids and the Zeolites.

[264] Tauson V.L., Goettlicher J., Sapozhnikov A.N., Mangold S., Lustenberg R.E. (2012). Sulfur speciation in lazurite-type minerals (Na,Ca)_8_[Al_6_Si_6_O_24_](SO_4_,S)_2_ and their annealing products: A comparative XPS and XAS study. Eur. J. Mineral..

[265] Hettmann K., Wenzel T., Marks M., Markl G. (2012). The sulfur speciation in S-bearing minerals: New constraints by a combination of electron microprobe analysis and DFT calculations with special reference to sodalite-group minerals. Am. Mineral..

[266] Paterson I. (2004). A Dictionary of Colour: A Lexicon of the Language of Colour.

[267] Eastaugh N., Walsh V., Chaplin T., Siddall R. (2004). Pigment Compendium—A Dictionary and Optical Microscopy of Historical Pigments.

[268] Eckert B., Steudel R. (2003). Molecular spectra of sulfur molecules and solid sulfur allotropes. Top. Curr. Chem..

[269] Steudel R., Chivers T. (2019). The role of polysulfide dianions and radical anions in the chemical, physical and biological sciences, including sulfur-based batteries. Chem. Soc. Rev..

[270] Platonov A.N., Tarashchan A.N., Belichenko V.P., Povarennikh A.S. (1971). Spectroscopic study of sulfide sulfur in some framework aluminosilicates. Const. Prop. Miner..

[271] Samoilovich M.I. (1971). An ESR study of sulfur-bearing radical ions in minerals. Geokhimiya.

[272] Evsyunin V.G., Sapozhnikov A.N., Kashaev A.A., Rastsvetaeva R.R. (1997). Crystal structure of triclinic lazurite. Crystallogr. Rep..

[273] Steudel R., Steudel R. (2003). Inorganic polysulfides S*_n_*^2−^ and radical anions S*_n_*^∙−^. Elemental Sulfur und Sulfur-Rich Compounds II. Topics in Current Chemistry.

[274] Climent-Pascual E., de Paz J.R., Rodri E., Suard E., Sa R. (2009). Synthesis and characterization of the ultramarine-type analog Na_8-x_[Si_6_Al_6_O_24_]·(S^–2^,S^–3^,CO_3_)_(1–2)_. Inorg. Chem..

[275] Fleet M.E., Liu X., Harmer S.L., Nesbitt H.W. (2005). Chemical state of sulfur in natural and synthetic lazurite by S *K*-edge XANES and X-ray photoelectron spectroscopy. Can. Mineral..

[276] Chivers T., Elder P.J.W. (2013). Ubiquitous trisulfur radical anion: Fundamentals and applications in materials science, electrochemistry, analytical chemistry and geochemistry. Chem. Soc. Rev..

[277] Rejmak P. (2020). Computational refinement of the puzzling red tetrasulfur chromophore in ultramarine pigments. Phys. Chem. Chem. Phys..

[278] Wong M.W., Steudel R. (2003). Structure and spectra of tetrasulfur S_4_—an *ab initio* MO study. Chem. Phys. Lett..

[279] Rolfe J. (1968). Emission spectra of S^2−^, Se^2−^, and SeS^−^ ions in KI Crystals. J. Chem. Phys..

[280] Hålenius U. (2011). Absorption of light by exchange coupled pairs of tetrahedrally coordinated divalent manganese in the helvite-genthelvite solid solution. Period. Mineral..

[281] Blumentritt F., Fritsch E. (2021). Photochromism and photochromic gems: A review and some new data (Part 1). J. Gemmol..

[282] Agamah C., Vuori S., Colinet P., Norrbo I., de Carvalho J.M., Key L., Nakamura O., Lindblom J., van Goethem L., Emmermann A. (2020). Hackmanite—The natural glow-in-the-dark material. Chem. Mater..

[283] Jensen A., Petersen O.V. (1982). Tugtupite: A gemstone from Greenland. Gems Gemmol..

[284] Warner T.E., Andersen J.H. (2012). The effects of sulfur intercalation on the optical properties of artificial ‘hackmanite’, Na_8_[Al_6_Si_6_O_24_]Cl_1.8_S_0.1_; ‘sulfosodalite’, Na_8_[Al_6_Si_6_O_24_]S; and natural tugtupite, Na_8_[Be_2_Al_2_Si_8_O_24_](Cl,S)_2–δ_. Phys. Chem. Miner..

[285] Curutchet A., le Bahers T. (2017). Modeling the photochromism of S-doped sodalites using DFT, TDDFT, and SAC-CI methods. Inorg. Chem..

[286] Colinet P., Gheeraert A., Curutchet A., le Bahers T. (2020). On the spectroscopic modeling of localized defects in sodalites by TD-DFT. J. Phys. Chem. C.

[287] Hassib A., Beckman O., Annersten H. (1977). Photochromic properties of natural sodalite. J. Phys. D Appl. Phys..

[288] Pizani P.S., Terrile M.C., Farach H.A., Poole C.P. (1985). Color centers in sodalite. Am. Mineral..

[289] Norrbo I., Gluchowski P., Hyppänen I., Laihinen T., Laukkanen P., Mäkelä J., Mamedov F., Santos H.S., Sinkkonen J., Tuomisto M. (2016). Mechanisms of tenebrescence and persistent luminescence in synthetic hackmanite Na_8_Al_6_Si_6_O_24_(Cl,S)_2_. ACS Appl. Mater. Interfaces.

[290] Goettlicher J., Kotelnikov A., Suk N., Kovalski A., Vitova T., Steininger R. (2013). Sulfur K X-ray absorption near edge structure spectroscopy on the photochrome sodalite variety hackmanite. Z. Kristallogr..

[291] Zahoransky T., Friis H., Marks M.A.W. (2016). Luminescence and tenebrescence of natural sodalites: A chemical and structural study. Phys. Chem. Miner..

[292] Sidike A., Sawuti A., Wang X.-M., Zhu H.-J., Kobayashi S., Kusachi I., Yamashita N. (2007). Fine structure in photoluminescence spectrum of S_2_^−^ center in sodalite. Phys. Chem. Miner..

[293] Gaft M., Panczer G., Nagli L., Yeates H. (2009). Laser-induced timeresolved luminescence of tugtupite, sodalite and hackmanite. Phys. Chem. Miner..

[294] Kaiheriman M., Maimaitinaisier A., Rehiman A., Sidike A. (2014). Photoluminescence properties of green and red luminescence from natural and heat-treated sodalite. Phys. Chem. Miner..

[295] Kirk R.J. (1955). The luminescence and tenebrescence of natural and synthetic sodalite. Am. Miner..

[296] Ballentyne D.W.G., Bye K.L. (1970). The nature of photochromism in chlorosodalites from optical data. J. Phys. D Appl. Phys..

[297] Warner T.E. (2011). Artificial Hackmanite Na_8_[Al_6_Si_6_O_24_]Cl_1.8_S_0.1_ by a Structure-Conversion Method with Annealing under a Reducing Atmosphere, Synthesis, Properties and Mineralogy of Important Inorganic Materials.

[298] Taylor M.J., Marshall D.J., Forrester P.A., McLaughlan S.D. (1970). Colour centres in sodalites and their use in storage displays. Radio Electro Eng..

[299] Finch A.A., Friis H., Maghrabi M. (2016). Defects in sodalite-group minerals determined from X-ray-induced luminescence. Phys. Chem. Miner..

[300] Lezhnina M.M., Kynast U.H. (2005). NIR- and upconverted luminescence from rare-earth sodalites. Phys. Solid State.

[301] Lezhnina M., Laeri F., Benmouhadi L., Kynast U. (2006). Efficient near-infrared emission from sodalite derivatives. Adv. Mater..

[302] Kaiheriman M., Sidike A., Maimaitinasier A., Reheman A., Rouzi B. (2015). Photoluminescence properties of Tb^3+^-doped sodalite under VUV–UV light excitation. J. Lumin..

[303] Cano N.F., Blak A.R., Watanabe S. (2010). Correlation between electron paramagnetic resonance and thermoluminescence in natural sodalite. Phys. Chem. Miner..

[304] Chen C., Cai P., Qin L., Wang J., Bi S., Huang Y., Seo H.J. (2018). Luminescence properties of sodalite-type Zn_4_B_6_O_13_:Mn^2+^. J. Lumin..

[305] Khajavi S., Jansen J.C., Kapteijn F. (2010). Application of a sodalite membrane reactor in esterification—Coupling reaction and separation. Catal. Today.

[306] Aghaeinejad-Meybodi A., Mousavi S.M., Shahabi A.A., Kakroudi M.R. (2021). CFD modeling of methanol to light olefins in a sodalite membrane reactor using SAPO-34 catalyst with in situ steam removal. Comb. Chem. High Throughput Screen..

[307] Teimouri F., Khezri S.H., Azizian J. (2015). Hydroxy sodalite zeolite as a recyclable catalyst for the green synthesis of tetrahydrobenzo[*b*]pyrans via one-pot three-component condensation reaction. Iran. J. Catal..

[308] Bijsterbosch J.W., Das A., Kerkhof F. (1994). Clean technology in the production of epichlorohydrin. J. Clean. Prod..

[309] Lu Y., Wang R., Zhang J., Jin Q., Luo G. (2015). Evaluation of an improved epichlorohydrin synthesis from dichloropropanol using a microchemical system, Chinese. J. Chem. Eng..

[310] Shanbhag G.V., Choi M., Kim J., Ryoo R. (2009). Mesoporous sodalite: A novel, stable solid catalyst for base-catalyzed organic transformations. J. Catal..

[311] Hiyoshi N. (2012). Nanocrystalline sodalite: Preparation and application to epoxidation of.2-cyclohexen-1-one with hydrogen peroxide. Appl. Catal. A Gen..

[312] Sachse A., Galarneau A., di Renzo Francois Fajula F., Coq B. (2010). Synthesis of zeolite monoliths for flow continuous processes. The case of sodalite as a basic catalyst. Chem. Mater..

[313] Makgaba C.P., Daramola M.O. (2015). Transesterification of waste cooking oil to biodiesel over calcined hydroxy sodalite (HS) catalyst: A preliminary investigation. Proceedings of the 2015 International Conference on Sustainable Energy and Environmental Engineering.

[314] Rahimnejad M., Hassaninejad S.K., Pourali S.M. (2015). Preparation of template-free sodalite nanozeolite–chitosan-modified carbon paste electrode for electrocatalytic oxidation of ethanol. J. Iran. Chem. Soc..

[315] Abukhadra M.R., Adlii A., Jumah M.N.B., Othman S.I., Alruhaimi R.S., Salama Y.F., Allam A.A. (2021). Sustainable conversion of waste corn oil into biofuel over different forms of synthetic muscovite based K^+^/Na^+^ sodalite as basic catalysts; characterization and mechanism. Mater. Res. Express.

[316] Mani P., Devadas S., Gurusamy T., Karthik P.E., Ratheesh B.P., Ramanujam K., Mandal S. (2019). Sodalite-type Cu-based three-dimensional metal–organic framework for efficient oxygen reduction reaction. Chem. Asian J..

[317] Zhang L.-J., Han C.-Y., Dang Q.-Q., Wang Y.-H., Zhang X.-M. (2015). Solvent-free heterogeneous catalysis for cyanosilylation in a modified sodalite-type Cu(II)-MOF. Adv. R. Soc. Chem..

[318] Chen W., Maugé F., Gestel J.V., Nie H., Li D., Long X. (2013). Effect of modification of the alumina acidity on the properties of supported Mo and CoMo sulfide catalysts. J. Catal..

[319] Sun Y., Wang H., Prins R. (2010). Hydrodesulfurization with classic Co–MoS_2_ and Ni–MoS_2_/c-Al_2_O_3_ and new Pt–Pd on mesoporous zeolite catalysts. Catal. Today.

[320] Wang Y., Liu H., Wang S.Y., Luo M.F., Lu J.Q. (2014). Remarkable enhancement of dichloromethane oxidation over potassium-promoted Pt/Al_2_O_3_ catalysts. J. Catal..

[321] Zhang L., Fu W., Ke Q., Zhang S., Jin H., Hu J., Wang S., Tang T. (2012). Study of hydrodesulfurization of 4,6-DM-DBT over Pd supported on mesoporous USY zeolite. Appl. Catal. A.

[322] Xue D., Liu F., Gao H., Yang L., Wu Y., Xue W., Li Z.L.F. (2018). The synthesis, characterization and catalytic performance of platinum encapsulated mesopotous sodalite material. Rev. Roum. Chim..

[323] Gao H., Liu F., Xue D., Han R., Li F. (2018). Study on sulfur-tolerant benzene hydrogenation catalyst based on Pt-encapsulated sodalite zeolite. React. Kinet. Mech. Catal..

[324] Jiao F., Guo H., Chai Y., Awala H., Mintova S., Liu C. (2018). Synergy between a sulfur-tolerant Pt/Al_2_O_3_ sodalite core–shell catalyst and a CoMo/Al_2_O_3_ catalyst. J. Catal..

[325] Choi M., Lee D.-H., Na K., Yu B.-W., Ryoo R. (2009). High catalytic activity of palladium(II)-exchanged mesoporous sodalite and NaA zeolite for bulky aryl coupling reactions: Reusability under aerobic conditions. Angew. Chem. Int. Ed..

[326] Wang S., Zhao Z.-J., Chang X., Zhao J., Tian H., Yang C., Li M., Fu Q., Mu R., Gong J. (2019). Activation and spillover of hydrogen on sub-1 nm palladium nanoclusters confined within sodalite zeolite for the semi-hydrogenation of alkynes. Angew. Chem. Int. Ed..

[327] Ogura M., Morozumi K., Elangovan S.P., Tanada H., Ando H., Okubo T. (2008). Potassium-doped sodalite: A tectoaluminosilicate for the catalytic material towards continuous combustion of carbonaceous matters. App. Catal. B Environ..

[328] Manique M.C., Lacerda L.V., Alves A.K., Bergmann C.P. (2017). Biodiesel production using coal fly ash-derived sodalite as a heterogeneous catalyst. Fuel.

[329] Wang F., Liu Z.-S., Yang H., Tan Y.-X., Zhang J. (2011). Hybrid zeolitic imidazolate frameworks with catalytic active *T*O_4_ (*T* = Mo^6+^ or W^6+^) building blocks. Angew. Chem. Int. Ed..

[330] Wang F., Fu H.-R., Kang Y., Zhang J. (2014). New approach towards zeolitic tetrazolate-imidazolate frameworks (ZTIFs) with uncoordinated N-heteroatom sites for high CO_2_ uptake. Chem. Commun..

[331] Chen J.-B., Cui T.-J., Lin G., Wang F., Zhang J. (2019). Sodalite-type metal-organic zeolite with uncoordinated N-sites as potential anticancer drug 5-fluorouracil (5-FU) delivery platform. Inorg. Chem. Commun..

[332] Peng F., Sun Y., Pickard C.J., Needs R.J., Wu Q., Ma Y. (2017). Hydrogen clathrate structures in rare earth hydrides at high pressures: Possible route to room-temperature superconductivity. Phys. Rev. Lett..

[333] Somayazulu M., Ahart M., Mishra A.K., Geballe Z.M., Baldini M., Meng Y., Struzhkin V.V., Hemley R.J. (2019). Evidence for superconductivity above 260 K in lanthanum superhydride at megabar pressures. Phys. Rev. Lett..

[334] Wang H., Tse J.S., Tanaka K., Iitaka T., Ma Y. (2012). Superconductive sodalite-like clathrate calcium hydride at high pressures. Proc. Nat. Acad. Sci. USA.

[335] Fu J., Song T., Liang X., Zhao G., Liu Z. (2020). Room temperature ferromagnetic half metal in Mn doped cluster-assembled sodalite phase of III-N compounds. J. Magn. Magn. Mater..

[336] Nakano T., Ishida Y., Hanazawa A., Nozue Y. (2013). Antiferromagnetic phase transition of K-Rb alloy nanoclusters incorporated in sodalite. J. Korean Phys. Soc..

[337] Nakano T., Suehiro R., Hanazawa A., Watanabe K., Watanabe I., Amato A., Pratt F.L., Nozue Y. (2010). μSR Study on antiferromagnetism of alkali-metal clusters incorporated in zeolite sodalite. J. Phys. Soc. Jpn..

[338] Nakano T. (2020). Antiferromagnetic orderings of alkali-metal nanoclusters arrayed in sodalite crystal studied by μSR and other microscopic probes. J. Comput. Chem. Jpn..

[339] Igarashi M., Nakano T., Goto A., Hashi K., Shimizu T., Hanazawa A., Nozue Y. (2012). NMR property of rubidium loaded sodalite. J. Phys. Chem. Solids.

[340] Nakamura K., Koretsune T., Arita R. (2009). *Ab initio* derivation of the low-energy model for alkali-cluster-loaded sodalites. Phys. Rev. B.

[341] Srdanov V.I., Stucky G.D., Lippmaa E., Engelhardt G. (1998). Evidence for an antiferromagnetic transition in a zeolite-supported cubic lattice of *F* centers. Phys. Rev. Lett..

[342] Madsen G.K.H., Iversen B.B., Blaha P., Schwarz K. (2001). Electronic structure of the sodium and potassium electrosodalites (Na/K)_8_(AlSiO_4_)_6_. Phys. Rev. B.

[343] Tou H., Maniwa Y., Mizoguchi K., Damjanovic L., Srdanov V.I. (2001). NMR studies on antiferromagnetism in alkali-electro-sodalite. J. Magn. Magn. Mater..

[344] Komada N., Westrum E.F., Hemingway B.S., Zolotov M.Y., Semenov Y.V., Khodakovsky I.L., Anovitz L.M. (1995). Thermodynamic properties of sodalite at temperatures from 15 K to 1000 K. J. Chem. Thermodyn..

[345] Schliesser J., Lilova K., Pierce E.M., Wub L., Missimer D.M., Woodfield B.F., Navrotsky A. (2017). Low temperature heat capacity and thermodynamic functions of anion bearing sodalites Na_8_Al_6_Si_6_O_24_X_2_ (X = SO_4_, ReO_4_, Cl, I). J. Chem. Thermodyn..

[346] Sharp Z.D., Helffrich G.R., Bohlen S.R., Essene E.J. (1989). The stability of sodalite in the system NaAlSiO_4_–NaCl. Geochim. Cosmochim. Acta.

[347] Kimura R., Nghia D.T., Wakabayashi J., Elangovan S.P., Ogura M., Okubo T. (2012). Nepheline synthesized from sodalite as diesel-soot combustion catalyst: Structure-property relationship study for an enhanced water tolerance. Bull. Chem. Soc. Jpn..

[348] Pekov I.V., Turchkova A.G., Lovskaya E.V., Chukanov N.V. (2004). Zeolites of Alkaline Massifs.

[349] Hassan I., Antao S.M., Parise J.B. (2004). Sodalite: High-temperature structures obtained from synchrotron radiation and Rietveld refinements. Am. Mineral..

[350] Khajavi S., Sartipi S., Gascon J., Jansen J.C., Kapteijn F. (2010). Thermostability of hydroxy sodalite in view of membrane applications. Microporous Mesoporous Mater..

[351] Henderson C.M.B., Taylor D. (1978). The thermal expansion of aluminate- and .aluminogermanate-sodalites. Mineral. Mag..

[352] Gesing T.M. (2007). Structure and properties of tecto-gallosilicates II. Sodium chloride, bromide and iodide sodalites. Z. Kristallogr..

[353] Gesing T.M., Schmidt B.C., Murshed M.M. (2010). Temperature dependent structural and spectroscopic studies of sodium gallosilicate nitrite sodalite. Mater. Res. Bull..

[354] Leardini L., Martucci A., Cruciani G. (2012). The unusual thermal expansion of pure silica sodalite probed by in situ time-resolved synchrotron powder diffraction. Microporous Mesoporous Mater..

[355] Martucci A., Leardini L., Alberti A. (2011). Negative thermal expansion in trioxane silica sodalite (TRSS). Acta Cryst..

[356] Antao S., Hassan I. (2002). Thermal analyses of sodalite, tugtupite, danalite and helvite. Can. Mineral..

[357] Golovina N.I., Chukanov N.V., Raevskii A.V., Atovmyan L.O. (2000). Prephase state in crystals of Prephase state in 2-bromo-2-nitropropane-1,3-diol: Structural aspects and IR spectra. J. Struct. Chem..

[358] Golovina N.I., Raevskii A.V., Fedorov B.S., Gusakovskaya I.G., Trofimova R.F., Chukanov N.V., Atovmyan L.O. (2001). Experimental study of structure-energy changes in molecules and crystals of 2,2-dinitropropane-1,3-diol caused by temperature variations. J. Solid State Chem..

[359] Golovina N.I., Raevskii A.V., Fedorov B.S., Chukanov N.V., Shilov G.V., Leonova L.S., Tarasov V.P., Erofeev L.N. (2002). Temperature-dependet structure-energetic changes in crystals of compounds with pyly(hydroxymethyl) grouping. J. Solid State Chem..

[360] Zakharov V.V., Chukanov N.V., Larikova T.S., Shilov G.V., Korepin A.G., Pivkina A.N., Monogarov K.A., Korsunskiy B.L., Korchagin D.V., Aldoshin S.M. (2020). Effect of polymorphic phase transitions on stability of energetic compounds. Thermal transformations of 2,4,6-tris(2,2,2-trinitroethylnitramino)-1,3,5-triazine. Russ. Chem. Bull. Int. Ed..

[361] Zakharov V.V., Chukanov N.V., Shilov G.V., Malkov G.V., Larikova T.S., Nedel’ko V.V., Korepin A.G., Korsunskiy B.L. (2021). Phase transformations of 2,4,6-tris(2,2,2-trinitroethylamino)-1,3,5-triazine. Russ. J. Phys. Chem. B.

[362] Robben L., Abrahams I., Fischer M., Hull S., Dove M.T., Gesing T.M. (2018). Low-temperature anharmonicity and symmetry breaking in the sodalite |Na_8_I_2_ |[AlSiO_4_]_6_. Z. Kristallogr..

[363] Depmeier W. (1984). Aluminate sodalite Ca_8_[Al_12_O_24_](WO_4_)_2_ at room temperature. Acta Crystallogr. Sect. C Cryst. Struct. Commun..

[364] Robben L., Wolpmann M., Bottke P., Petersen H., Šehović M., Gesing T.M. (2018). Disordered but primitive gallosilicate hydro-sodalite: Structure and thermal behaviour of a framework with novel cation distribution. Microporous Mesoporous Mater..

[365] Hermeler G., Buhl J.-C., Hoffmann W. (1991). The influence of carbonate on the synthesis of an intermediate phase between sodalite and cancrinite. Catal. Today.

[366] Grader C., Buhl J.-C. (2013). The intermediate phase between sodalite and cancrinite: Synthesis of nano-crystals in the presence of Na_2_CO_3_/TEA and its thermal- and hydrothermal stability. Microporous Mesoporous Mater..

[367] Buhl J.-C. (2017). Synthesis of a sulfate enclathrated zeolite with intermediate framework structure between sodalite and cancrinite. Z. Anorg. Allg. Chem..

[368] Petersen H., Zhao H., Robben L., Kolb U., Gesing T.M. (2019). An average structure model of the intermediate phase between sodalite and cancrinite. Z. Krist. Cryst. Mater..

[369] Heiden F., Nielsen U.G., Warner T.E. (2012). Synthesis and thermal stability of the sodalite Na_6_Zn_2_[Al_6_Si_6_O_24_](SO_4_)_2_ and its reaction with hydrogen. Microporous Mesoporous Mater..

[370] Poltz I., Robben L., Buhl J.-C., Gesing T.M. (2013). Synthesis, crystal structure and temperature-dependent behavior of gallogermanate tetrahydroborate sodalite |Na_8_(BH_4_)_2_|[GaGeO_4_]_6_. Microporous Mesoporous Mater..

[371] Buhl J.-C., Luger S. (1990). The properties of salt-filled sidalites. I. Thermal decomposition reactions of hydroxoborate sodalite. Thermochim. Acta.

[372] Buhl J.-C. (1993). The properties of salt-filled sodalites. Part 3. Synthesis and thermal behaviour of basic and non-basic carbonate enclathrated sodalites. Thermochim. Acta.

[373] Braunbarth C.M., Behrens P., Felsche J., van de Goor G. (1997). Phase transitions and thermal behaviour of silica sodalites. Solid State Ion..

[374] Xiong Y. (2016). Solubility constants of hydroxyl sodalite at elevated temperatures evaluated from hydrothermal experiments: Applications to nuclear waste isolation. Appl. Geochem..

[375] Kuribayashi T., Aoki S., Nagase T. (2018). Thermal behavior of modulated haüyne from Eifel, Germany: In situ high–temperature single–crystal X–ray diffraction study. J. Mineral. Pet. Sci..

[376] Bokiy G.B., Borutskiy B.E. (2003). Minerals V(2): Feldspathoids.

[377] Johnson G.M., Mead P.J., Weller M.T. (2000). Synthesis of a range of anion-containing gallium and germanium sodalites. Microporous Mesoporous Mater..

[378] Nenoff T.M., Harrison W.T.A., Gier T.E., Keder N.L., Zaremba C.M., Srdanov V.I., Nicol J.M., Stucky G.D. (1994). Structural and chemical investigations of Na_3_(ABO_4_)_3_·4H_2_O-Type sodalite Phases. Inorg. Chem..

[379] Newsam J.M., Jorgensen J.D. (1987). Gallosilicate sodalite—further syntheses and structural details. Zeolites.

[380] McCusker L.B., Meier W.M., Suzuki K., Shin S. (1986). The crystal structure of a sodium gallosilicate sodalite. Zeolites.

[381] Chukanov N.V., Pekov S.M.A.I.V. (2023). Infrared spectroscopy as a tool for the analysis of framework topology and extraframework components in microporous cancrinite- and sodalite-related aluminosilicates. Spectrochim. Acta A Mol. Biomol. Spectrosc..

[382] Chukanov N.V. (2014). Infrared Spectra of Mineral Species: Extended Library.

[383] Chukanov N.V., Chervonnyi A.D. (2016). Infrared Spectroscopy of Minerals and Related Compounds.

[384] Ling Z.C., Wang A., Jolliff B.L. (2011). Mineralogy and geochemistry of four lunar soils by laser-Raman study. Icarus.

[385] Chukanov N.V., Rastsvetaeva R.K., Zubkova N.V., Vigasina M.F., Pekov I.V., Zolotarev A.A., Mikhailova J.A., Aksenov S.M. (2024). Spectroscopic characterization of extra-framework hydrated proton complexes with the extremely strong hydrogen bonds in microporous silicate minerals. J. Raman Spectrosc..

[386] Libowitzky E. (1999). Correlation of O–H stretching frequencies and O–H···O hydrogen bond lengths in minerals. Monatsh. Chem..

[387] Zhang S., Cui X., Liu L.-P., Zhang W.-P. (2010). A novel chemosynthetic method for the production of nitrate sodalite. Mater. Lett..

[388] Borhade V., Wakchaure S.G., Dholi A.G. (2010). One pot synthesis and crystal structure of aluminosilicate mixed chloro-iodo sodalite. Indian J. Phys..

[389] Lau C., Brück S., Mai H.-J., Kynast U. (2001). Incorporation of tungsten trioxide into faujasites and sodalites by solid-state reactions. Microporous Mesoporous Mater..

[390] Peng H., Ding M., Vaughan J. (2018). The anion effect on zeolite LTA to sodalite phase transformation. Ind. Eng. Chem. Res..

[391] Warner T.E., Bancells M.M., Lund P.B., Lund F.W., Ravnsbæk D.B. (2019). On the thermal stability of manganese(II) sulfate and its reaction with zeolite A to form the sodalite Na_6_Mn_2_[Al_6_Si_6_O_24_](SO_4_)_2_. J. Solid State Chem..

[392] Beagley B., Henderson C.M.B., Taylor D. (1982). The crystal structures of aluminosilicate-sodalites: X-ray diffraction studies and computer modelling. Mineral. Mag..

[393] Brenchley M.E., Weller M.T. (1994). Synthesis and structures of M_8_[ALSiO_4_]_6_·(XO_4_)_2_, M = Na, Li, K.; X = Cl, Mn sodalites. Zeolites.

[394] Borhade A.V., Kshirsagar T.A., Dholi A.G. (2017). Eco-friendly synthesis of aluminosilicate bromo sodalite from waste coal fly ash for the removal of copper and methylene blue dye. Arab. J. Sci. Eng..

[395] Nielsen N.C., Bildsøe H., Jakobsen H.J., Norby P. (1991). ^7^Li, ^23^Na, and ^27^Al quadrupolar interactions in some aluminosilicate sodalites from MAS n.m.r. spectra of satellite transitions. Zeolites.

[396] Stein A., Ozin G.A., Macdonald P.M., Stucky G.D., Jelinek R. (1992). Silver, sodium halosodalites: Class A sodalites. J. Am. Chem. Soc..

[397] Pierce E.M., Lukens W.W., Fitts J.P., Jantzen C.M., Tang G. (2014). Experimental determination of the speciation, partitioning, and release of perrhenate as a chemical surrogate for pertechnetate from a sodalite-bearing multiphase ceramic waste form. Appl. Geochem..

[398] Mead P.J., Weller M.T. (1995). Synthesis, structure, and characterization of halate sodalites: *M*_8_[AlSiO_4_]_6_(XO_3_)*_x_*(OH)_2−*x*_; *M* = Na, Li, or K.; *X* =Cl, Br, or I. Zeolites.

[399] Murshed M.M., Baer A.J., Gesing T.M. (2008). Isomorphous framework cation substitution in the alumosilicatesodalites: Synthesis, structural and spectroscopic studies of nitrite containing phases. Z. Krist..

[400] Latturner S.E., Sachleben J., Iversen B.B., Hanson J., Stucky G.D. (1999). Covalent guest−framework interactions in heavy metal sodalites: Structure and properties of thallium and silver sodalite. J. Phys. Chem. B.

[401] Baerlocher C., Meier W.M. (1969). Synthese und Kristallstruktur von Tetramethylammonium-Sodalith. Helv. Chim. Acta.

[402] Chong S., Peterson J., Nam J., Riley B., McCloy J. (2017). Synthesis and characterization of iodosodalite. J. Am. Ceram. Soc..

[403] Fejes P., Kiricsi I., Kovács K., Lázár K., Marsi I., Oszkó A., Rockenbauer A., Schay Z. (2002). Incorporation of iron in sodalite structures and their transformation into other iron containing zeolites. Synthesis of Fe-NaA (LTA). Appl. Catal. A Gen..

[404] Paillaud J.-L., Marichal C., Roux M., Baerlocher C., Chézeau J.M. (2005). Tripling of the unit cell volume of the non-centrosymmetric AlPO_4_-SOD after Dehydration: A structural study of a reversible process. J. Phys. Chem. B.

[405] Roux M., Marichal C., Paillaud J.-L., Fernandez C., Baerlocher C., Chézeau J.-M. (2001). Structural investigation by multinuclear solid state NMR and X-ray diffraction of as-synthesized, dehydrated, and calcined AlPO_4_-SOD. J. Phys. Chem. B.

[406] Flanigen E.M., Lok B.M., Patton R.L., Wilson S.T. (1986). Aluminophosphate Molecular Sieves and the Periodic Table.

[407] Wilson S.T., Lok B.M., Messina C.A., Cannan T.R., Flanigen E.M. (1982). Aluminophosphate molecular sieves: A new class of microporous crystalline inorganic solids. J. Am. Chem. Soc..

[408] Han S., Smith J.V., Pluth J.J., Richardson J.W. (1990). Crystal structure of MAPO-20 sodalite; theoretical analysis of three-color ordering of Mg, Al and P in a sodalite unit. Eur. J. Mineral..

[409] Brenchley M.E., Weller M.T. (1992). Synthesis and structure of sulfide aluminate sodalites. J. Mater. Chem..

[410] Ponomarev V.I., Kheiker D.M., Belov N.V. (1971). Crystal structure of tetracalcium trialuminate—The aluminate analog of sodalite. Sov. Phys. Crystallogr..

[411] Dann S.E., Weller M.T. (1998). The preparation, characterisation and structure of Ca_8_[AlO_2_]_12_Te_2_ and Cd_8_[AlO_2_]_12_Te_2_; two new members of the sodalite family. J. Mater. Chem..

[412] Depmeier W. (1988). Structure of cubic aluminate sodalite Ca_8_[Al_12_O_24_](WO_4_)_2_ in comparison with its orthorhombic phase and with cubic Sr_8_[Al_12_O_24_](CrO_4_)_2_. Acta Crystallogr. Sect. B Struct. Sci..

[413] van Smaalen S., Dinnebier R., Katzke H., Depmeier W. (1997). Structural characterization of the high-temperature phase transitions in Ca_8_[Al_12_O_24_](MoO_4_)_2_ aluminate sodalite using X-ray powder diffraction. J. Solid State Chem..

[414] Antao S.M., Hassan I., Parise J.B. (2004). Chromate aluminate sodalite, Ca_8_[Al_12_O_24_](CrO_4_)_2_: Phase transitions and high-temperature structural evolution of the cubic phase. Can. Mineral..

[415] Saalfeld H., Depmeier W. (1972). Silicon-Free Compounds with Sodalite Structure. Krist. Tech..

[416] Dann S.E., Weller M.T. (1996). The structures of strontium tellurite and strontium telluride aluminate sodalites studied by powder neutron diffraction, EXAFS, IR and MAS NMR spectroscopies. J. Mater. Chem..

[417] Depmeier W., Bührer W. (1991). Aluminate sodalites: Sr_8_[Al_12_O_24_](MoO_4_)_2_ (SAM) at 293, 423, 523, 623 and 723 K and Sr_8_[Al_12_O_24_](WO_4_)_2_ (SAW) at 293 K. Acta Crystallogr. Sect. B Struct. Sci..

[418] Depmeier W., Schmid H., Setter N., Werk M.L. (1987). Structure of cubic aluminate sodalite, Sr_8_[Al_12_O_24_](CrO_4_)_2_. Acta Crystallogr. Sect. C Cryst. Struct. Commun..

[419] Többens D.M., Depmeier W. (2001). Superstructure of strontium chromate aluminate sodalite at low temperatures. Z. Krist. Cryst. Mater..

[420] Többens D.M., Depmeier W. (2001). The intermediate phase of strontium chromate aluminate sodalite. Z. Krist. Cryst. Mater..

[421] Scheikowski M., Müller-Buschbaum H. (1993). Zur Kristallchemie der Blei-Lanthanoid-Oxoaluminate. Zur Kenntnis von Pb_2_HoAl_3_O_8_ und Pb_2_LuAl_3_O_8_. Z. Anorg. Allg. Chem..

[422] King R.S.P., Dann S.E., Elsegood M.R.J., Kelly P.F., Mortimer R.J. (2009). The synthesis, full characterisation and utilisation of template-free silica sodalite, a novel polymorph of silica. Chem. Eur. J..

[423] Moteki T., Chaikittisilp W., Shimojima A., Okubo T. (2008). Silica sodalite without occluded organic matters by topotactic conversion of lamellar precursor. J. Am. Chem. Soc..

[424] Koike M., Sakai R., Enomoto S., Mino T., Sugimura N., Gotoh T., Wada H., Shimojima A., Kuroda K. (2020). Encapsulation of Cu nanoparticles in nanovoids of plate-like silica sodalite through interlayer condensation of Cu^2+^ ion-exchanged layered silicate RUB-15. Dalton Trans..

[425] Sato M., Kojima E., Uehara H., Miyake M. (1997). Si,Al solid solution in sodalite: Synthesis, 29Si NMR and X-ray structure. Stud. Surf. Sci. Catal..

[426] Richardson J.W., Pluth J.J., Smith J.V., Dytrych W.J., Bibby D.M. (1988). Conformation of ethylene glycol and phase change in silica sodalite. J. Phys. Chem..

[427] Hong S.B., Camblor M.A., Davis M.E. (1997). Host–guest Interactions in pure-silica and aluminosilicate sodalites containing ethylene glycol as a guest molecule. J. Am. Chem. Soc..

[428] Bibby D.M., Dale M.P. (1985). Synthesis of silica-sodalite from non-aqueous systems. Nature.

[429] Murshed M.M., Gesing T.M. (2008). Gallium substitution in the alumosilicate framework: Synthesis and structural studies of hydro sodalites. Z. Kristallogr..

[430] Johnson G.M., Weller M.T. (1999). A powder neutron diffraction study of lithium-substituted gallosilicate and aluminogermanate halide sodalites. Inorg. Chem..

[431] Murshed M.M., Gesing T.M. (2007). Isomorphous gallium substitution in the alumosilicate sodalite framework: Synthesis and structural studies of chloride and bromide containing phases. Z. Krist..

[432] Borhade A.V., Dholi A.G., Wakchaure S.G. (2010). Synthesis, characterization and crystal structure of gallosilicate perchlorate sodalite. Int. J. Chem..

[433] Borhade A.V., Wakchaure S.G. (2010). Synthesis and characterization of gallosilicate sodalite containing NO_2_^–^ ions. Int. J. Chem. Biol. Eng..

[434] Buhl J.-C., Gesing T.M., Höfs T., Rüscher C.H. (2006). Synthesis and crystal structure of gallosilicate- and aluminogermanate tetrahydroborate sodalites Na_8_[GaSiO_4_]_6_(BH_4_)_2_ and Na_8_[AlGeO_4_]_6_(BH_4_)_2_. J. Solid State Chem..

[435] Borhade A.V., Wakchaure S.G. (2011). Synthesis, crystal structure and characterization of Na_2.02_Ag_5.98_[GaSiO_4_]_6_(NO_2_)_2_ and Na_3.12_K_4.88_[GaSiO_4_]_6_(NO_2_)_2_ sodalites. Indian J. Pure Appl. Phys..

[436] Armstrong J.A., Weller M.T. (2006). New sodalite frameworks; synthetic tugtupite and a beryllosilicate framework with a 3:1 Si:Be ratio. Dalton Trans..

[437] Dann S.E., Weller M.T. (1996). Synthesis and structure of cadmium chalcogenide beryllosilicate sodalites. Inorg. Chem..

[438] Nenoff T.M., Harrison W.T.A., Gier T.E., Stucky G.D. (1991). Room-temperature synthesis and characterization of new ZnPO and ZnAsO sodalite open frameworks. J. Am. Chem. Soc..

[439] Holden M.A., Cubillas P., Attfield M.P., Gebbie J.T., Anderson M.W. (2012). Growth mechanism of microporous zincophosphate sodalite revealed by in situ atomic force microscopy. J. Am. Chem. Soc..

[440] Gier T.E., Harrison W.T.A., Stucky G.D. (1991). The synthesis and structure of some new sodalites: The lithium haloberyllophosphates and -arsenates. Angew. Chem. Int. Ed..

[441] Wiebcke M., Sieger P., Felsche J., Engelhardt G., Behrens P., Schefer J. (1993). Sodium aluminogermanate hydroxosodalite hydrate Na_6+x_[Al_6_Ge_6_O_24_](OH)*_x_*·*n*H_2_O (*x* = 1.6, *n* = 3.0): Synthesis, phase transitions and dynamical disorder of the hydrogen dihydroxide anion, H_3_O_2_?, in the cubic high-temperature form. Z. Anorg. Allg. Chem..

[442] Fleet M.E. (1989). Structures of sodium alumino-germanate sodalites [Na_8_(Al_6_Ge_6_O_24_)A_2_, A = Cl, Br, I]. Acta Crystallogr. Sect. C Cryst. Struct. Commun..

[443] Belokoneva E.L., Demyanets L.N., Uvarova T.G., Belov N.V. (1982). Crystal structure of Ge Sodalite Na_8_Al_6_Ge_6_O_24_(OH)_2_. Kristallografiya.

[444] Borhade A.V., Dholi A.G., Tope D.R., Wakchaure S.G. (2012). Synthesis and characterization of perchlorate enclathrated aluminogermanate sodalite and its potassium and silver derivatives. J. Anal. Sci. Technol..

[445] Bachmann S., Buhl J.-C. (1999). Crystallization, characterization and structure of nitrite aluminogermanate sodalite Na_8_[AlGeO_4_]_6_(NO_2_)_2_. Microporous Mesoporous Mater..

[446] Camblor M.A., Lobo R.F., Koller H., Davis M.E. (1994). Synthesis and characterization of zincosilicates with the SOD topology. Chem. Mater..

[447] Feng P., Bu X., Stucky G.D. (1997). Hydrothermal syntheses and structural characterization of zeolite analogue compounds based on cobalt phosphate. Nature.

[448] Bu X., Gier T.E., Feng P., Stucky G.D. (1998). Template control of framework topology and charge in new phosphate- and arsenate-based sodalite analogs. Microporous Mesoporous Mater..

[449] Parnham E.R., Morris R.E. (2006). The ionothermal synthesis of cobalt aluminophosphate zeolite frameworks. J. Am. Chem. Soc..

[450] Feng P., Zhang T., Bu X. (2001). Arsenate Zeolite analogues with 11 topological types. J. Am. Chem. Soc..

[451] Poltz I., Robben L., Buhl J.-C. (2010). Synthesis and characterization of B(OH)_4_·H_2_O enclathered gallogermanate sodalite. Acta Crystallogr. Sect. A.

[452] Bu X., Feng P., Gier T.E., Zhao D., Stucky G.D. (1998). Hydrothermal synthesis and structural characterization of zeolite-like structures based on gallium and aluminum germanates. J. Am. Chem. Soc..

[453] Vaughan D.E.W., Yennawar H.P., Perrotta A.J. (2006). Synthesis and structure of large optically clear crystals of gallogermanate sodalite. Cryst. Growth Des..

[454] Poltz I., Robben L., Buhl J.-C., Gesing T.M. (2015). Synthesis, crystal structure and temperature-dependent properties of gallogermanate nitrite sodalite |Na_8_(NO_2_)_2_|[GaGeO_4_]_6_. Microporous Mesoporous Mater..

[455] Fouassier C., Levasseur A., Joubert J.C., Muller J., Hagenmuller P. (1970). Les Systémes B_2_O_3_·MO·MS boracites (M = Mg, Mn, Fe, Cd) et Sodalites (M = Co, Zn). Z. Anorg. Allg. Chem..

[456] Pan R., Cheng J.-W., Yang B.-F., Yang G.-Y. (2017). CsB*_x_*Ge_6–*x*_O_12_ (*x* = 1): A zeolite sodalite-type borogermanate with a high Ge/B ratio by partial boron substitution. Inorg. Chem..

[457] Zheng S.-T., Wu T., Zuo F., Chou C., Feng P., Bu X. (2012). Mimicking zeolite to its core: Porous sodalite cages as hangers for pendant trimeric M_3_(OH) Clusters (M = Mg, Mn, Co, Ni, Cd). J. Am. Chem. Soc..

[458] Zhang T.-Z., Zhang Z.-M., Lu Y., Fu H., Wang E.-B. (2013). Expansion of sodalite-type metal–organic frameworks with heterometallic metal–oxo cluster and its cation exchange property. CrystEngComm.

[459] Beldon P.J., Fábián L., Stein R.S., Thirumurugan A., Cheetham A.K., Fris T. (2010). Rapid room-temperature synthesis of zeolitic imidazolate frameworks by using mechanochemistry. Angew. Chem. Int. Ed..

[460] Martinez V., Karadeniz B., Biliškov N., Lončarić I., Muratović S., Žilić D., Avdoshenko S.M., Roslova M., Popov A.A., Užarević K. (2020). Tunable fulleretic sodalite MOFs: Highly efficient and controllable entrapment of C_60_ fullerene via mechanochemistry. Chem. Mater..

[461] Karagiaridi O., Lalonde M.B., Bury W., Sarjeant A.A., Farha O.K., Hupp J.T. (2012). Opening ZIF-8: A catalytically active zeolitic imidazolate framework of sodalite topology with unsubstituted linkers. J. Am. Chem. Soc..

[462] Gross A.F., Sherman E., Vajo J.J. (2012). Aqueous room temperature synthesis of cobalt and zinc sodalite zeolitic imidizolate frameworks. Dalton Trans..

